# De novo design and directed folding of disulfide-bridged peptide heterodimers

**DOI:** 10.1038/s41467-022-29210-x

**Published:** 2022-03-22

**Authors:** Sicong Yao, Adam Moyer, Yiwu Zheng, Yang Shen, Xiaoting Meng, Chong Yuan, Yibing Zhao, Hongwei Yao, David Baker, Chuanliu Wu

**Affiliations:** 1 Department of Chemistry, College of Chemistry and Chemical Engineering, The MOE Key Laboratory of Spectrochemical Analysis and Instrumentation, State Key Laboratory of Physical Chemistry of Solid Surfaces, Xiamen University, Xiamen, 361005 P.R. China; 2 Department of Biochemistry and Institute for Protein Design, University of Washington, Seattle, WA 98195 USA; 3 Institute of Molecular Enzymology, School of Biology and Basic Medical Sciences, Soochow University, Suzhou, 215123 P.R. China

**Keywords:** Peptides, Protein design, Synthetic biology

## Abstract

Peptide heterodimers are prevalent in nature, which are not only functional macromolecules but molecular tools for chemical and synthetic biology. Computational methods have also been developed to design heterodimers of advanced functions. However, these peptide heterodimers are usually formed through noncovalent interactions, which are prone to dissociate and subject to concentration-dependent nonspecific aggregation. Heterodimers crosslinked with interchain disulfide bonds are more stable, but it represents a formidable challenge for both the computational design of heterodimers and the manipulation of disulfide pairing for heterodimer synthesis and applications. Here, we report the design, synthesis and application of interchain disulfide-bridged peptide heterodimers with mutual orthogonality by combining computational de novo designs with a directed disulfide pairing strategy. These heterodimers can be used as not only scaffolds for generating functional molecules but chemical tools or building blocks for protein labeling and construction of crosslinking hybrids. This study thus opens the door for using this unexplored dimeric structure space for many biological applications.

## Abstract (web summary)

Peptide heterodimers are prevalent in nature, which are not only functional macromolecules but molecular tools for chemical and synthetic biology. Here the authors report de novo design and directed folding of peptide heterodimers crosslinked through multiple disulfide bonds, which can be explored as chemical tools for orthogonal labeling of proteins and preparing protein hybrids.

## Introduction

Peptide heterodimers represent a unique structure space that is modularly tunable for acquiring useful functions^1–4^. They can be exploited as modular building blocks for constructing functional assemblies and molecular tools for chemical and synthetic biology^5–10^. The formation of peptide heterodimers is usually driven by noncovalent interactions such as hydrophobic interactions and hydrogen bonding with limited affinity, which could cause the dissociation and concentration-dependent nonspecific aggregation of the loosely assembled heterodimers^11–15^. Interchain disulfide bonding could strengthen the interactions and assist the heterodimer formation^16–20^. However, achieving precise intermolecular disulfide pairing is still a great challenge for both chemical synthesis and computational de novo design due to competitions of correct pairing over mismatched inter-/intra-molecular disulfides^19–24^, although tremendous advance has been made for achieving precise intramolecular disulfide pairing^25–27^. Incorporation of disulfide bonds into peptide heterodimers could enable access to a broad space of sequence, structure and function that cannot be explored by noncovalent heterodimers. Solution of this challenge in both de novo design and synthesis would enable the use of covalent hetero-pairing of peptides as a unique structural modality for broad applications, such as development of functional heterodimers, specific and reversible labeling of proteins, and constructions of peptide/protein hybrids and assemblies.

**Fig. 1:**
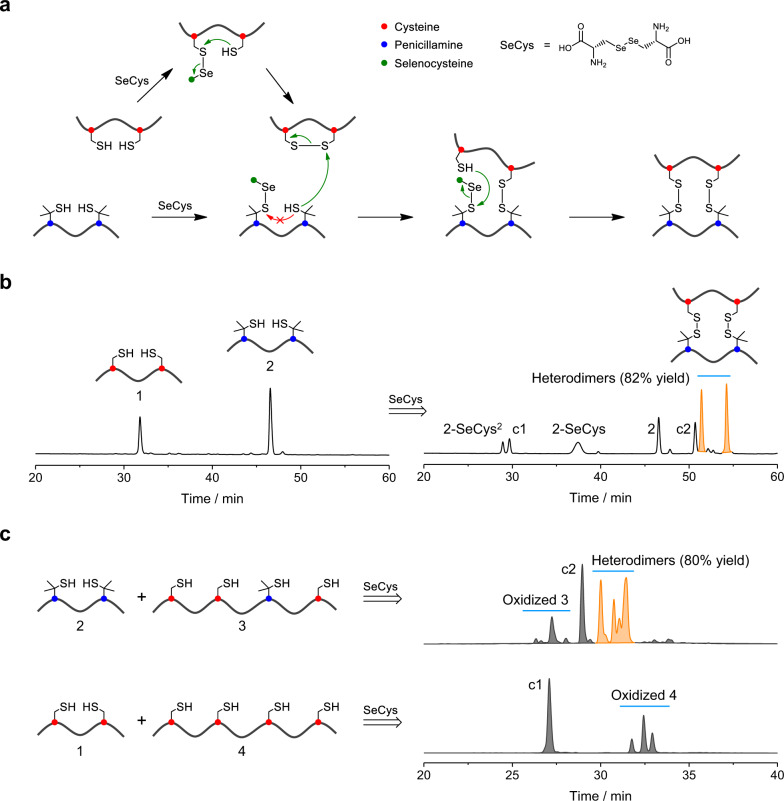
Interchain disulfide pairing chemistry for peptide heterodimerizations. **a** SeCys-mediated thiol-disulfide exchanges between Cys and Pen residues to direct the heterodimerization of peptides with multiple disulfide bonds. **b** HPLC chromatograms showing the oxidation of 1 (0.1 mM; sequence: WGCGKG_3_CG) and 2 (0.2 mM; sequence: WGPenGKG_3_PenG) in the presence of SeCys (0.05 mM) in a phosphate buffer (100 mM, pH 7.4); left: before oxidation, right: after 24 h oxidation. Other peaks in HPLC: 2-SeCys, peptide 2 linked with one SeCys; 2-SeCys^2^, peptide 2 linked with two SeCys; c1: disulfide-cyclic 1; c2, disulfide-cyclic 2. **c** HPLC chromatograms showing the oxidation of 2 (0.2 mM) and 3 (0.1 mM; sequence: WGCG_2_KG_2_CGKG_3_PenGKG_3_CGW) in a phosphate buffer (100 mM, pH 7.4) containing 0.05 mM SeCys and the oxidation of 4 (0.1 mM; sequence: WGCG_2_KG_2_CGKG_3_CGKG_3_CGW) and 1 (0.2 mM) under the same condition. Source data are provided as a Source Data file.

**Fig. 2:**
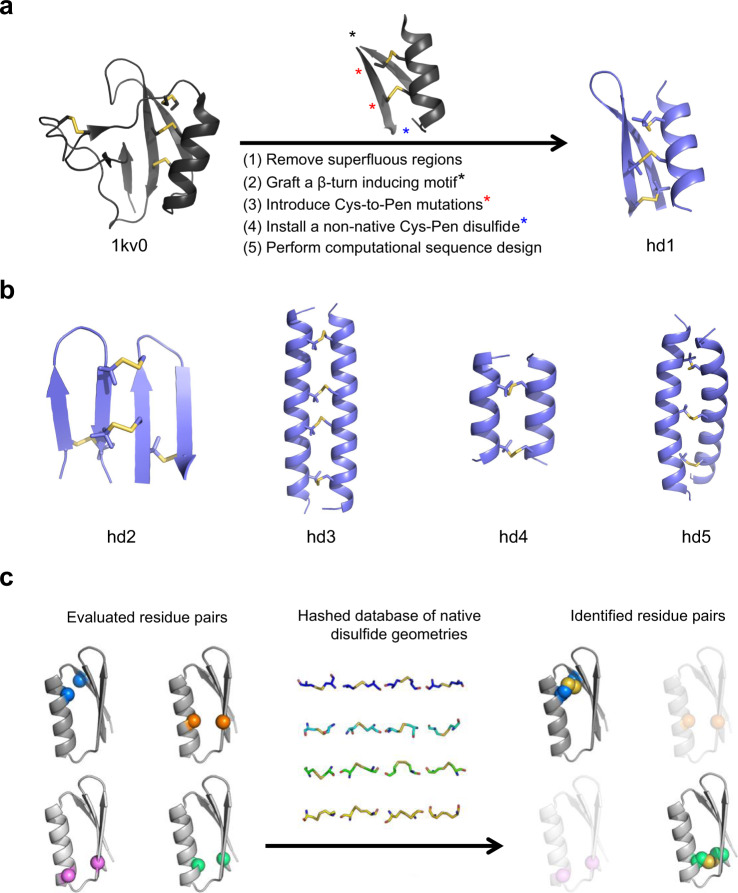
Design principle for structurally ordered peptide heterodimers. **a** Typical workflow of designing peptide heterodimers from disulfide-rich mini-proteins (e.g., from 1kv0 to hd1). **b** Designed heterodimers (hd2‒hd5) crosslinked through 2‒4 interchain disulfide bonds. Sequences of these peptides were given in Supplementary Table 1. **c** Schematic representation of the RPX method for the identification and placement of disulfides within protein/peptide scaffolds. PDB output files for these designed peptides are provided in a Supplementary file.

Herein we report a general strategy for the design and synthesis of disulfide-bridged peptide heterodimers with mutual orthogonality by combining directed disulfide pairing chemistry with computational de novo designs. The directed disulfide pairing chemistry relies on the efficient formation of multiple interchain cysteine (Cys)–penicillamine (Pen) disulfide bonds in the presence of selenol-l-cystine (SeCys) (Fig. 1). Computational designs enable structurally compatible incorporation of this disulfide pairing chemistry in design models (Fig. 2), generating structurally distinct dimeric peptides with orthogonal association properties and high synthetic yields. The peptide heterodimerizations were further applied for specific, orthogonal and reversible labeling of proteins.

## Results and discussion

### Interchain disulfide pairing chemistry

We reported previously that the orthogonal Cys–Pen disulfide pairing can be used to direct the monomer folding of disulfide-rich peptides^28–30^. However, it remains a great challenge to direct the interchain disulfide formation because of the difficulty in suppressing the proximity-induced formation of intrachain disulfides. Herein we discovered that a pair of disordered peptides with two Cys and Pen residues, respectively, can be oxidized to form heterodimers containing two intermolecular Cys–Pen disulfides under the assistance of a special oxidant—SeCys (Fig. 1a). When the two-Cys containing peptide (1) was oxidized in the buffer, we only observed the formation of the close-loop monomer (c1) without dimerization (Supplementary Fig. 1). As the second peptide with two Pen residues (2) was added, we obtained a small amount (~10% yield) of heterodimers in addition to the close-loop monomer c1 and c2 (Supplementary Fig. 2). Interestingly, when SeCys was present in the mixture, we obtained the heterodimers as major products (82% yield; Fig. 1b and Supplementary Fig. 3). Diselenides are often used to promote the oxidative folding of proteins because of their unique thermodynamic properties^31^. Mechanistic analysis reveals that the heterodimer formation results from the different modes of thiol–diselenide exchanges taking place on the two peptides (1 and 2) (Fig. 1a). Thiol–diselenide exchange of 1 with SeCys leads to rapid formation of the mixed 1-SeCys, followed by immediate ejection of SeCys through intramolecular ring closure to form c1. In contrast, reaction of 2 with SeCys leads to formation of 2-SeCys as relatively stable intermediates due to the steric hindrance from the two methyl groups adjacent to the sulfur atom^28^. 2-SeCys can then react intermolecularly with c1 to initiate the dimerization to form the final heterodimers (Fig. 1a). We found further that this directed disulfide pairing strategy can also be used to promote dimerization of peptides with more Cys and Pen residues (Fig. 1c), albeit with the formation of more heterodimeric isomers. For instance, oxidation of 2 and 3 containing one Pen and three Cys residues in the presence of SeCys leads to a high yield formation of heterodimers (80% yield), whereas only monomers were obtained from the control peptides 1 and 4, in which 4 contains four Cys residues (Fig. 1c and Supplementary Figs. 4 and 5).

### Design principle for structurally ordered peptide heterodimers

We then sought to apply this disulfide pairing principle on structure designing. First, we identified previously described (de novo and native) disulfide-rich mini-proteins which could be split into two chains with undestroyed disulfides and could accommodate the required Cys-to-Pen mutations. We found that the disulfide-rich region of the native structure, 1kv0, fits this criterion well^32^. After removing the superfluous regions and joining the remained β-strands by a simple β-turn inducing motif, Gly-Pro, we obtained a heterodimeric complex of an α-helix and a β-hairpin with two interchain disulfides (Fig. 2a). Computational modeling confirmed that the Cys-to-Pen mutations on the β-hairpin could be accommodated. We further installed a non-native Cys–Pen disulfide between the termini of the β-hairpin to constrain structure via macrocyclization, as well as to challenge the robustness of the chemistry to extra intrachain disulfide pairing. Lastly, we performed structure-based computational sequence design to improve the folding of the complex. Similar protocols were also applied on disulfide-rich mini-proteins designed previously by our lab^33^ and native insulin family peptides as well^23^. However, this strategy was not effective for these peptides. These split peptides formed monomers or a mixture of monomers and non-native dimers. Given that the folding of these peptides largely relies on hydrophobic interactions, we suspect that the exposed hydrophobic residues of the split peptide chains induce some incompetent intermediate states which cannot reach the intended heterodimeric structure.

From this point, we sought to identify more peptide chains that relied on crosslinking interactions in lieu of typical hydrophobic interactions for folding. Three complexes were identified, which exhibit defined structures without burying substantial non-polar surface area (Fig. 2b): (1) the antiparallel β-hairpin heterodimer (hd2), (2) the antiparallel 11/3 coiled coil heterodimers (hd3 and hd4), and (3) the parallel 7/2 coiled coil heterodimer (hd5). The installation of disulfides in the hairpins was guided by the observation that inter-strand disulfides tend to form at the non-hydrogen bonding pairs of β-sheets^34^. The identification of compatible disulfide sites of coiled coils was automated using a hash-based residue pair transform (RPX) approach (Fig. 2c)^35^.

### Designed peptide heterodimers

**Fig. 3:**
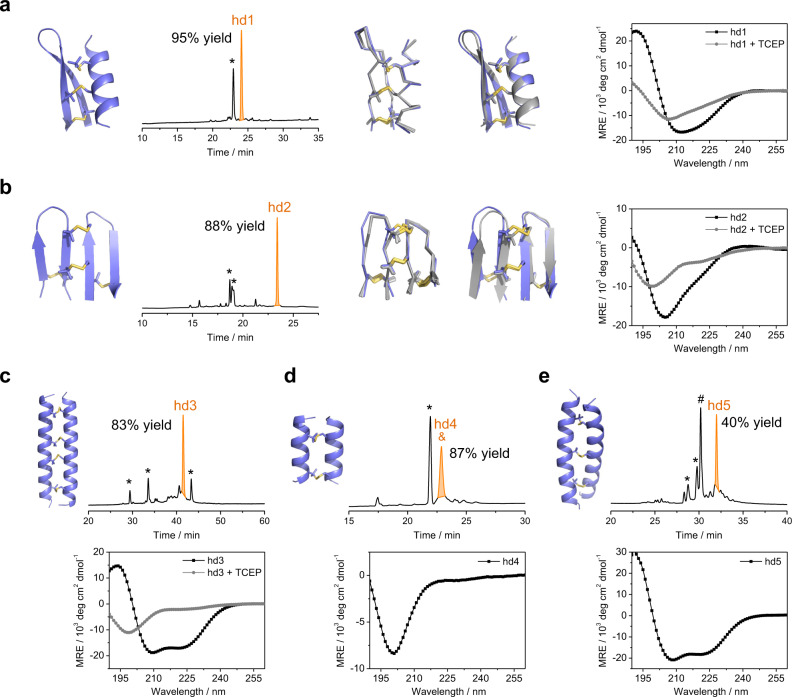
Synthesis and characterizations of the designed peptide heterodimers. **a**–**e** Cartoon representations of design models; C_α_ traces of NMR ensembles (gray) and cartoon representation of the lowest energy conformer of each NMR ensemble (gray) aligned to the design model (blue slate); HPLC chromatograms showing the dimeric folding of these designed heterodimers (concentration of Cys- and Pen-bearing monomers: 100 and 200 μM respectively; * denotes the oxidized Pen-bearing monomers of hd1‒hd5, which are excess relative to the Cys-bearing monomers in the folding buffers); & a peak containing both the correctly-paired antiparallel heterodimer (hd4) and the undesired parallel heterodimer; # denotes the undesired antiparallel heterodimer. CD spectra of the heterodimers were recorded in water at room temperature. TCEP was added to record CD spectra of the reduced monomers (hd1–hd3). Source data are provided as a Source Data file.

These designed heterodimers (hd1‒hd5) were then examined experimentally. We synthesized the peptide monomers using microwave-assisted solid-phase peptide synthesis. hd1–hd3 can be obtained in high yields (>80%) by directly oxidizing peptide monomers in phosphate buffers containing SeCys (Fig. 3a–c and Supplementary Figs. 6–8; monitored by HPLC). The yields of the heterodimers were significantly decreased when the oxidant SeCys in buffers was replaced with oxidized glutathione (GSSG) (Supplementary Figs. 9–11). In addition, no formation of heterodimers was observed from control peptides, in which Pen residues were replaced with Cys residues (Supplementary Figs. 12–14). These results stress the important role of both SeCys and Pen residues in directing the oxidative folding. The secondary structures of these heterodimers (isolated using HPLC) were determined using CD spectroscopy (Fig. 3a–c), indicating the formation of relevant α-helical and β-stranded structures. The interchain disulfide bonds are important for the structural stability as reduced monomers are unstructured (Fig. 3a–c). In addition, we found that the designed heterodimers were very stable to thermal and chemical denaturation (Supplementary Figs. 15 and 16). hd4 and hd5 can also be obtained directly from the dimeric folding of the peptide monomers in redox buffers (Fig. 3d and e and Supplementary Figs. 17–21), though dimeric isomers with incorrect disulfide pairing formed as well (identified by trypsin-digestion LC–MS analysis, Supplementary Figs. 19–21). CD spectra indicate a flexible and α-helical structure for hd4 and hd5, respectively (Fig. 3d and e).

We determined the NMR structures of hd1 and hd2 by using ^1^H, ^1^H distance constraints derived from 2D ^1^H, ^1^H NOESY spectra (PDB IDs: 7FB8, 7FBA; Supplementary Figs. 22–25 and Tables 2 and 3). The NMR structures closely match the design models, with a mean all-atom r.m.s.d. of 2.049 (hd1) and 2.224 (hd2) Å to the lowest-energy NMR structure (Fig. 3a and b). In addition, though neither the NMR characterization nor the crystallization of hd3 was successful, trypsin-digestion LC–MS analysis and CD spectra clearly demonstrated the successful disulfide pairing and folding of hd3 (Fig. 3c and Supplementary Fig. 26). These results, taken together with the previous HPLC and CD characterizations, demonstrated that our computational design approach enables the design of stable and easy-to-fold peptide heterodimers with multiple intermolecular disulfide bonds.

### Orthogonality of peptide heterodimerizations

**Fig. 4:**
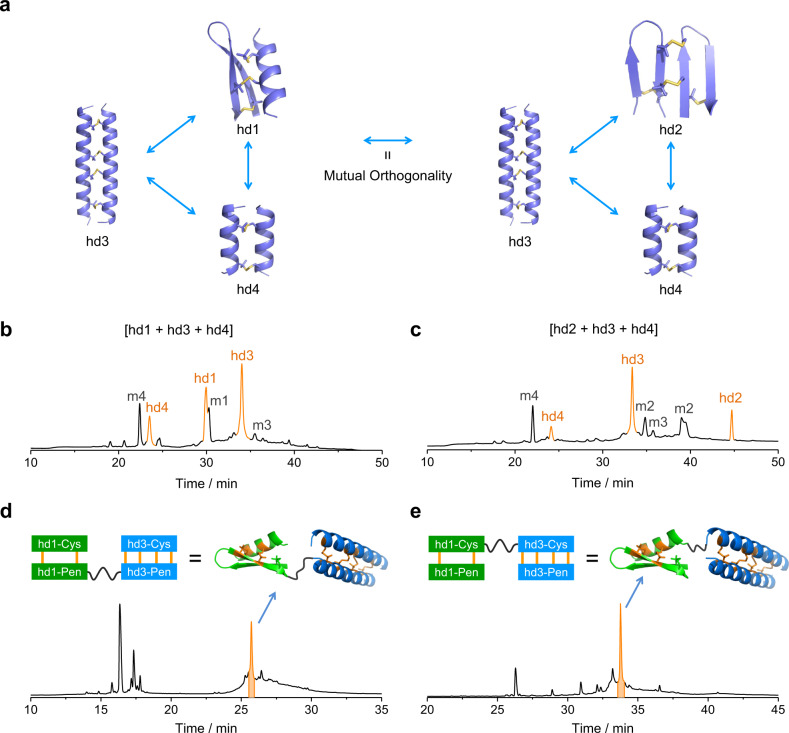
Orthogonality of peptide heterodimerizations. **a** Excellent orthogonality was observed between each two heterodimers except for the orthogonality between hd1 and hd2 (Supplementary Figs. 27‒32). **b** and **c** Excellent orthogonality was observed in the formation of three heterodimers (**b**: hd1/hd3/hd4; **c**: hd2/hd3/hd4). m1‒m4 denote the oxidized products of the excess Pen-bearing monomers of hd1‒hd4, respectively. **d** and **e** Oxidation-induced dimerization leading to formation of the tandem heterodimers: hd1 and hd3 were connected with a flexible linker containing a tobacco etch virus (TEV) protease cleavage site (ENLYFQG). Source data are provided as a Source Data file.

Disulfide-bridged peptide heterodimers represent a unique class of structure space that can be exploited for protein labeling and design of functional proteins or assemblies. Orthogonality of heterodimeric pairing is a desirable property for these applications^5,10^. We first examined the pair-wise orthogonality between hd1–hd4 (Fig. 4a). Reduced monomers from two different heterodimers were mixed and oxidized in redox buffers. The resulting mixtures were analyzed using HPLC and mass spectrometry. Encouragingly, all designed heterodimers were recovered in high yields in the reaction mixtures (Supplementary Figs. 27–32; note that other peaks in HPLC chromatograms come from the excess Pen-bearing monomers (~2:1 ratio) relative to the Cys-bearing counterparts), and no incorrectly paired dimers were observed except for the mixture of hd1 and hd2, in which the α-helical chain of hd1 bearing two cysteines can pair with the β-stranded chain of hd2 with three Pen residues (Supplementary Fig. 32). This is very likely due to the similar structure of the two Pen-bearing β-strands in hd1 and hd2. We next tested whether this orthogonality preserves when three pairs of monomers were mixed to allow oxidation (Fig. 4b, c). Again, the designed heterodimers can be recovered without obvious formation of incorrectly paired dimers (Fig. 4b, c and Supplementary Figs. 33 and 34).

We then examined the potential of forming more complex oligomers using the existing heterodimeric building blocks. One monomer from each of two different heterodimers (e.g., hd1 and hd3) was linked through a tobacco etch virus (TEV) protease-cleavable peptide chain^36^. Oxidation of the linked peptides with the other two monomers in buffers yields the correctly paired heterotrimers as identified by HPLC and protease-digestion LC–MS characterizations (Fig. 4d and e and Supplementary Figs. 35–38). The crosslinked heterotrimers can be dissociated by treating with either reducing reagents or TEV protease (Supplementary Figs. 36 and 38). This approach provides a way to develop induced dimerization and dissociation systems with redox and proteolytic responsiveness.

### Potential applications of peptide heterodimerizations

**Fig. 5:**
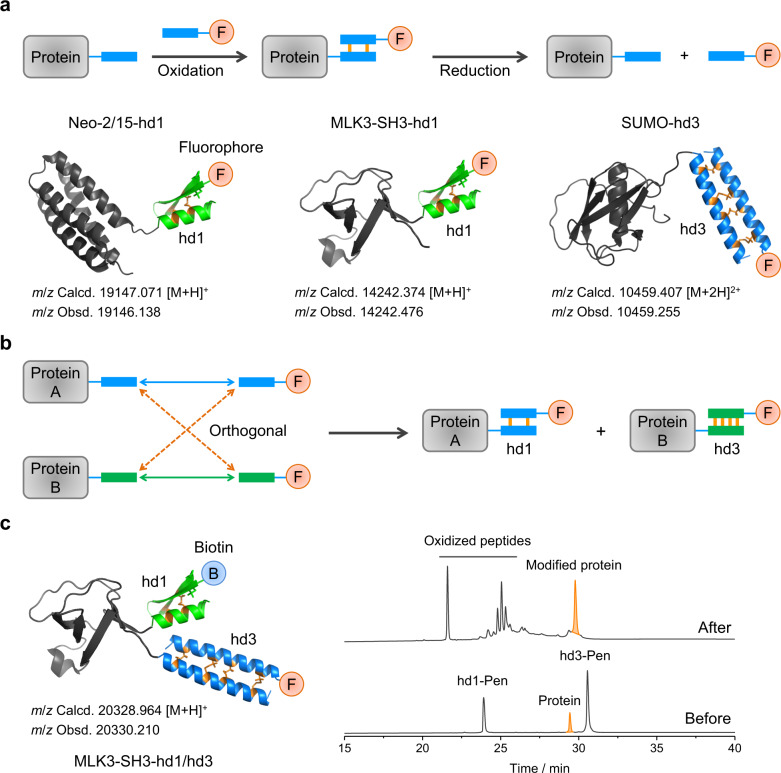
Potential applications of peptide heterodimerizations. **a** Labeling of proteins (Neo-2/15, MLK3-SH3, and SUMO; Supplementary Table 6) with functional molecules (e.g., fluorescent probes) through the heterodimerization. **b** Schematic illustration of the labeling of two different proteins simultaneously through orthogonal peptide heterodimerizations (HPLC chromatograms showing the formation of the relevant labeled proteins were given in Supplementary Figs. 43 and 44). **c** Double labeling of a protein through two orthogonal peptide heterodimerizations, and HPLC chromatograms showing the formation of the modified protein (MLK3-SH3-hd1/hd3; Supplementary Fig. 45). hd1-Pen and hd3-Pen represent the biotin-conjugated Pen-bearing monomer of hd1 and the fluorescein-conjugated Pen-bearing monomer of hd3, respectively. Other peaks besides the modified protein come from oxidation of the excess Pen-bearing peptides. Source data are provided as a Source Data file.

We next explored the disulfide-mediated peptide heterodimerizations for protein labeling and functionalization (Fig. 5). Chemical labeling can greatly expand protein functions. Compared to noncovalent peptide heterodimerizations^1,5^, interchain disulfide bonds can provide the final hybrid protein products with merits of stability and redox responsiveness. Three model proteins (Neo-2/15^37^, SH3 domain of MLK3 (MLK3-SH3), and small ubiquitin-like modifier (SUMO); Supplementary Table 4) were first expressed in recombinant forms with the hd1- or hd3-derived Cys-bearing monomer at the C-termini. Then, the purified protein and the relevant Pen-bearing monomer modified with fluorescein were mixed in the SeCys-containing buffer to allow heterodimer formation. All the three proteins can be efficiently modified through the heterodimerizations as characterized by HPLC, SDS–PAGE, and MALDI-TOF MS (Fig. 5a and Supplementary Figs. 39–42). Encouraged by these findings, we further examined the feasibility of modifying two different proteins simultaneously using two orthogonal peptide heterodimers (Fig. 5b). SUMO with a hd3 monomer tag and Neo-2/15 or MLK3-SH3 with a hd1 monomer tag were mixed in the reaction buffer for the orthogonal labeling. The correctly paired heterodimers can be obtained as major products, indicating the high efficiency of the orthogonal heterodimerizations (Supplementary Figs. 43 and 44). Finally, we explored the possibility of modifying one protein with two different functional modules through orthogonal heterodimerizations (Fig. 5c). We expressed and purified the recombinant MLK3-SH3-hd1/hd3 with the Cys-bearing monomer sequences of hd3 and hd1 at the N- and C-terminus, respectively (Supplementary Table 4). A TEV protease cleavage site was inserted between the terminal peptides and MLK3-SH3 to facilitate the identification of the desired final hybrid product through proteolytic digestion HPLC and mass spectral analysis. To our delight, the protein can be efficiently bonded with Pen-bearing monomers of hd1 and hd3 labeled with biotin and fluorescein, respectively (Fig. 5c and Supplementary Fig. 45). These results, though preliminary, still demonstrated broad applicability and robustness of disulfide-bridged orthogonal heterodimerizations for protein labeling and constructions of complex hybrids or assemblies.

Most protein–protein or protein–peptide complexes are reversible and dynamic. Peptide heterodimers stabilized through interchain disulfide bonds can be more stable, and nature has taken advantages of this strategy to produce many peptide heterodimers to execute important functions (e.g., insulin, relaxin, and insulin-like hormones). However, owing to the challenge of manipulating interchain disulfide pairing, this unique structure space for protein engineering is underexplored. This is not surprising, because even the natural protein folding machinery is incapable of handling interchain disulfide pairing; and insulin and insulin-like heterodimeric peptides are produced in our bodies from well-folded single chain precursors. In this work, we developed a unique, efficient, and convenient interchain disulfide pairing strategy to direct peptide heterodimerizations. By combining the disulfide pairing chemistry with de novo protein design, we can exploit the dimeric structure space for designing peptide heterodimers with new structures. These peptide heterodimers can be used as scaffolds for generating functional entities. Moreover, we have generated a set of heterodimers with mutual orthogonality which can serve as general chemical tools or building blocks for protein labeling and construction of functional hybrids. We believe that this unexplored dimeric structure space would provide solutions for many applications such as protein assembly and functionalization, vaccine design, drug delivery, and development of dimeric peptide therapeutics.

## Methods

### Materials and instruments

Selenol-l-cystine (SeCys) was purchased from J&K Chemical (Guangzhou, China). Reduced glutathione (GSH), oxidized glutathione (GSSG), dithiothreitol (DTT), tris (2-carboxyethyl) phosphine (TCEP), N,N-diisopropylethylamine (DIEA), ethyl cyanoglyoxylate-2-oxime (Oxyma), N,N′-diisopropylcarbodiimide (DIC), ethanedithiol (EDT), and piperidine were purchased from Energy Chemical (Shanghai, China). Acetonitrile (ACN), trifluoroacetic acid (TFA), and trypsin were purchased from Sigma-Aldrich (Beijing, China). Guanidine hydrochloride (Gn·HCl) was purchased from Sangon Biotech (Shanghai, China). 4-Mercaptophenylactic acid (MPAA) was purchased from Alfa Aesar (Beijing, China). Thioanisole was purchased from TCI (Shanghai, China). Rink-amide MBHA resins, 2-chlorotrityl chloride resins and Fmoc-protected amino acids were purchased from GL Biochem (Shanghai, China). Other conventional reagents were purchased from Sinopharm Chemical Reagent (Beijing, China). All reagents were analytical grade at least and used without further purification. Ultrapure water (18.0 MΩ/cm) was used throughout the experiments.

All peptides were synthesized on a CEM Liberty Blue automatic microwave peptide synthesizer. Waters high-performance liquid chromatography (HPLC) and SHIMADZU HPLC were used to purify and analyze peptides and proteins. Bruker Esquire 3000 plus ion trap ESI mass spectrometry, Bruker autoflex maX MALDI-TOF mass spectrometer and Bruker Impact II QqTOF mass spectrometer were used to identify peptides and proteins. HITACHI U-3900H UV–Vis spectrophotometer was used to determine the concentration of peptides and proteins. Jasco J-810 circular dichroism (CD) spectrometer was used for recording CD spectra. Proteins were purified using a SuperdexTM 75 10/300 GL column (GE Healthcare) on a fast protein liquid chromatography (FPLC). NMR experiments were performed at 298 K on Bruker AVANCE III 850 MHz equipped with a cryogenic triple-resonance probe.

### Computation placement of disulfides into designed structures

A database of 30,000 example disulfide geometries was acquired from the PDB database filtering for high-resolution structures (<2.0 Å resolution). The 30,000 example disulfide geometries were utilized to generate a hash database. To improve coverage of the example geometries, all of the example disulfides from the PDB were randomly perturbed 100 times up to 5 Å on the sidechain degrees of freedoms. Once the large set of example disulfides was generated, the relative transformation from backbone-to-backbone coordinates was calculated and hashed using the same hash function in previous work. The cartesian resolution parameter was 1.0 Å and the angular resolution parameter was 15.0°. The hashed transformation was saved as a key in a dictionary and the corresponding rotamers of the cysteine residues were stored as the associated value. This database can be quickly queried to identify residue pairs in peptides and proteins that can accommodate a disulfide bond by calculating all of the hashed residue-to-residue transformations of the design peptide/protein structure and comparing those values to the keys stored in the database. When a hashed residue pair transformation is found in the database, the associated cysteine rotamers can be placed into design peptide/protein structure. The placed disulfide will be close to optimal geometry, but a minimization with the newly placed disulfide bond is necessary to optimize the structure. An implementation of this protocol with instructions for installation can be found at: https://github.com/atom-moyer/stapler.

### Computational design of peptide heterodimers

Various native and non-native scaffolds were evaluated with the hash-based disulfide placement protocol. After disulfides were placed into the potential scaffolds, the structures were prepared for design by splitting the structures into hetero-dimeric structures by splitting the monomeric chains into two separate structures. The penicillamine residues were manually mutated to obey the synthetic restrictions of the Cys–Pen pairing and ensure that the bulkier Pen residue could be structurally accommodated. The sequences were optimized using Rosetta Scripts and PyRosetta with the goal of introducing favorable sidechain interactions across the heterodimer interfaces. The Rosetta protocol focuses on introducing hydrogen bonding interactions across the interface to improve specificity. A representative script and associated parameter files used to design the peptides can be found in a Supplementary file.

### Peptide synthesis and purification

All peptides were synthesized using Fmoc solid-phase peptide synthesis method on a CEM Liberty Blue automatic microwave peptide synthesizer (peptide sequences were provided in Supplementary Table 1). Coupling of Fmoc-protected amino acids to the resins (0.05 mmol scale) was performed in the reaction vessel using standard coupling methods. In general, coupling reactions were performed with a 5-fold excess of Fmoc-protected amino acids, 0.5 mmol DIC, and 0.5 mmol Oxyma in DMF. After each coupling step, Fmoc groups were removed using 20% piperidine in DMF. The synthesized peptides were cleaved from the resins by treating the peptidyl resins with a cleavage cocktail (TFA/thioanisole/EDT/H_2_O/phenol in a volume ratio of 87.5/5.0/2.5/2.5/2.5) for 3.0 h on a shaker at 37 °C. After that, the cleaved peptides were precipitated in anhydrous ether and purified by HPLC.

#### Preparation of Fmoc-hydrazine resins

1.5 g of 2-chlorotrityl chloride resins (resin loading: 1.14 mmol/g) were swelled in 12 mL DCM for 20 min with protective nitrogen and cooled in an ice bath. To the swollen 2-chlorotrityl chloride resins, Fmoc-hydrazine (4 equiv. relative to the resin loading) and DIEA (10 equiv.) dissolved in 15 mL DMF and 3 mL DCM was added slowly and dropwise. After 15 h reaction at room temperature, 0.69 mL methanol was added to quench the reaction and block unreacted sites. The resins were then washed successively with DMF, methanol and anhydrous ether. The washed resins were vacuum dried for 1 h. The prepared Fmoc-hydrazine resins were determined to have a loading of 0.346 mmol/g, which were used for the synthesis of peptide hydrazides.

#### Native chemical ligation

The general procedure for ligation of peptides using peptide hydrazides was followed as described previously^38^. Peptide hydrazide was dissolved in 100 mM phosphate buffer (pH 3.0) containing 6.0 M Gn·HCl to reach a concentration of 1.0 mM. Acetyl acetone (5 equiv. relative to the peptide hydrazide) and 4-mercaptophenylacetic acid (20 equiv.) were then added to the peptide mixture, which was allowed to react for 4 h on a shaker at 37 °C, followed by purification using HPLC to obtain the peptide thioester. The obtained peptide thioester was freeze-dried and dissolved in 100 mM phosphate buffer (pH 7.4) containing 50 mM TCEP. N-Terminal Cys-containing peptides (1.3 equiv. relative to the peptide thioester) were added to the mixture, and the cocktail was stirred for 2.0 h at 37 °C. After that, the ligated peptides were purified by HPLC. hd1–TEV–hd3-A and hd1–TEV–hd3-B were synthesized using this method.

### Oxidative dimerization of peptides

In a typical experiment, HPLC-purified peptide monomers with multiple Cys residues (100 μM) and multiple Pen residues (2 equiv.; 200 μM) were dissolved in phosphate buffers containing 50 μM selenol-l-cystine (SeCys). After reaction for ~24 h on a shaker at 37 °C, the formed products were characterized by HPLC and mass spectrometry. HPLC chromatograms and mass spectrometry were acquired using LabSolutions version 5.85 (Shimadzu) and flexControl version 3.4 (otofControl version 4.0.15.3248, trapControl Version 7.0; Bruker), respectively. Detailed conditions for the dimeric folding of peptides were provided in Supplementary Table 5.

### Circular dichroism measurements

#### General method

Circular dichroism (CD) spectra were measured using a 1.0 mm path length cuvette. All peptides were dissolved in water to reach a concentration of 50 μM. CD spectra were recorded in a wavelength range of 190‒260 nm with a bandwidth of 2.0 nm, a date pitch of 1.0 nm, a response time of 8.0 s, and a scanning speed of 50 nm/min. CD spectra were acquired using Spectra Manager version 1.52.01 (JASCO).

#### Thermal denaturation

The thermal denaturation CD spectra were recorded in a temperature range of 25‒95 °C with a heating rate of 5 °C/min. For temperature melts, the change of ellipticity at 220 nm was monitored as the temperature increased from 25 to 95 °C in an increment of 5 °C. Other experimental conditions were consistent with those of the general method.

#### Chemical denaturation

Peptide heterodimers with a concentration of 50 μM were dissolved in water containing 1, 3 and 6 M Gn·HCl, respectively. Then, CD spectra were recorded using the conditions given in the general method. The baseline signals from the aqueous Gn·HCl solution were subtracted from the corresponding spectrum.

### NMR characterization of peptide heterodimers

The hd1 and hd2 peptides were dissolved in H_2_O/D_2_O (90%/10%) and H_2_O/CD_3_CN (50%/50%) with a final concentration of 1.3 mM and 0.8 mM, respectively. All NMR experiments were conducted at 298 K on a Bruker AVANCE III 850 MHz spectrometer equipped with a 5 mm z-gradient ^1^H/^13^C/^15^N TCI cryogenic probe. Two-dimensional (2D) ^1^H–^1^H TOCSY (80 ms mixing time) and ^1^H–^13^C/^15^N HSQC spectra were measured for resonance assignments. 2D ^1^H–^1^H NOESY experiments with a mixing time of 200 or 300 ms were performed to extract ^1^H–^1^H distance. The phi and psi backbone torsion angles were predicted by TALOS+ using chemical shifts of HN, HA, CA, CB, and N^39^. The Xplor-NIH (version 2.53) program was used for the structure determination and refinement^40^. The intra- and inter-molecular hydrogen bond distance constraints inferred from the preliminarily calculated structures were applied in the late stage of structure determination. All NMR spectra were processed using TopSpin 3.5 and analyzed using NMRFAM-SPARKY^41^. ^1^H chemical shifts were referenced to DSS, and ^13^C/^15^N chemical shifts were referenced indirectly to DSS.

### Tryptic digestion analysis of disulfide connectivity

Freeze-dried peptide heterodimers (~100 μM) were dissolved in 100 mM phosphate buffer (pH = 6.0 or 7.4), and trypsin with a final concentration of 100 μg/mL was added to the reaction solution. The reaction mixture was kept in a shaker at 37 °C for 8 h (pH = 6.0) or 2 h (pH = 7.4). Then, the digested fragments were characterized using HPLC and mass spectrometry.

### Protein expression and purification

Genes encoding the protein sequences (Neo-2/15-hd1-B, MLK3-SH3-hd1-B, SUMO-hd3-B, and MLK3-SH3-hd1/hd3-B) were cloned into pET-28b(+) *E. coli* expression vectors (Supplementary Table 4). The recombinant plasmids were then transformed into chemically competent *E. coli* BL21 (DE3) cells. The cells were grown at 37 °C until *A*_600 nm_ reached 0.6–0.8 in fresh LB medium, and then the protein expression was induced by adding 0.5 mM isopropyl-β-d-thiogalactopyranoside (IPTG). After ~14 h culture at 16 °C, the cells were harvested and lysed by sonication on an ice bath using a lysis buffer (100 mM Tris, 500 mM NaCl, 1 mM PMSF, pH 7.9). The soluble fraction was clarified by centrifugation at 11,000 rpm for 30 min, and then purified by Ni^2+^ Sepharose column with a concentration gradient of imidazole. All proteins were finally purified using FPLC and HPLC, and identified by MALDI-TOF MS (flexControl version 3.4). The purified proteins were stored at ‒80 °C for the further labeling applications.

### Labeling of proteins

The purified proteins (5 or 12.5 μM) and the relevant Pen-bearing monomers modified with functional molecules (100 μM) were dissolved in buffers containing 50 μM selenol-l-cystine (SeCys). After reaction for 24 h on a shaker at 37 °C, the reaction mixture was analyzed by HPLC, SDS–PAGE, and MALDI-TOF MS. Detailed conditions for the labeling of proteins were provided in Supplementary Table 6. To further identify the protein labeling, the labeled proteins were freeze-dried and dissolved in 100 mM phosphate buffer (pH = 7.0), into which 2 µL AcTEV^TM^ Protease (10 U/µL in AcTEV^TM^ Protease buffer) was added. The reaction mixtures were kept on a shaker at 37 °C for 2 h. Then, the digested fragments were analyzed by HPLC (LabSolutions version 5.85) and MALDI-TOF MS (flexControl version 3.4).

### Statistics and reproducibility

No sample size calculation was performed. All experiments were repeated independently at least once with similar results. All results are reproducible.

### Reporting summary

Further information on research design is available in the Nature Research Reporting Summary linked to this article.

## Supplementary information

Supplementary Information

Reporting Summary

Description of additional supplementary files

Supplementary Data 1

Supplementary Software

## Data Availability

The NMR structure data generated in this study have been deposited in the PDB database under accession code 7FB8 and 7FBA. Any other data are available from corresponding authors upon reasonable request. Source data are provided with this paper.

## Source data

Source Data

## Code availability

The code used for the placement of disulfides into designed structures and instructions can be found at: https://github.com/atom-moyer/stapler. A representative script and associated parameter files used to design the peptides can be found in a Supplementary file. The Rosetta software suite is available for free to all non-commercial users and to commercial users for a fee: https://www.rosettacommons.org/.
