# Supplementary material for: De novo design and directed folding of disulfide-bridged peptide heterodimers
Source: Nat Commun. 2022 Mar 22;13:1539. doi: 10.1038/s41467-022-29210-x (PMC8941120; doi:10.1038/s41467-022-29210-x)
Supplement: Supplementary file 1 — Supplementary Information [file 41467_2022_29210_MOESM1_ESM.pdf]

Supplementary Information for

De Novo Design and Directed Folding of Disulfide-Bridged Peptide Heterodimers

Sicong Yao[1]†, Adam Moyer[2]†, Yiwu Zheng[1]†, Yang Shen,[1] Xiaoting Meng,[1] Chong Yuan,[3] Yibing Zhao,[1] Hongwei Yao[3]*, David Baker[2]*, and Chuanliu Wu[1]*

[1]Department of Chemistry, College of Chemistry and Chemical Engineering, The MOE Key Laboratory of Spectrochemical Analysis and Instrumentation, State Key Laboratory of Physical Chemistry of Solid Surfaces, Xiamen University, Xiamen, 361005, P.R. China. [2]Department of Biochemistry and Institute for Protein Design, University of Washington, Seattle, Washington 98195, United States. [3]Institute of Molecular Enzymology, School of Biology and Basic Medical Sciences, Soochow University, Suzhou 215123, P.R. China.

Correspondence to: chlwu@xmu.edu.cn; dabaker@uw.edu; hwyao@suda.edu.cn.

This PDF file includes:

Supplementary Figures S1 to S45
Supplementary Tables S1 to S6
References

Table of contents

1. Supplementary Data 3

1.1 Supplementary Tables 3

1.2 Interchain disulfide pairing chemistry 10

1.3 Designed peptide heterodimers 16

1.4 Orthogonality of peptide heterodimerizations 43

1.5 Potential applications of peptide heterodimerizations 55

2. References 67

1. Supplementary Data

1.1 Supplementary Tables

Supplementary Table S1. Sequence of peptides

Name	Sequence	m/z calcd. $[M+H]^+$
Peptide 1	Ac-WGCGKGGGCG-NH_2	922.3658
Peptide 2	Ac-WGPenGKGGGPenG-NH_2	978.4284
Peptide 3	Ac-WGCGGKGGCGKGGGPenGKGGGCGW-NH_2	2055.8591
Peptide 4	Ac-WGCGGKGGCGKGGGCGKGGGCGW-NH_2	2027.8278
hd1-A	EPenTGHIEGPTPenTPenHCK-NH_2	1801.7852
hd1-B	DDKDCDEYCKKTKE-NH_2	1718.7360
hd2-A	ECREYGPPenKPenPenA-NH_2	1444.6204
hd2-B	APenCECGPTRECK-NH_2	1326.5421
hd3-A	AAEAPenERTWAEPenAEKPenKRTHEEPenARKA-NH_2	3192.5357
hd3-B	AAEACDRTGAECEEKCDRTGEECRRKA-NH_2	2987.3009
hd4-A	EAPenDRHGEAPenDRAA-NH_2	1558.6849
hd4-B	AAEACRRDHEACAR-NH_2	1557.7121
hd5-A	DKAAAPenEKWDAEPenKKERKEPenAEALS-NH_2	2896.4190
hd5-B	DKARACEEHADACQKETTECAKALS-NH_2	2708.2260
hd1-A-Cys	ECTGHIEGPTCTCHCK-NH_2	1717.6913
hd2-A-Cys	ECREYGPCKCCA-NH_2	1360.5265
hd2-B-Cys	ACCECGPTRECK-NH_2	1298.5108
hd3-A-Cys	AAEACERTWAECAEKCKRTHEECARKA-NH_2	3080.4104
hd1-TEV-hd3-A	EPenTGHIEGPTPenTPenHCKENLYFQGAAEAPenERTWAEPenAEKPenKRTHEEPenARKA-NH_2	5829.6722
hd1-TEV-hd3-B	DDKDCDEYCKKTKEGSENLYFQGGSAAEACDRTGAECEEKCDRTGEECRRKA-NH_2	5829.4955
FITC hd1-A	FITC-βAEPenTGHIEGPTPenTPenHCK-NH_2	2262.8609
FITC hd3-A	FITC-βAAAEAPenERTWAEPenAEKPenKRTHEEPenARKA-NH_2	3653.6103
Biotin hd1-A	Biotin-GGEPenTGHIEGPTPenTPenHCK-NH_2	2142.9085

A: Pen-bearing monomer;

B: Cys-bearing monomer;

The green Cys residues denote the ligation site for native chemical ligation;
FITC: fluorescein isothiocyanate isomer I;
βA: β-alanine.

Supplementary Table S2. NMR and refinement statistics for the 20 lowest-energy structures of hd1

	**Protein**
**NMR distance and dihedral constraints**	
Distance constraints	
Total NOE	703
Intra-residue	245
Inter-residue	458
Sequential ($\|i-j\| = 1$)	203
Medium-range ($\|i-j\| \leq 4$)	128
Long-range ($\|i-j\| \geq 5$)	44
Intermolecular	83
Hydrogen bonds	15
Total dihedral angle restraints	33
ϕ	17
ψ	16
**Structure statistics**	
Violations (mean and s.d.)	
Distance constraints (Å)	0.018±0.001
Dihedral angle constraints (º)	0.290±0.061
Max. dihedral angle violation (º)	2.312
Max. distance constraint violation (Å)	0.147
Deviations from idealized geometry	
Bond lengths (Å)	0.002±0.000
Bond angles (º)	0.471±0.007
Impropers (º)	0.363±0.010
Average pairwise r.m.s. deviation* (Å)	
Heavy	0.57±0.05
Backbone	0.16±0.03

*Pairwise r.m.s. deviation was calculated among 20 refined structures.

Supplementary Table S3. NMR and refinement statistics for the 20 lowest-energy structures of hd2

	**Protein**
**NMR distance and dihedral constraints**	
Distance constraints	
Total NOE	530
Intra-residue	194
Inter-residue	336
Sequential ($\|i-j\| = 1$)	155
Medium-range ($\|i-j\| \leq 4$)	47
Long-range ($\|i-j\| \geq 5$)	83
Intermolecular	51
Hydrogen bonds	14
Total dihedral angle restraints	22
ϕ	12
ψ	10
**Structure statistics**	
Violations (mean and s.d.)	
Distance constraints (Å)	0.019±0.004
Dihedral angle constraints (º)	0.049±0.089
Max. dihedral angle violation (º)	< 2
Max. distance constraint violation (Å)	0.351
Deviations from idealized geometry	
Bond lengths (Å)	0.002±0.000
Bond angles (º)	0.503±0.020
Impropers (º)	0.369±0.023
Average pairwise r.m.s. deviation* (Å)	
Heavy	0.96±0.16
Backbone	0.33±0.06

*Pairwise r.m.s. deviation was calculated among 20 refined structures.

Supplementary Table S4. Sequence of proteins

Name	Sequence	m/z calcd. (oxidized)
Neo-2/15-hd1-B	GSSHHHHHHSSGLVPRGSHMPKKKIQLHAEHALYDALMILNIVKTNSPPAEEKLEDYAFNFELILEEIARLFESGDQKDEAEKAKRMKEWMKRIKTTASEDEQEEMANAIITILQSWIFSGSGSGSGENLYFQ*GDDKDCDEYCKKTKE	16888.247 $[M+H]^{+}$
MLK3-SH3-hd1-B	GSSHHHHHHSSGLVPRGSHMHMPVWTALFDYEPSGQDELALRKGDRVEVLSRDAAISGDEGWWAGQVGGQVGIFPSNYVSRGGSGSGSGENLYFQ*GDDKDCDEYCKKTKE	11984.553 $[M+H]^{+}$
SUMO-hd3-B	MNWSHPQFEKSSGSSGGHHHHHHGGSGGSGSDSEVNQEAKPEVKPEVKPETHINLKVSDGSSEIFFKIKKTTPLRRLMEAFAKRQGKEMDSLRFLYDGIRIQADQAPEDLDMEDNDIIEAHREQIGGLAAEACDRTGAECEEKCDRTGEECRRKA	8634.620 $[M+2H]^{2+}$
MLK3-SH3-hd1/hd3-B	GSHMAAEACDRTGAECEEKCDRTGEECRRKAENLYFQ*GGSGSGSGHMPVWTALFDYEPSGQDELALRKGDRVEVLSRDAAISGDEGWWAGQVGGQVGIFPSNYVSRGGSGSGSGENLYFQ*GDDKDCDEYCKKTKE	14540.520 $[M+H]^{+}$

* indicates the cleavage site of AcTEV™ Protease.

Supplementary Table S5. Reaction conditions for the dimeric folding of peptides

Reaction	Pen-bearing monomer	Cys-bearing monomer	SeCys	pH
Dimerization of hd1	200 μM	100 μM	50 μM	10.0
Dimerization of hd2	200 μM	100 μM	50 μM	7.4
Dimerization of hd3	200 μM	100 μM	50 μM	10.0
Dimerization of hd4	200 μM	100 μM	50 μM	8.5
Dimerization of hd5	120 μM	100 μM	50 μM	10.0
Orthogonal reaction between hd1 and hd3	100 μM	50 μM	50 μM	10.0
Orthogonal reaction between hd1 and hd4	100 μM	50 μM	50 μM	8.5
Orthogonal reaction between hd2 and hd3	100 μM	50 μM	50 μM	8.5
Orthogonal reaction between hd2 and hd4	100 μM	50 μM	50 μM	8.5
Orthogonal reaction between hd3 and hd4	100 μM	50 μM	50 μM	8.5
Orthogonal reaction between hd1 and hd2	100 μM	50 μM	50 μM	7.4
Orthogonal reaction between hd1, hd3 and hd4	100 μM	50 μM	75 μM	8.5
Orthogonal reaction between hd2, hd3 and hd4	100 μM	50 μM	75 μM	8.5
Dimerization of heterotrimer1	100 μM	300 μM[a], 150 μM[b]	100 μM	10.0
Dimerization of heterotrimer2	500 μM[a], 200 μM[b]	100 μM	175 μM	10.0

a: Pen-bearing monomer or Cys-bearing monomer of hd1;

b: Pen-bearing monomer or Cys-bearing monomer of hd3.

Supplementary Table S6. Reaction conditions for labeling of proteins with functional molecules

Reaction	Pen-bearing monomer	Protein	SeCys	pH
Labeling of Neo-2/15	100 μM[a]	12.5 μM	50 μM	10.0
Labeling of MLK3-SH3	100 μM[a]	5 μM	50 μM	10.0
Labeling of SUMO	45 μM[b]	5 μM	22.5 μM	10.0
Orthogonal labeling of Neo-2/15 and SUMO	100 μM[a], 45 μM[b]	12.5 μM[d], 5 μM[f]	72.5 μM	10.0
Orthogonal labeling of MLK3-SH3 and SUMO	100 μM[a], 45 μM[b]	5 μM[e], 5 μM[f]	72.5 μM	10.0
Double labeling of MLK3-SH3	100 μM[c], 45 μM[b]	5 μM	72.5 μM	10.0

a: Pen-bearing monomer of hd1 conjugated with a fluorescein;

b: Pen-bearing monomer of hd3 conjugated with a fluorescein;

c: Pen-bearing monomer of hd1 conjugated with a biotin;

d: Neo-2/15; e: MLK3-SH3; f: SUMO.

1.2 Interchain disulfide pairing chemistry

a)

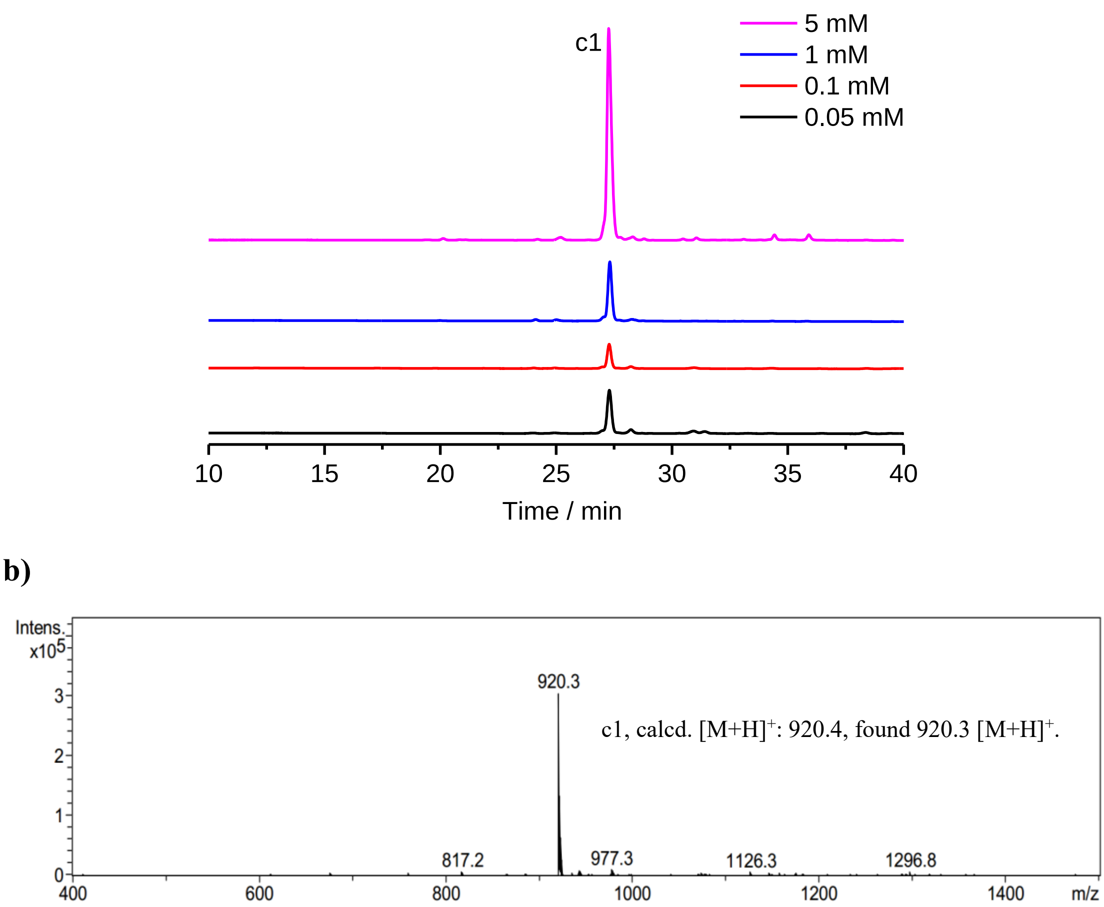

Supplementary Figure S1. Oxidation of peptide 1. a) Chromatograms showing the oxidation of peptide 1 with different concentrations. Reaction conditions: peptide 1, 100 mM phosphate buffer (pH 7.4) containing 50% DMSO, at 37 °C for 24 hours. **b)** Mass spectrum of the product formed after the oxidation of peptide 1.

a)

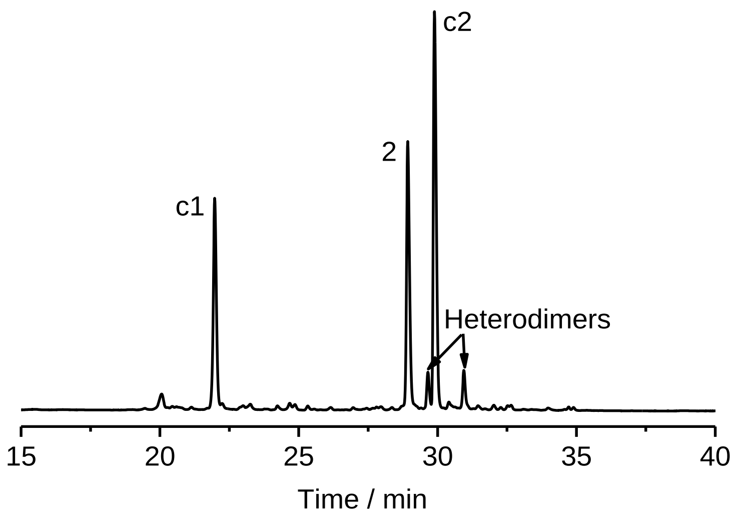
c2
2
c1
Heterodimers
15
20
25
30
35
40
Time / min

b)

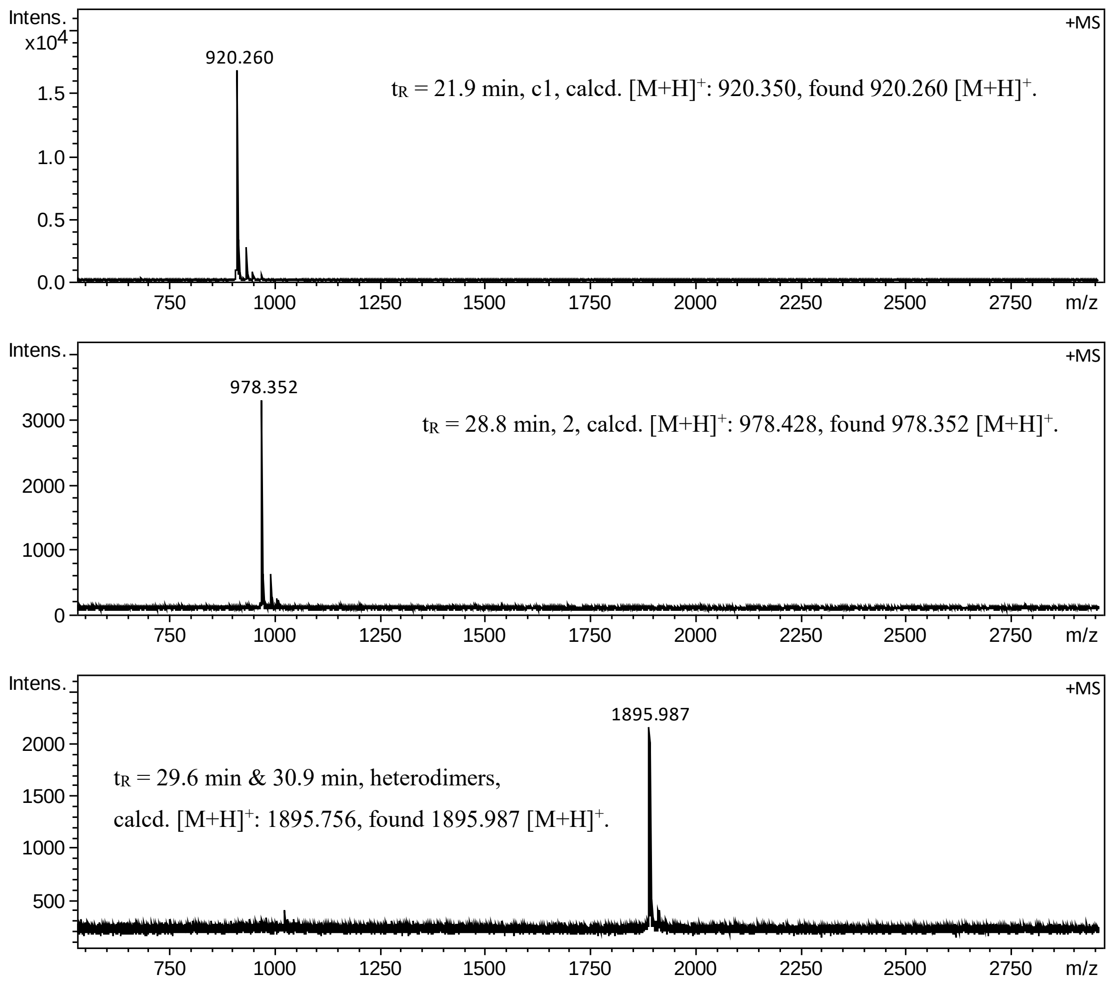
Intens.
x10^4
920.260
+MS
tR = 21.9 min, c1, calcd. [M+H]+: 920.350, found 920.260 [M+H]+.
978.352
tR = 28.8 min, 2, calcd. [M+H]+: 978.428, found 978.352 [M+H]+.
1895.987
tR = 29.6 min & 30.9 min, heterodimers,
calcd. [M+H]+: 1895.756, found 1895.987 [M+H]+.
m/z

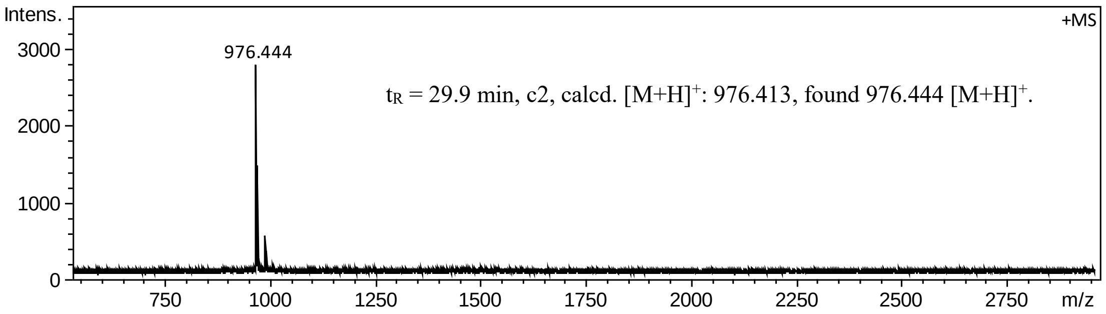

Supplementary Figure S2. Oxidation of peptides 1 and 2. a) Chromatogram showing the oxidation of peptides 1 and 2. Reaction conditions: 0.1 mM peptide 1, 0.2 mM peptide 2, 100 mM phosphate buffer (pH 7.4) containing 50% DMSO, at 37 °C for 24 hours. **b)** Mass spectra of the products formed after the oxidation of peptides 1 and 2.

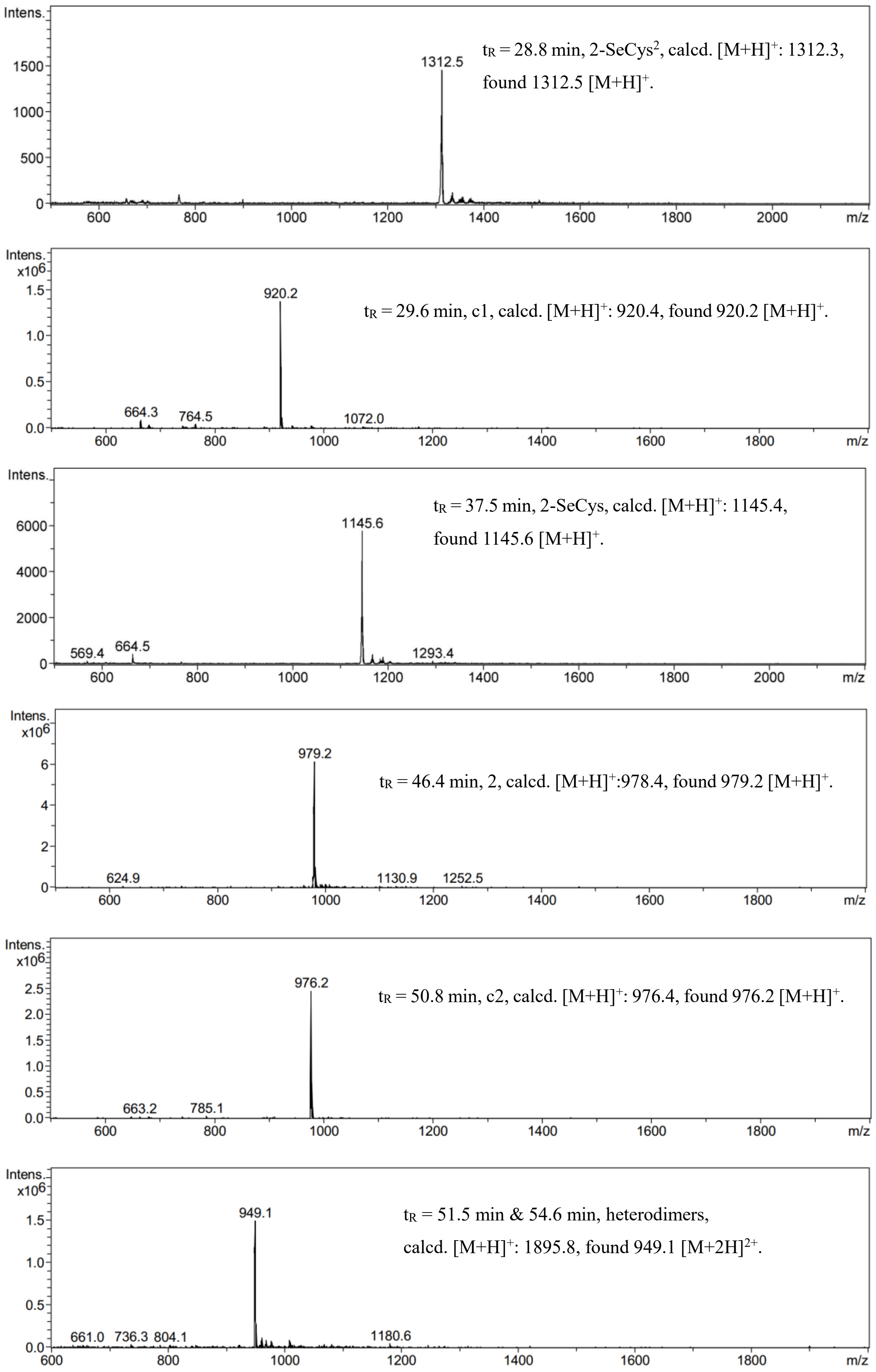

Supplementary Figure S3. Mass spectra of the products formed after the oxidation of peptides 1 and 2 in the presence of SeCys.

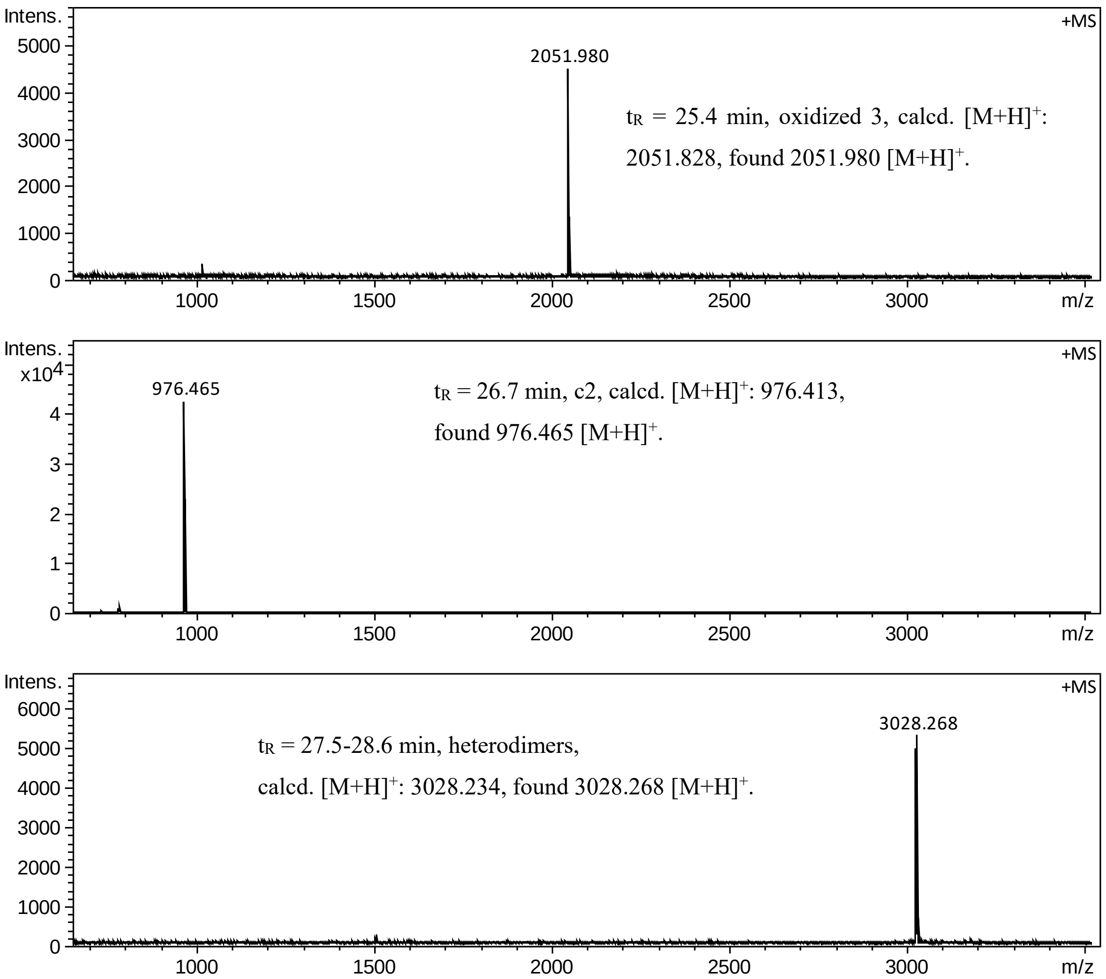

Supplementary Figure S4. Mass spectra of the products formed after the oxidation of peptides 2 and 3 in the presence of SeCys.

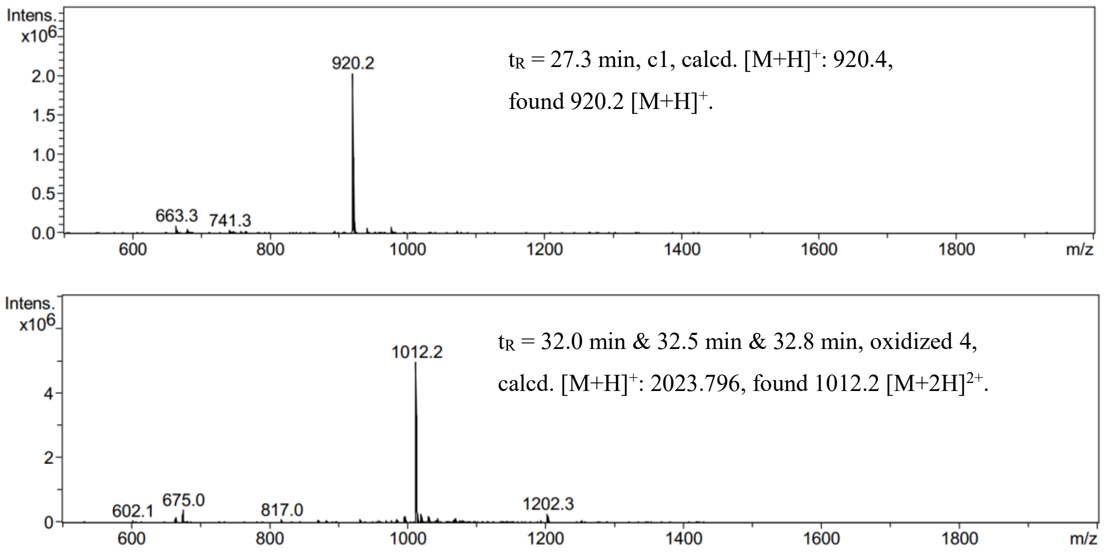

Supplementary Figure S5. Mass spectra of the products formed after the oxidation of peptides 1 and 4 in the presence of SeCys.

1.3 Designed peptide heterodimers

a)

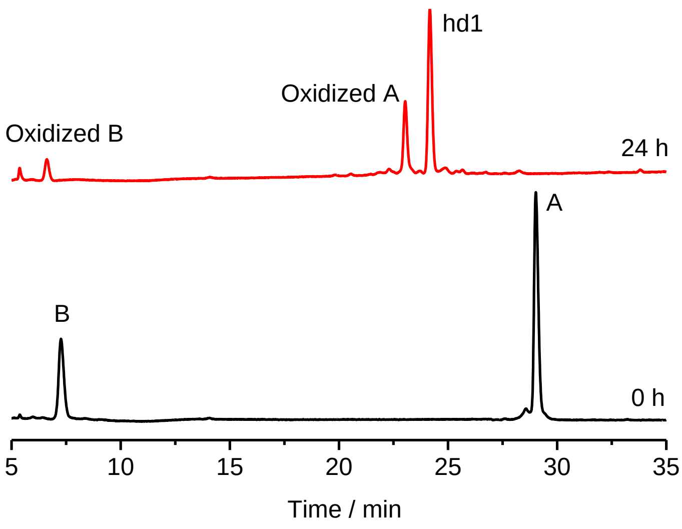

b)

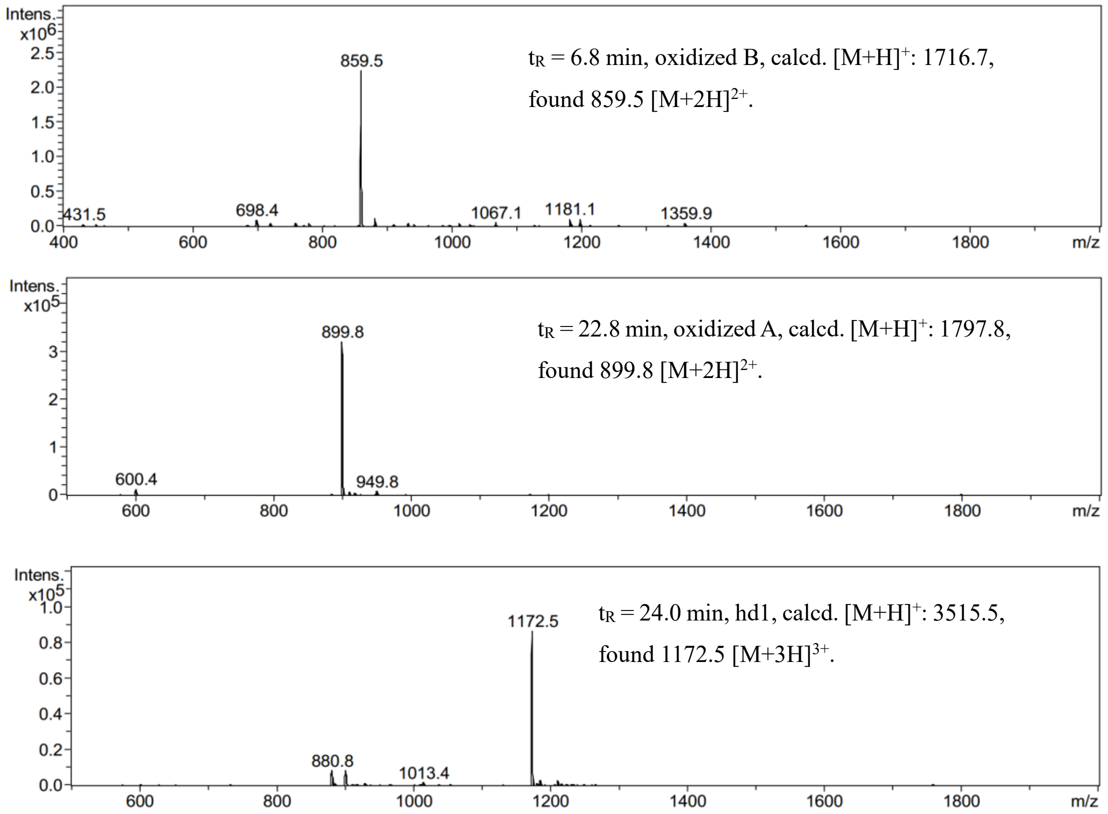

Supplementary Figure S6. Dimerization of hd1. a) Chromatograms showing the dimerization of hd1. A: Pen-bearing monomer, B: Cys-bearing monomer. Reaction conditions: 200 µM A, 100 µM B, 50 µM SeCys, 100 mM Gly-NaOH buffer (pH 10.0), at 37 °C for 24 hours. **b)** Mass spectra of the products formed after the dimerization of hd1.

a)

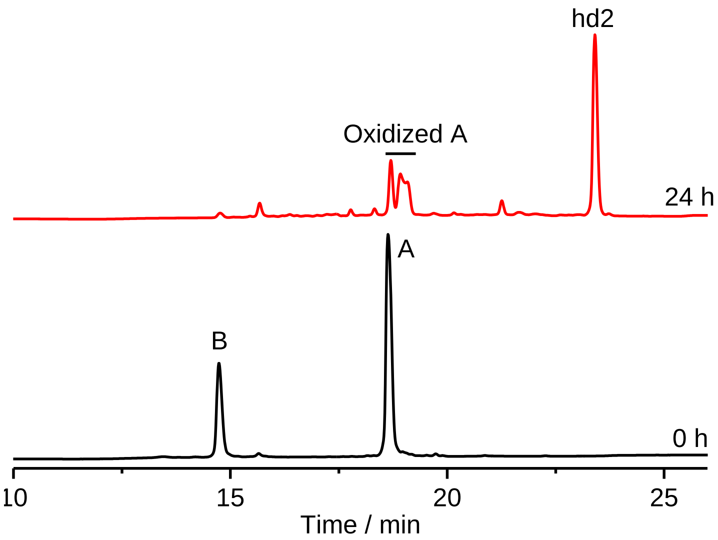

b)

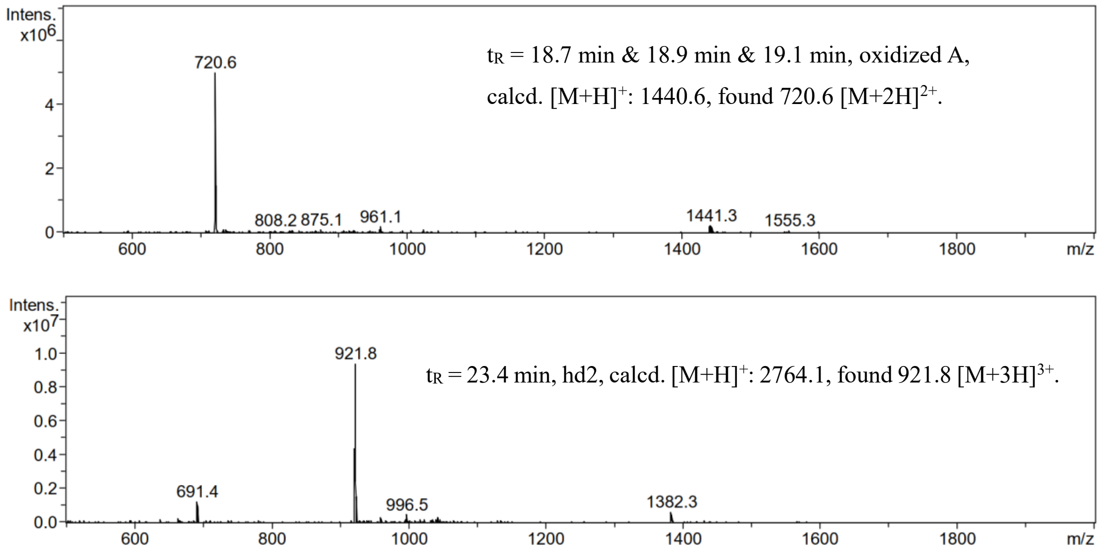

Supplementary Figure S7. Dimerization of hd2. a) Chromatograms showing the dimerization of hd2. A: Pen-bearing monomer, B: Cys-bearing monomer. Reaction conditions: 200 µM A, 100 µM B, 50 µM SeCys, 100 mM phosphate buffer (pH 7.4), at 37 °C for 24 hours. **b)** Mass spectra of the products formed after the dimerization of hd2.

a)

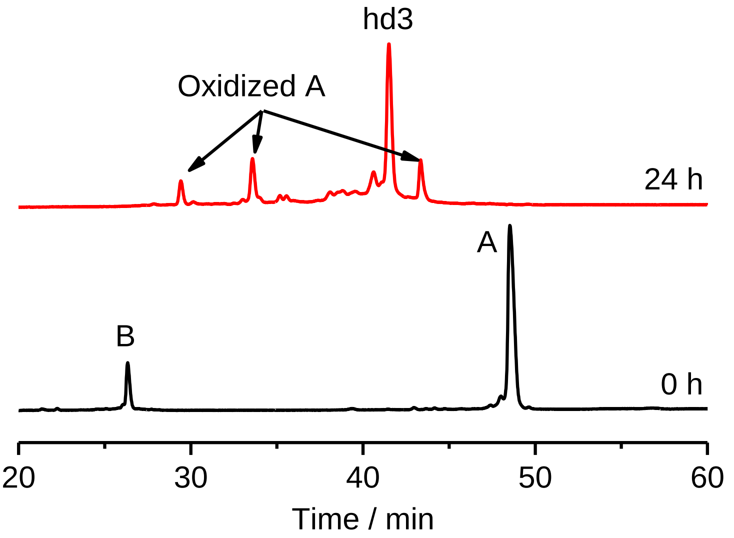

b)

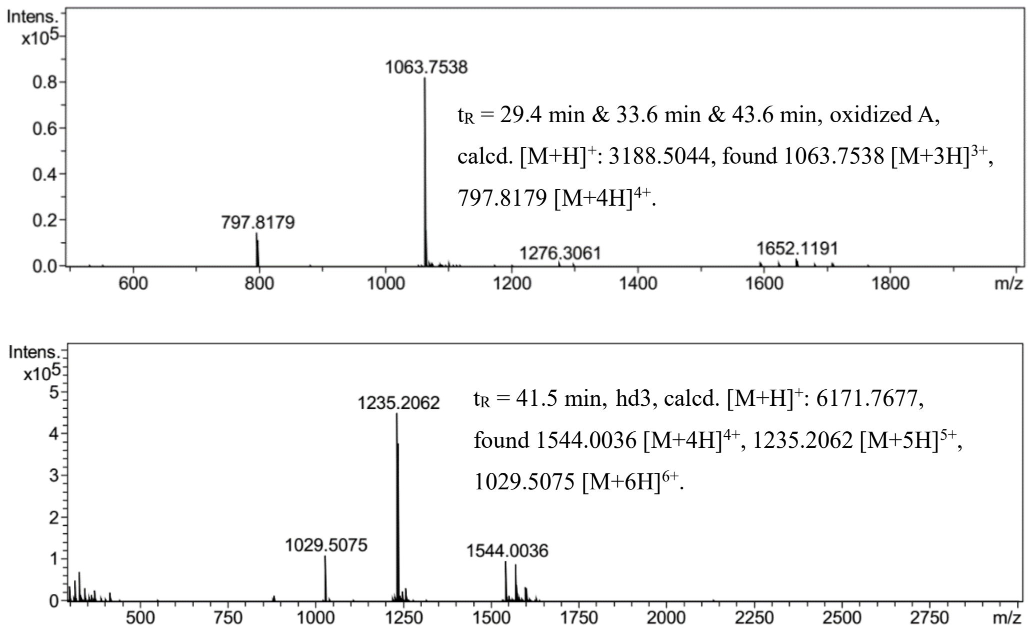

Supplementary Figure S8. Dimerization of hd3. a) Chromatograms showing the dimerization of hd3. A: Pen-bearing monomer, B: Cys-bearing monomer. Reaction conditions: 200 µM A, 100 µM B, 50 µM SeCys, 100 mM Gly-NaOH buffer (pH 10.0), at 37 °C for 24 hours. **b)** Mass spectra of the products formed after the dimerization of hd3.

a)

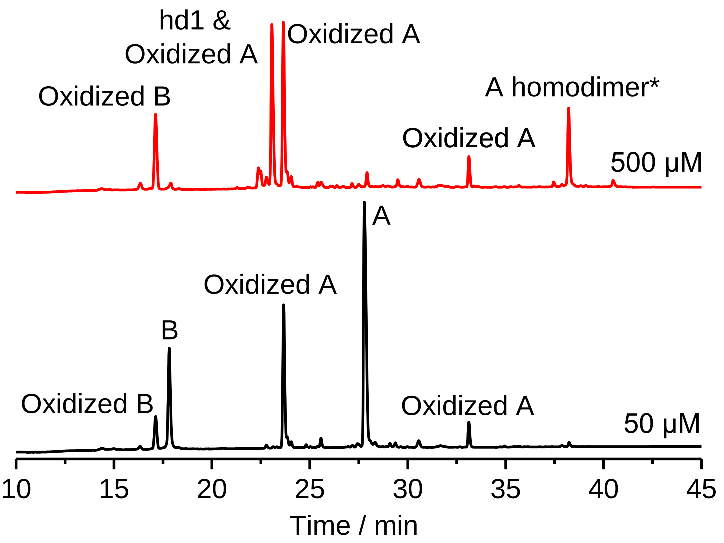

b)

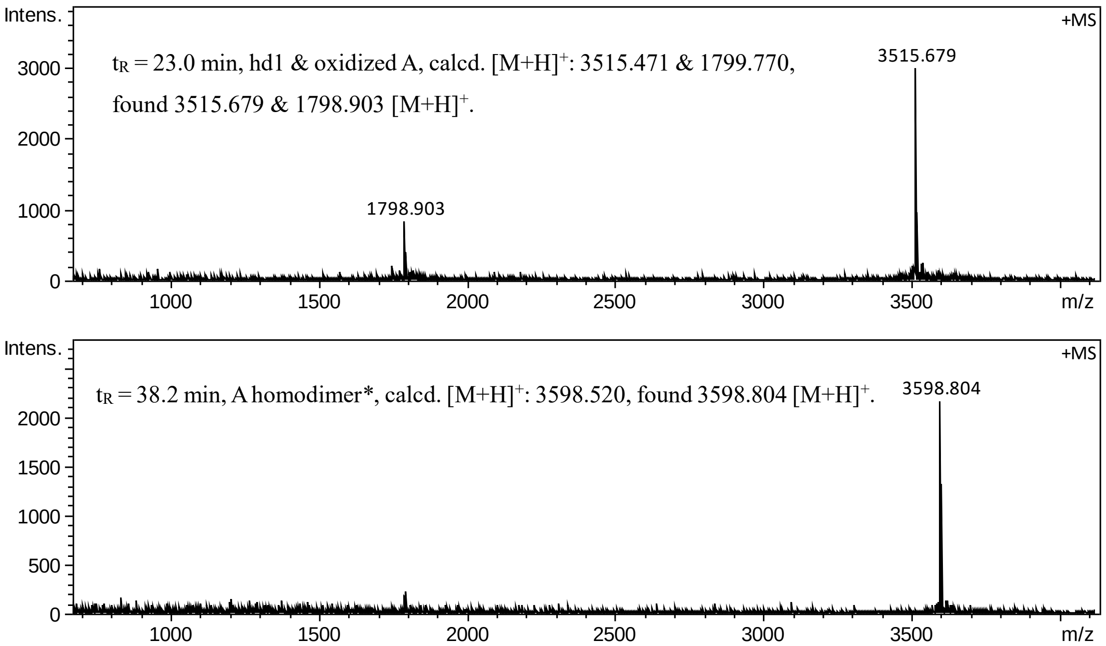

Supplementary Figure S9. Oxidation of hd1 in the absence of SeCys. a) Chromatograms showing the oxidation of hd1 with GSSG in different concentrations. A: Pen-bearing monomer, B: Cys-bearing monomer. Reaction conditions: 200 µM A, 100 µM B, 50 µM or 500 µM GSSG, 100 mM Gly-NaOH buffer (pH 10.0), at 37 °C for 24 hours. * denotes A homodimer containing three disulfides. **b)** Mass spectra of the products formed after the oxidation.

a)

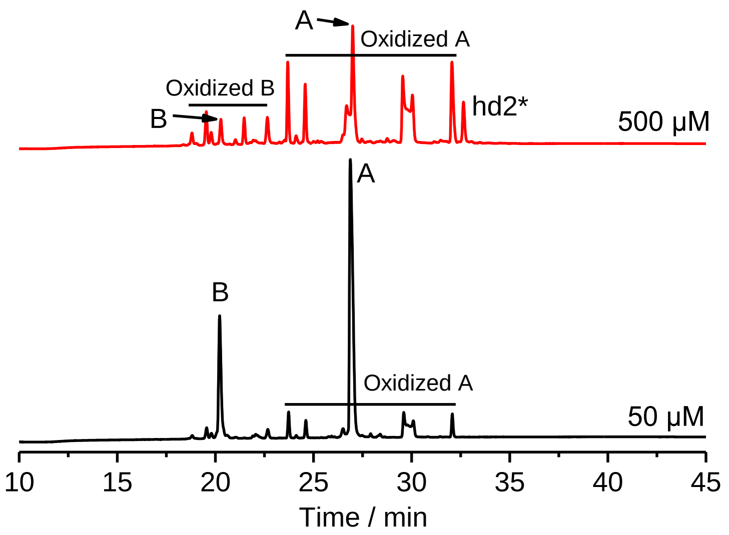

b)

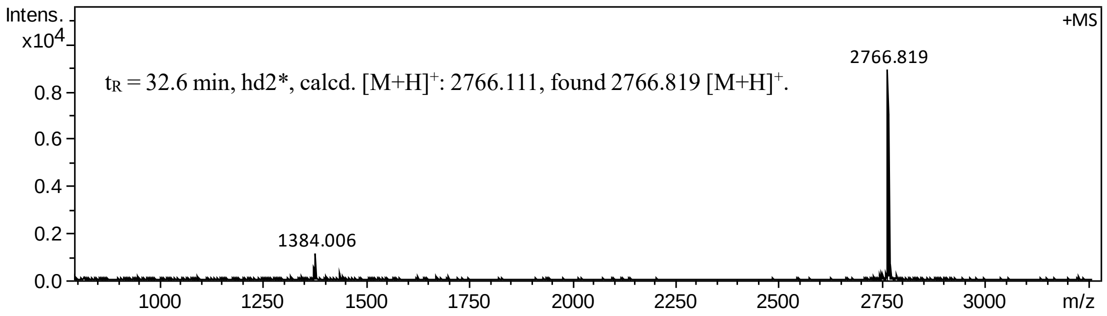

Supplementary Figure S10. Oxidation of hd2 in the absence of SeCys. a) Chromatograms showing the oxidation of hd2 with GSSG in different concentrations. A: Pen-bearing monomer, B: Cys-bearing monomer. Reaction conditions: 200 μM A, 100 μM B, 50 μM or 500 μM GSSG, 100 mM phosphate buffer (pH 7.4), at 37 °C for 24 hours. hd2* are a hd2 heterodimer containing three disulfides. **b)** Mass spectrum of the product hd2* formed after the oxidation.

a)

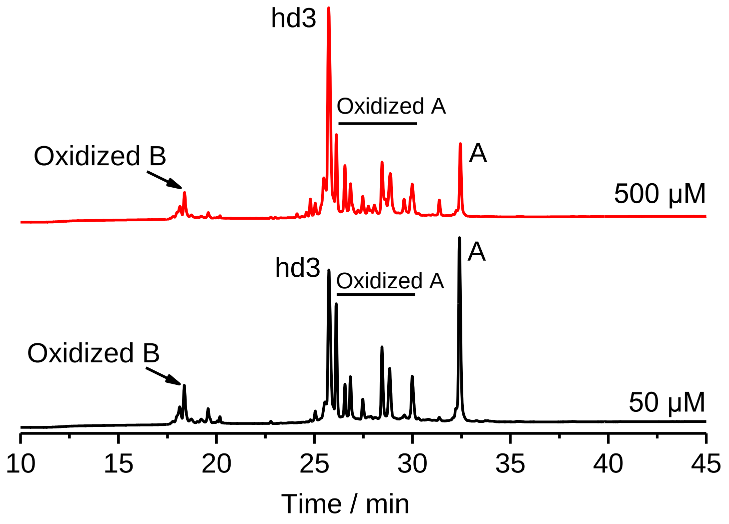

b)

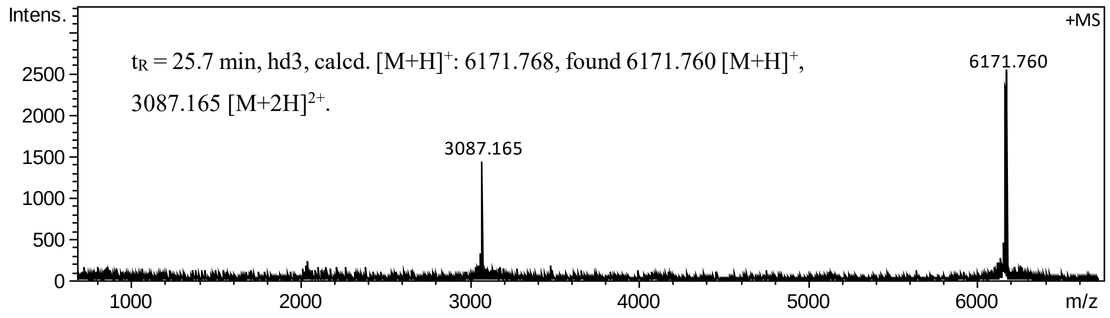

Supplementary Figure S11. Oxidation of hd3 in the absence of SeCys. a) Chromatograms showing the oxidation of hd3 with GSSG in different concentrations. A: Pen-bearing monomer, B: Cys-bearing monomer. Reaction conditions: 200 µM A, 100 µM B, 50 µM or 500 µM GSSG, 100 mM Gly-NaOH buffer (pH 10.0), at 37 °C for 24 hours. **b)** Mass spectrum of the product hd3 formed after the oxidation.

a)

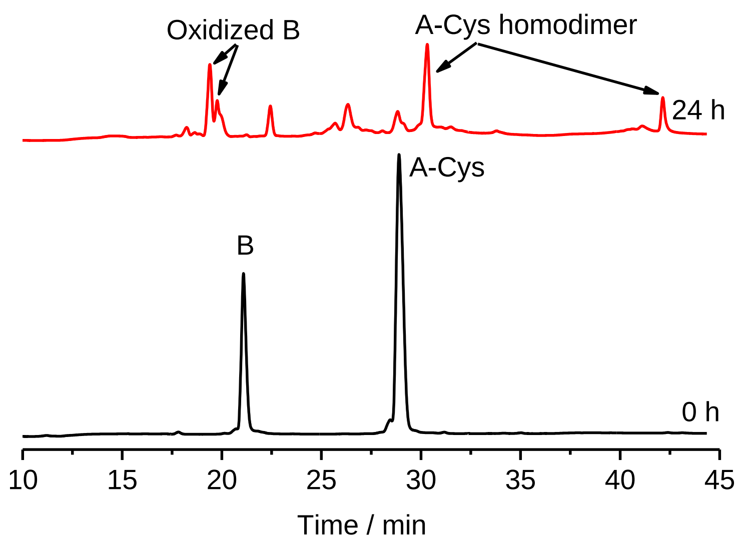

b)

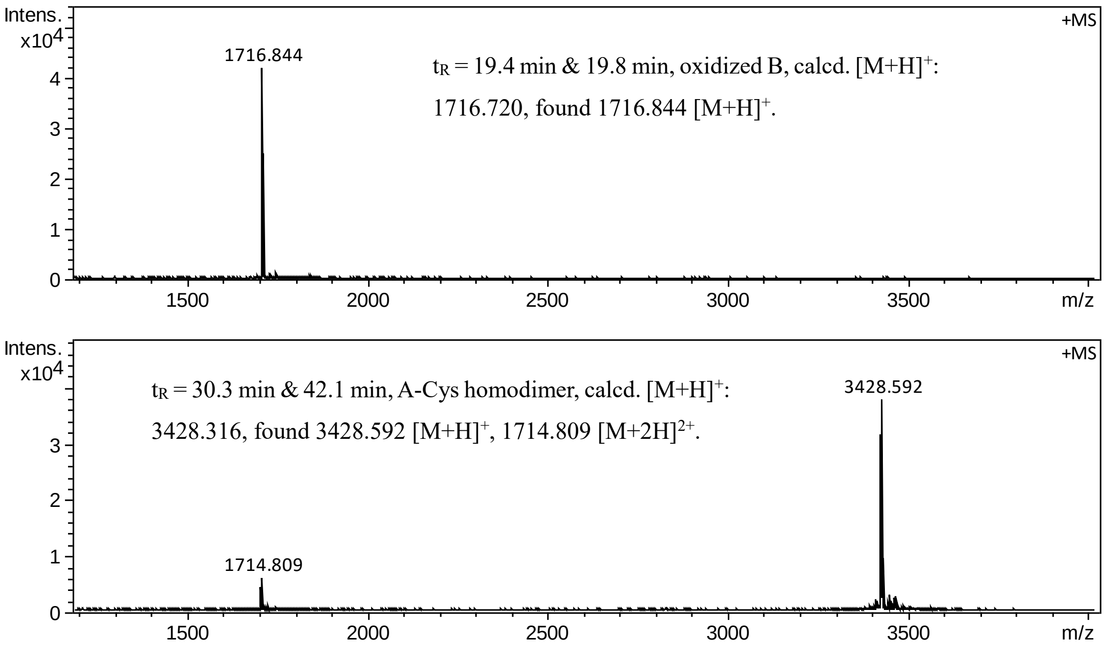

Supplementary Figure S12. Oxidation of control peptides for hd1 (no heterodimer was detected by HPLC). a) Chromatograms showing the oxidation of control peptides for hd1. A-Cys: Pen-bearing monomer of hd1 in which all Pen residues were replaced with Cys residues, B: Cys-bearing monomer of hd1. Reaction conditions: 200 μM A-Cys, 100 μM B, 50 μM SeCys, 100 mM Gly-NaOH buffer (pH 10.0), at 37 °C for 24 hours. **b)** Mass spectra of the products formed after the oxidation.

a)

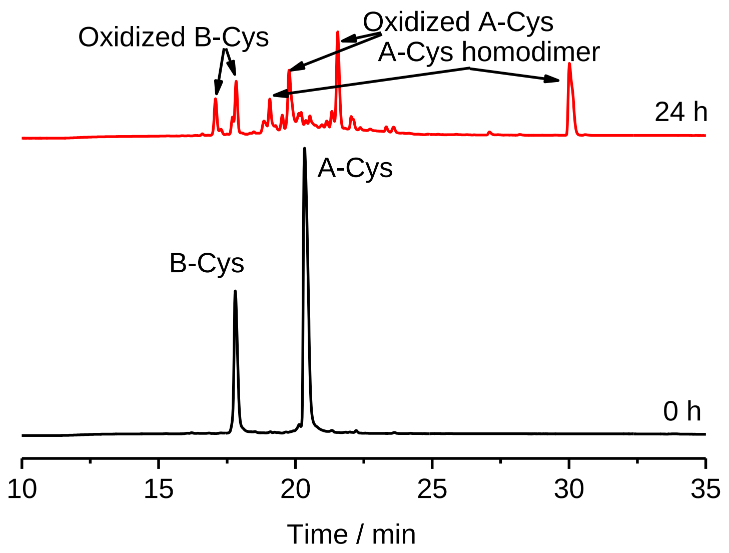

b)

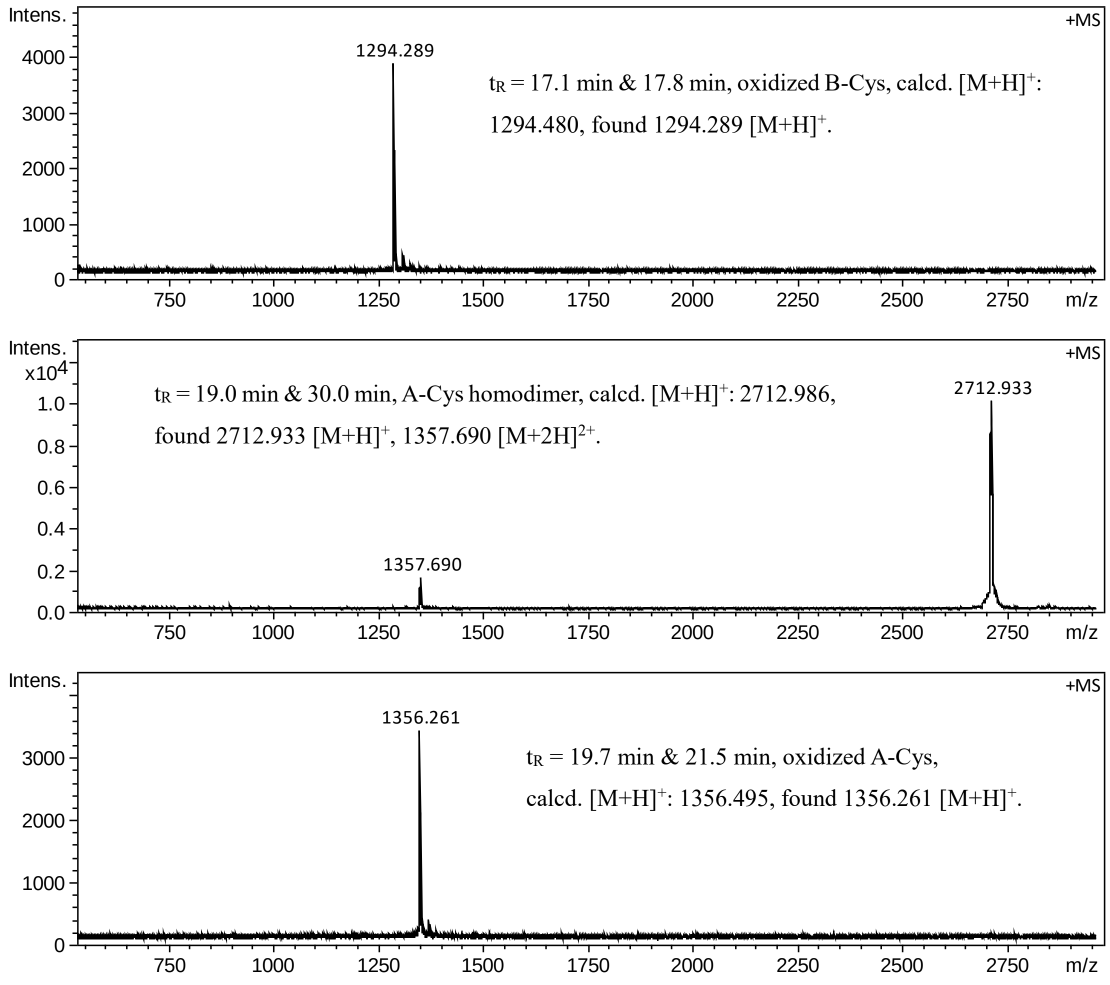

Supplementary Figure S13. Oxidation of control peptides for hd2 (no heterodimer was detected by HPLC). a) Chromatograms showing the oxidation of control peptides for hd2. A-Cys and B-Cys: Pen-bearing monomers of hd2 in which all Pen residues were replaced with Cys residues. Reaction conditions: 200 μM A-Cys, 100 μM B-Cys,

50 μM SeCys, 100 mM phosphate buffer (pH 7.4), at 37 °C for 24 hours. **b)** Mass spectra of the products formed after the oxidation.

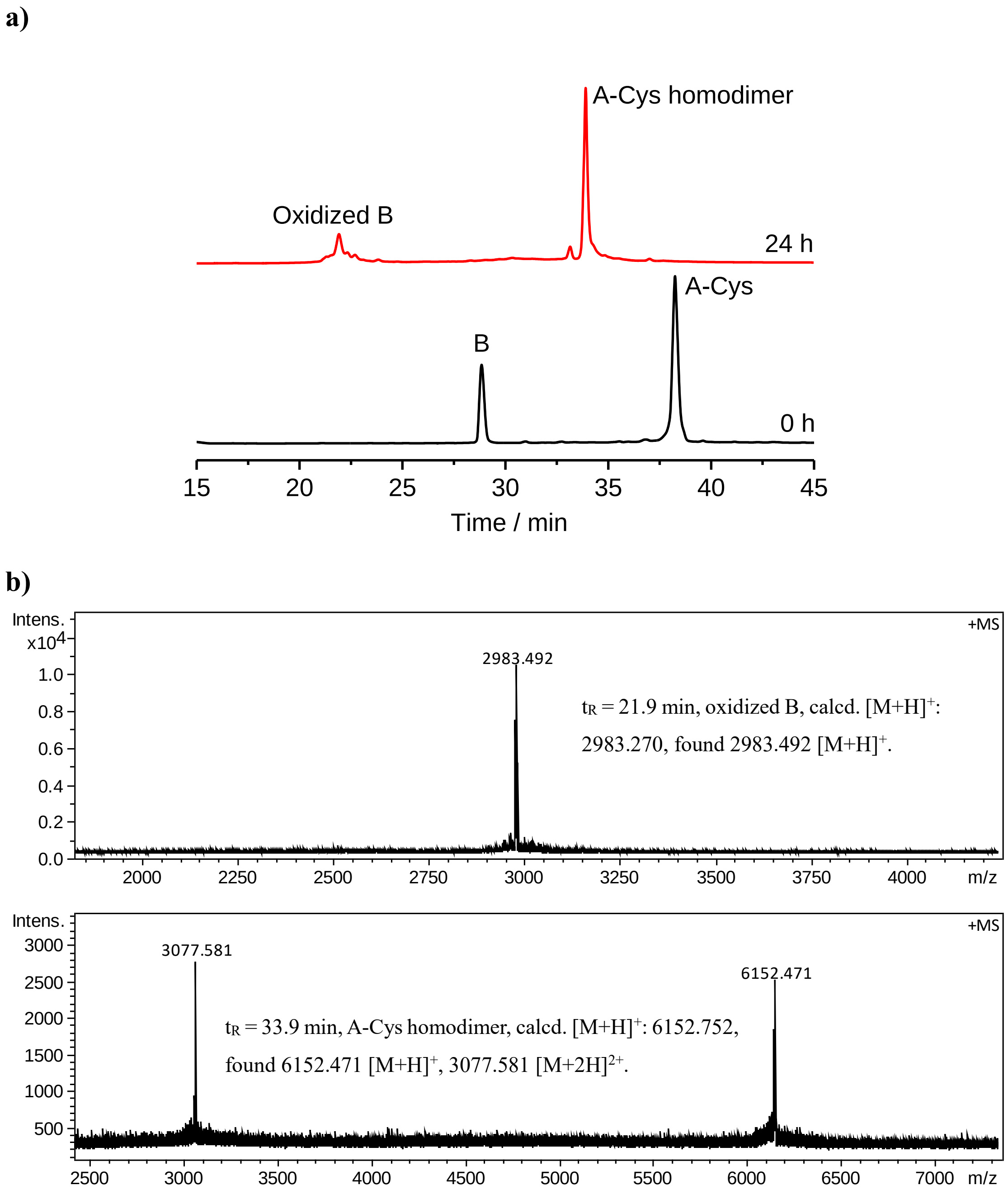

Supplementary Figure S14. Oxidation of control peptides for hd3 (no heterodimer was detected by HPLC). a) Chromatograms showing the oxidation of control peptides for hd3. A-Cys: Pen-bearing monomer of hd3 in which all Pen residues were replaced with Cys residues, B: Cys-bearing monomer of hd3. Reaction conditions: 200 µM A-Cys, 100 µM B, 50 µM SeCys, 100 mM Gly-NaOH buffer (pH 10.0), at 37 °C for 24 hours. **b)** Mass spectra of the products formed after the oxidation.

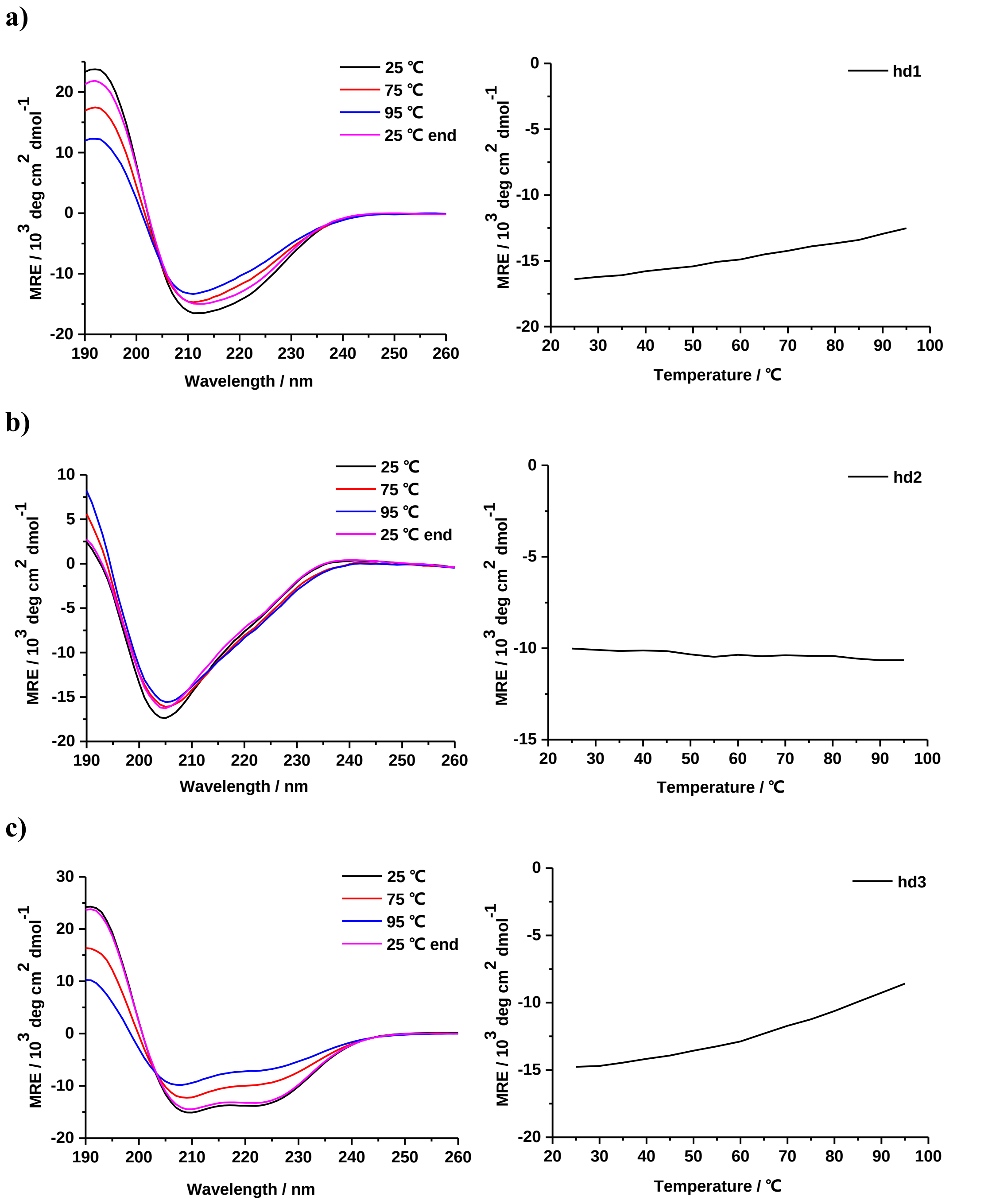

Supplementary Figure S15. CD spectra showing thermal denaturation of hd1 (a), hd2 (b) and hd3 (c), respectively. Left, wavelength scan at 25 °C, 75 °C, 95 °C and after cooling to 25 °C. Right, CD temperature melts, monitoring absorption at 220 nm as temperature was increased from 25 to 95 °C.

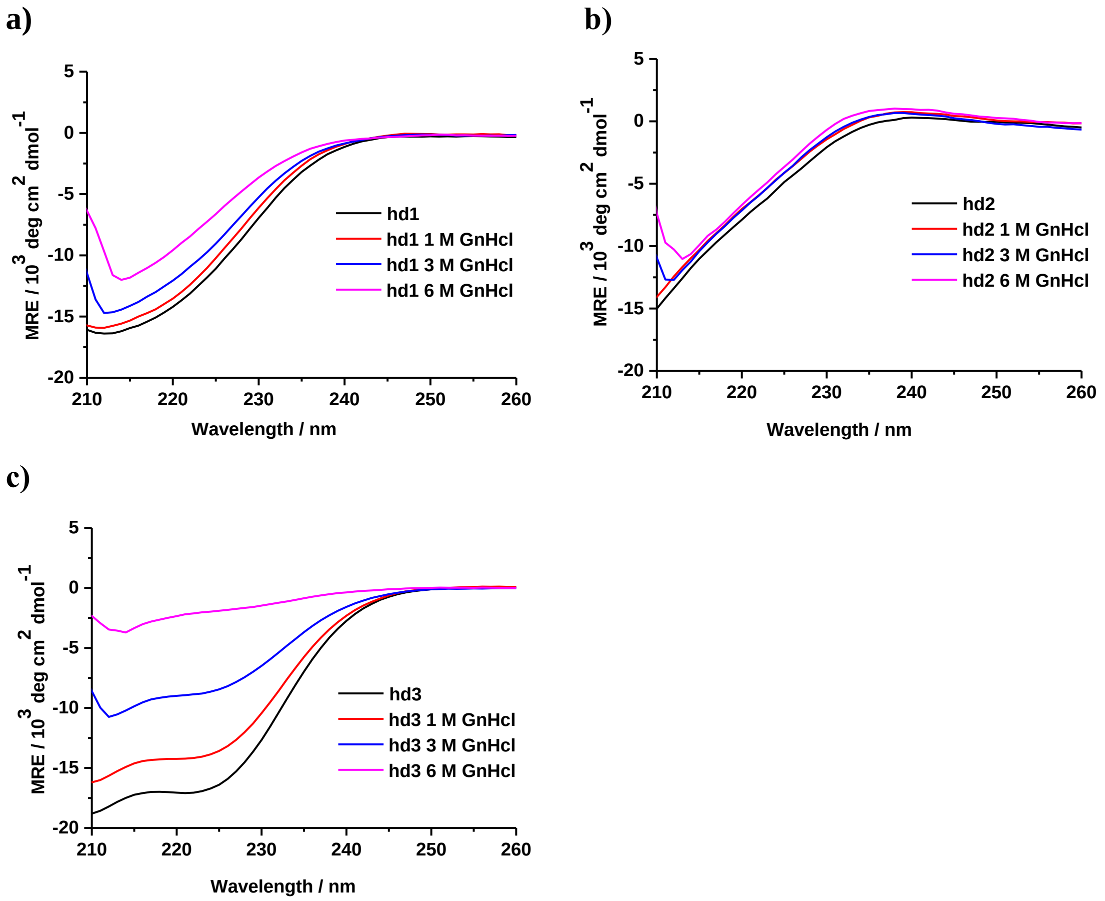

Supplementary Figure S16. CD spectra showing chemical denaturation of hd1 (a), hd2 (b) and hd3 (c), respectively. Gn HCl denaturation of the heterodimers measured by CD monitoring absorption with increasing concentration of denaturant.

a)

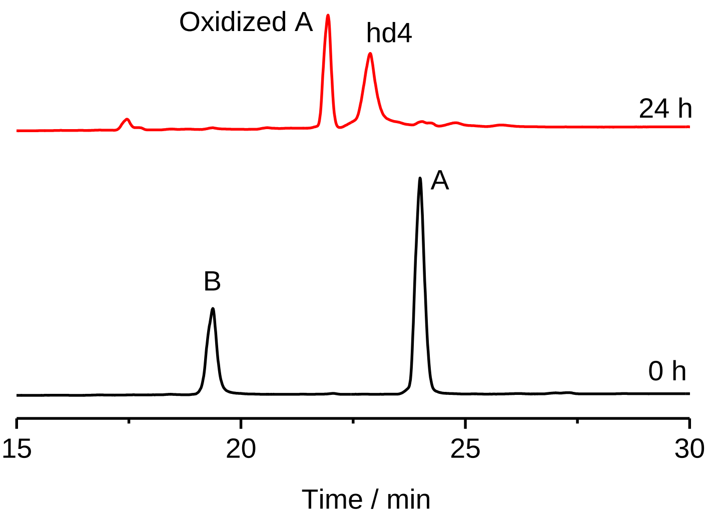

b)

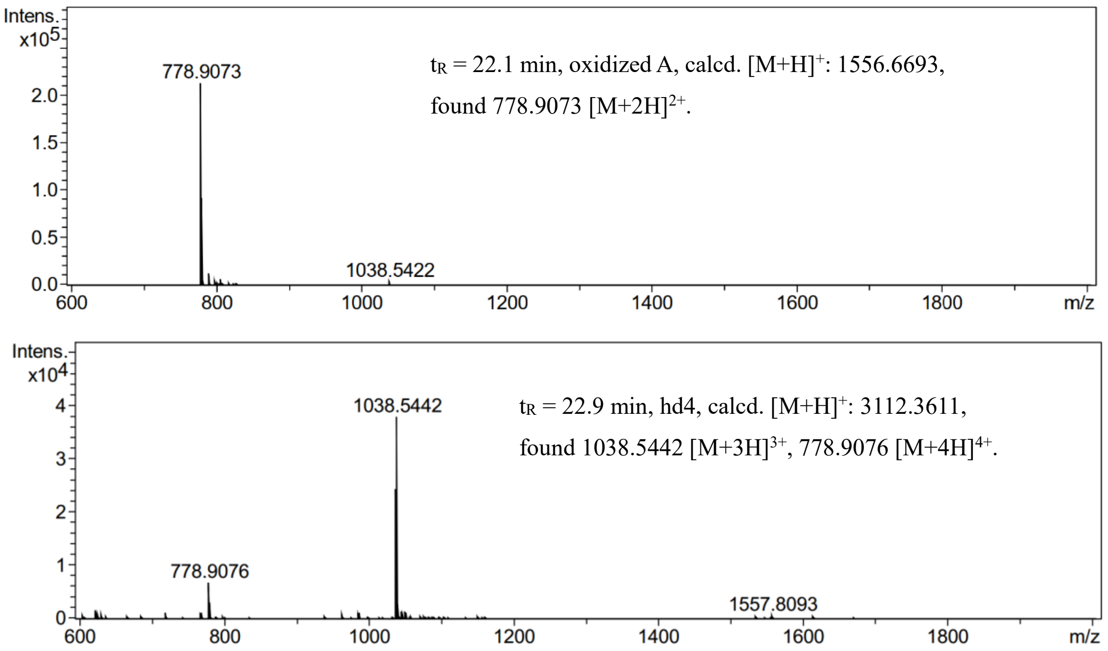

Supplementary Figure S17. Dimerization of hd4. a) Chromatograms showing the dimerization of hd4. A: Pen-bearing monomer, B: Cys-bearing monomer. Reaction conditions: 200 µM A, 100 µM B, 50 µM SeCys, 50 mM Tris buffer (pH 8.5), at 37 °C for 24 hours. **b)** Mass spectra of the products formed after the dimerization of hd4.

a)

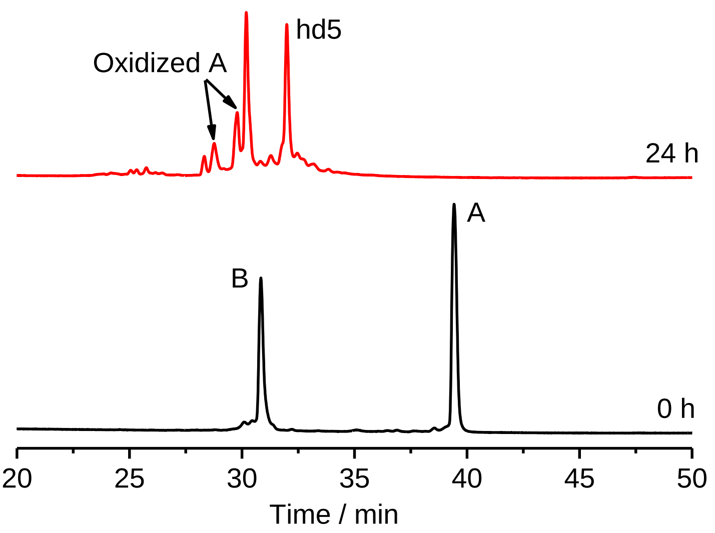

b)

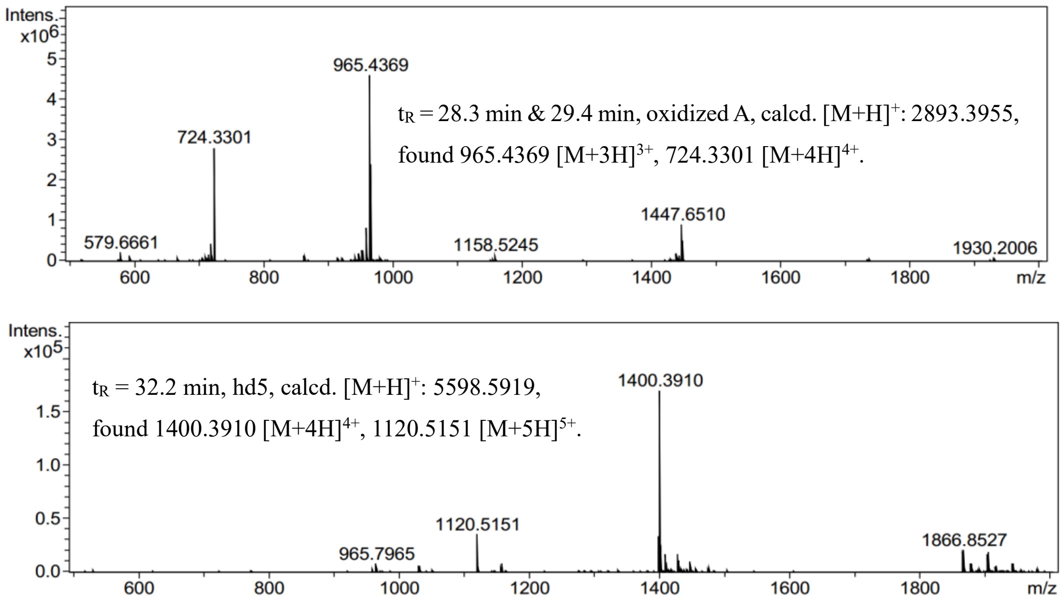

Supplementary Figure S18. Dimerization of hd5. a) Chromatograms showing the dimerization of hd5. A: Pen-bearing monomer, B: Cys-bearing monomer. Reaction conditions: 120 µM A, 100 µM B, 50 µM SeCys, 100 mM Gly-NaOH buffer (pH 10.0), at 37 °C for 24 hours. **b)** Mass spectra of the products formed after the dimerization of hd5.

a)

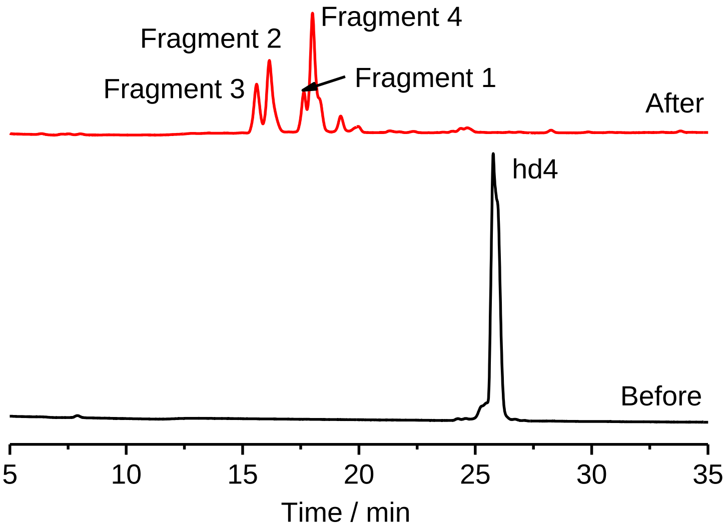
Fragment 4
Fragment 2
Fragment 1
Fragment 3
After
hd4
Before
5
10
15
20
25
30
35
Time / min

b)

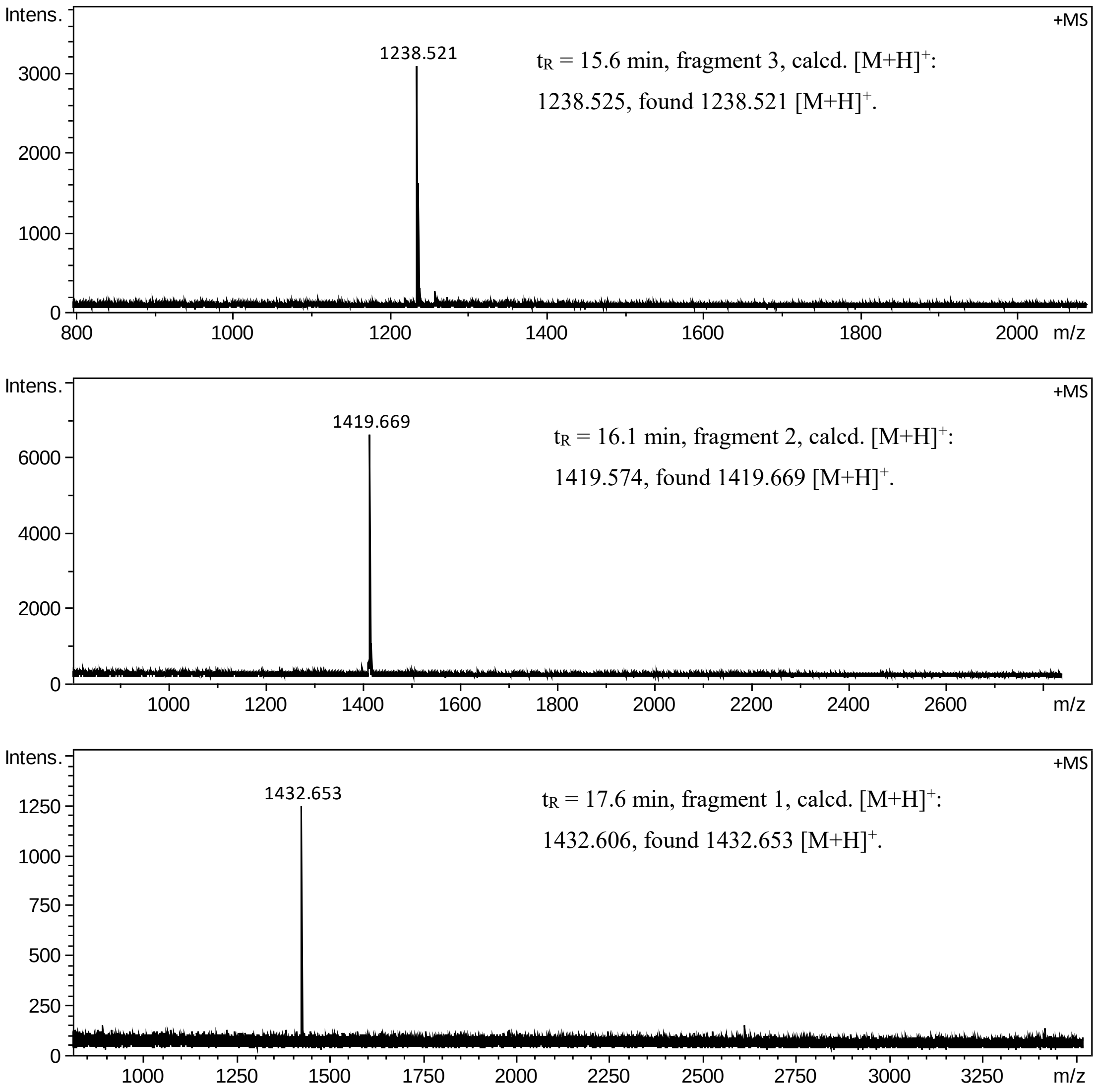
Intens.
+MS
1238.521
t_R = 15.6 min, fragment 3, calcd. $[M+H]^+$: 1238.525, found 1238.521 $[M+H]^+$.
m/z
Intens.
+MS
1419.669
t_R = 16.1 min, fragment 2, calcd. $[M+H]^+$: 1419.574, found 1419.669 $[M+H]^+$.
m/z
Intens.
+MS
1432.653
t_R = 17.6 min, fragment 1, calcd. $[M+H]^+$: 1432.606, found 1432.653 $[M+H]^+$.
m/z

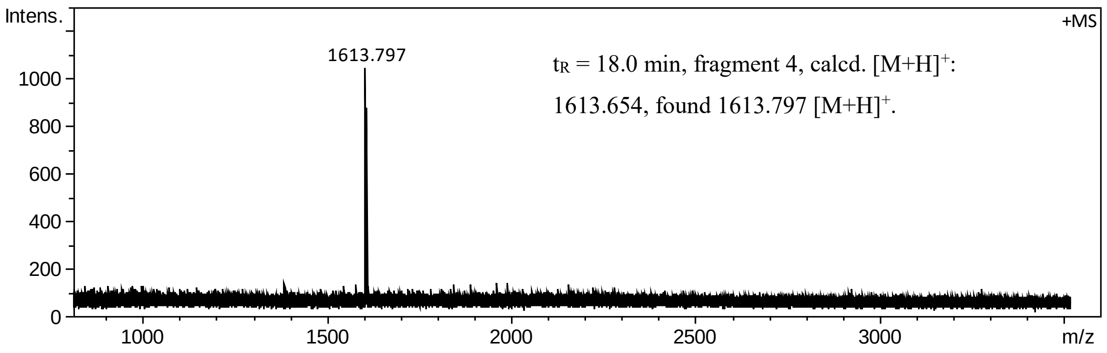

The arrows indicate the cleavage sites of trypsin digestion:

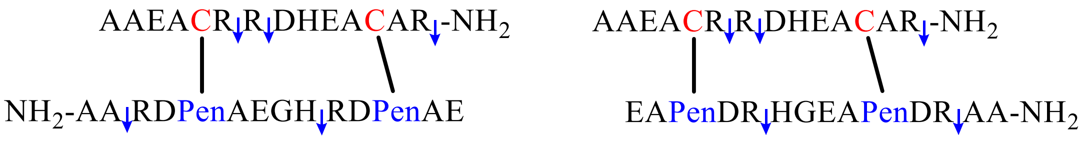

correctly-paired antiparallel heterodimer

undesired parallel heterodimer

Fragments analysis:

Fragment NO.	Fragment sequence
Fragment 1	AAEACR<br>RDPenAEGH
Fragment 2	DHEACAR<br>RDPenAE
Fragment 3	AAEACR<br>EAPenDR
Fragment 4	DHEACAR<br>HGEAPenDR

Supplementary Figure S19. Trypsin digestion LC-MS analysis of hd4. a) Chromatograms showing the digested fragments from hd4. **b)** Mass spectra of the digested fragments labeled in the chromatograms.

a)

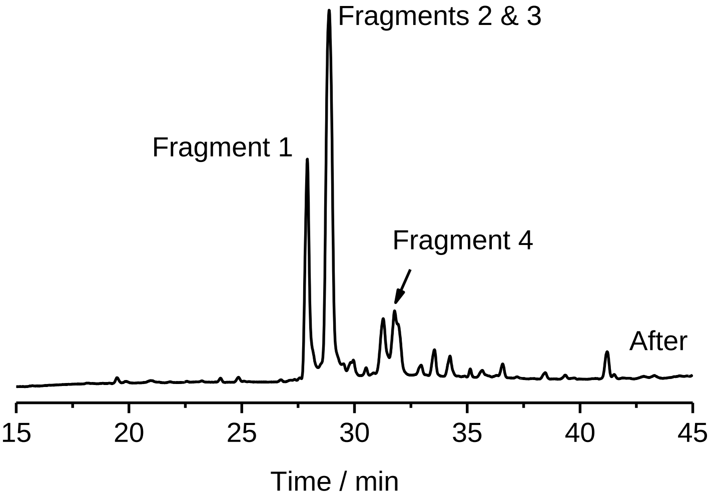

b)

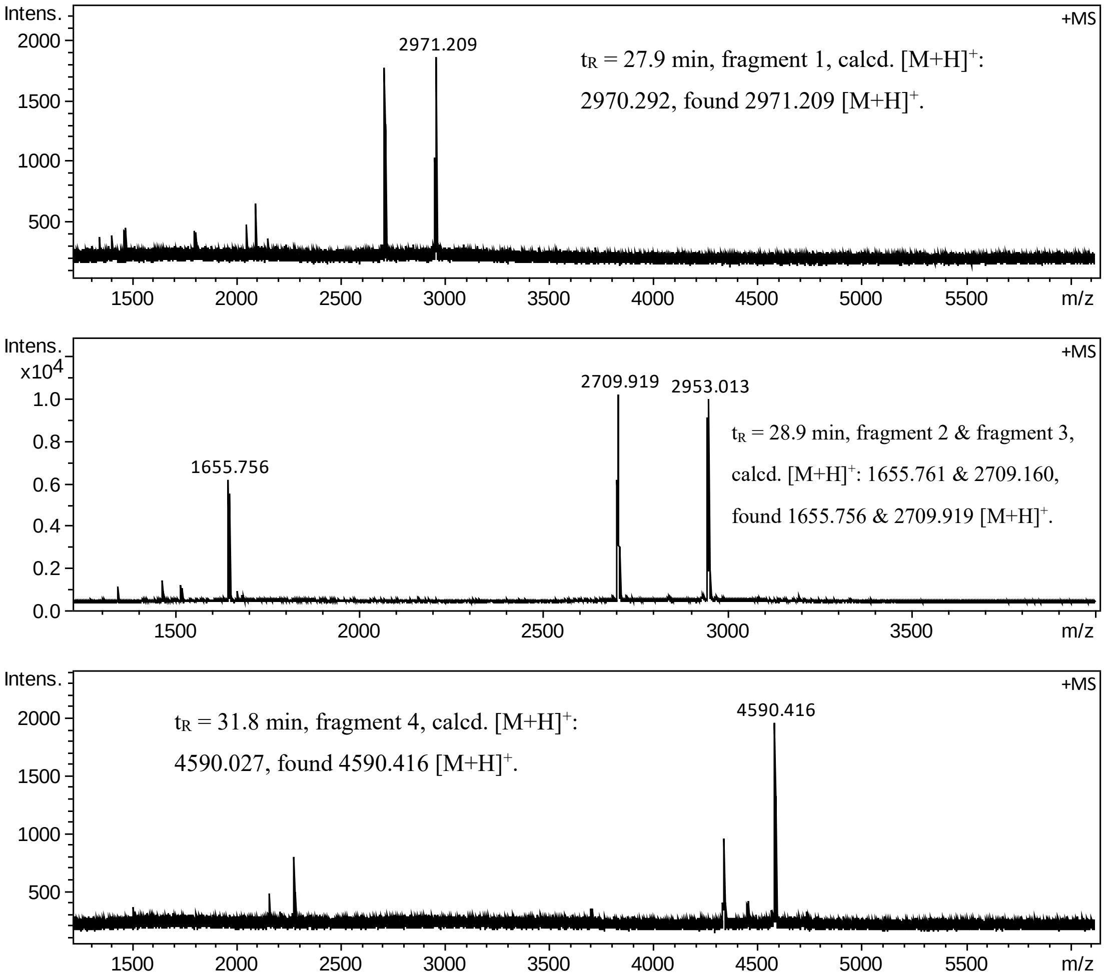

The arrows indicate the cleavage sites of trypsin digestion:

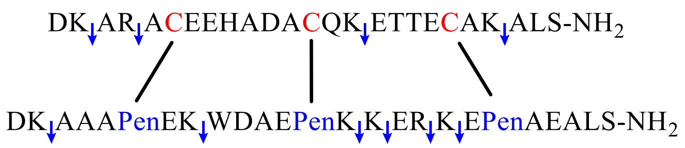

Fragments analysis:

Fragment NO.	Fragment sequence
Fragment 1	ACEEHADACQK<br>DKAAAPenEK WDAEPenKK
Fragment 2	ETTECAK<br>KEPenAEALS-NH_2
Fragment 3	ACEEHADACQK<br>AAAPenEKWDAEPenKK
Fragment 4	ACEEHADACQKETTECAK<br>DKAAAPenEKWDAEPenKK KEPenAEALS-NH_2

Supplementary Figure S20. Trypsin digestion LC-MS analysis of hd5. a) Chromatogram showing the digested fragments from hd5. **b)** Mass spectra of the digested fragments labeled in the chromatogram.

a)

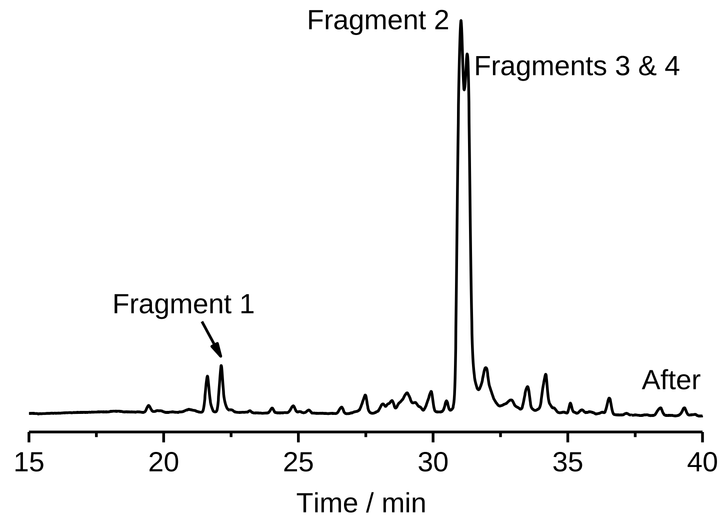

b)

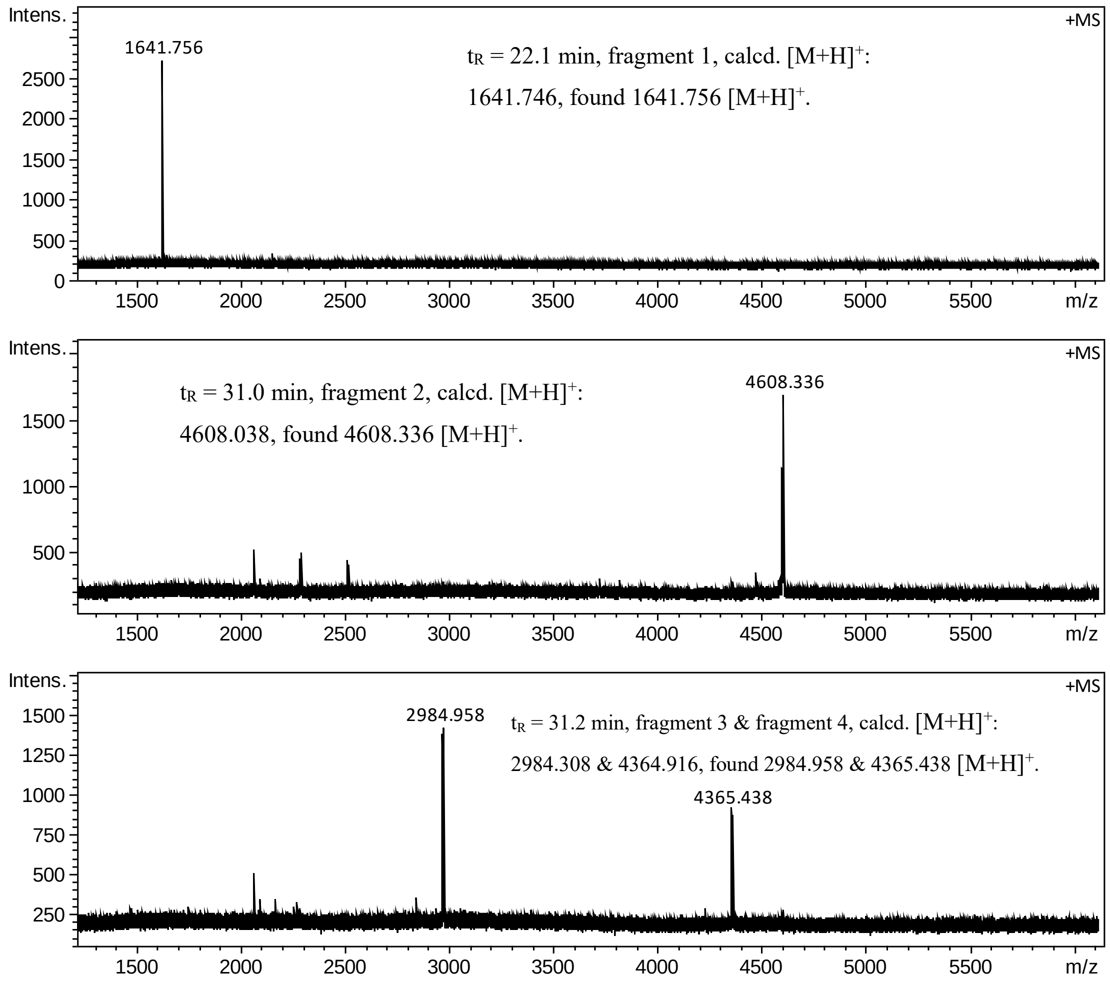

The arrows indicate the cleavage sites of trypsin digestion:

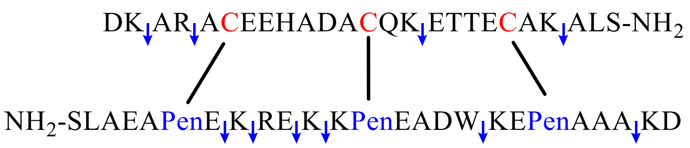

Fragments analysis:

Fragment NO.	Fragment sequence
Fragment 1	ETTECAK<br>KEPenAAAKD
Fragment 2	ACEEHADACQKETTECAK<br>NH_2-SLAEAPenEK KKPenEADW KEPenAAAKD<br>or<br>ACEEHADACQK ETTECAK<br>NH_2-SLAEAPenEK KKPenEADWKEPenAAAKD
Fragment 3	ACEEHADACQK<br>NH_2-SLAEAPenEK KKPenEADW
Fragment 4	ACEEHADACQKETTECAK<br>NH_2-SLAEAPenEK KKPenEADW KEPenAAA<br>or<br>ACEEHADACQK ETTECAK<br>NH_2-SLAEAPenEK KKPenEADWKEPenAAA

Supplementary Figure S21. Trypsin digestion LC-MS analysis of the undesired antiparallel heterodimer for hd5. a) Chromatogram showing the digested fragments from the undesired antiparallel heterodimer. **b)** Mass spectra of the digested fragments labeled in the chromatogram.

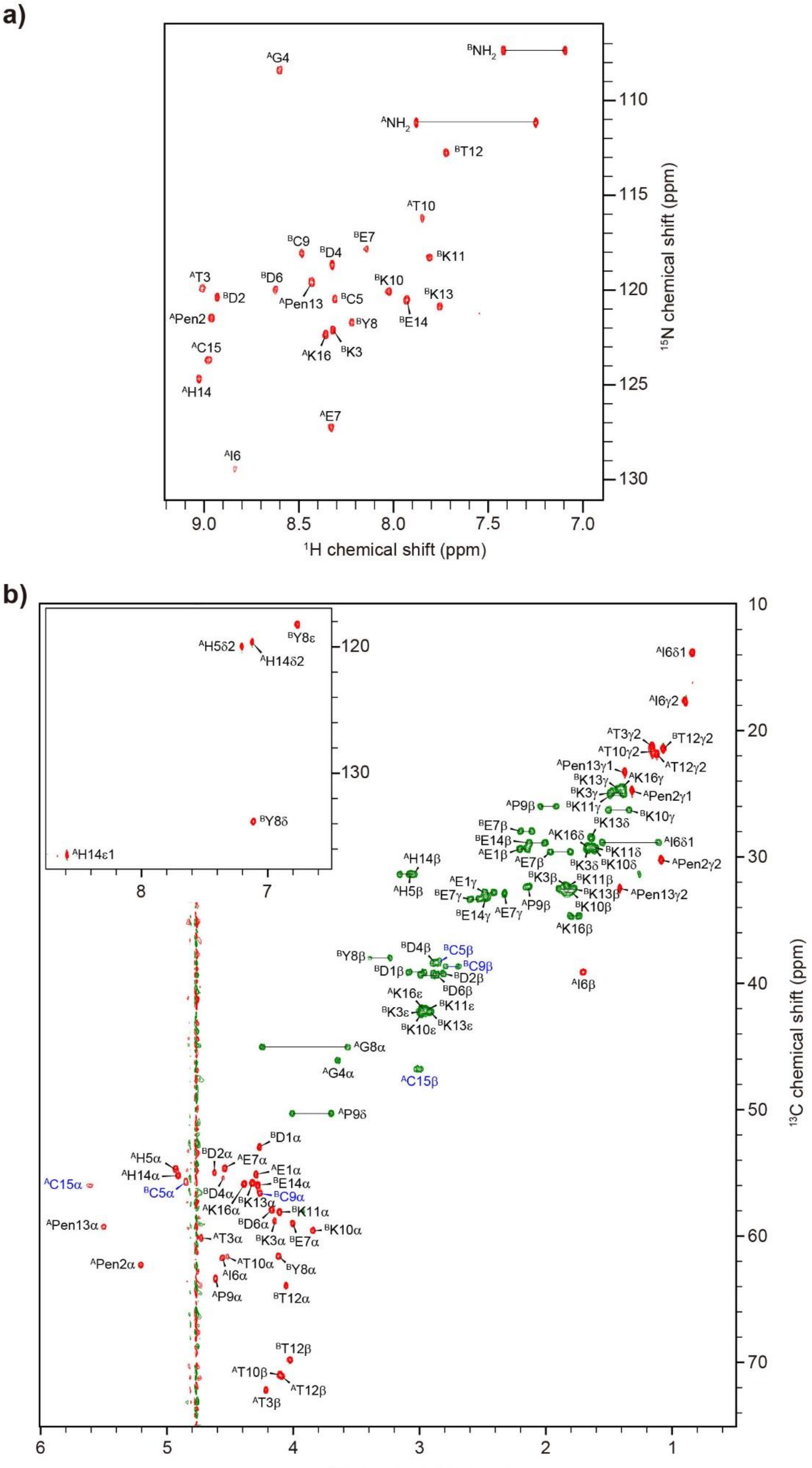

a)
b)
^{1}H chemical shift (ppm)
^{15}N chemical shift (ppm)
^{13}C chemical shift (ppm)
9.0 8.5 8.0 7.5 7.0
110 115 120 125 130
6 5 4 3 2 1
10 20 30 40 50 60 70
8 7
120 130

c)

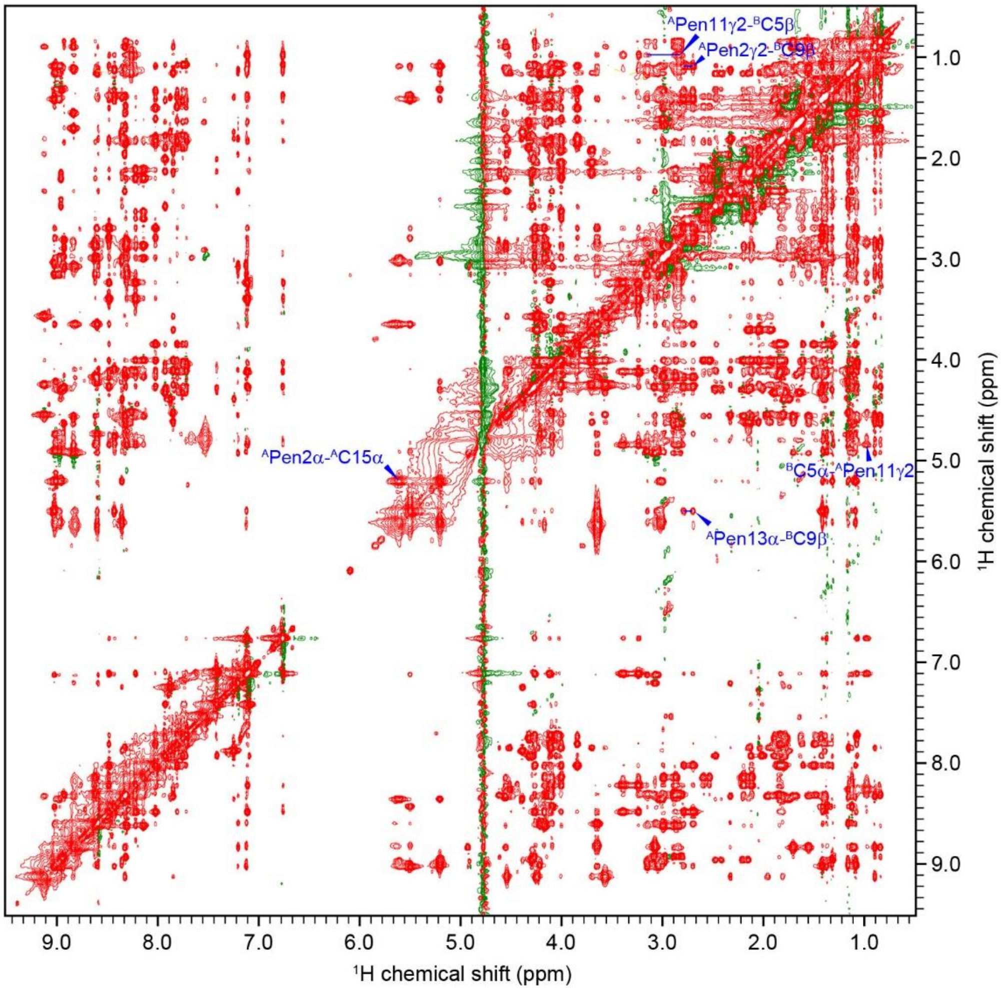

Supplementary Figure S22. 2D NMR spectra showing the disulfide-bonded heterodimerization of hd1. **a)** ^{1}H-^{15}N HSQC spectrum of hd1. The superscripts A and B represent assigned amino acid residues from A and B chains of hd1, respectively. The well dispersed signals and the homogenous signal intensities indicate the well folded peptide structure. **b)** Edited ^{1}H-^{13}C HSQC spectrum with opposite phases for CH_2 (green) and CH/CH_3 (red) signals. The Cβ chemical shifts of all three Cys residues in hd1 are in the range of oxidized chemical shifts (A chain: 46.8 ppm for Cys15-Cβ; B chain: 38.4 ppm for Cys5-Cβ and 38.7 ppm for Cys9-Cβ).[1] Moreover, the Cys15 in A chain and the Cys5/Cys9 in B chain show β-strand and helical Cβ chemical shifts, respectively, which are in consistent with the designed hd1 structure. **c)** ^{1}H-^{1}H NOESY spectrum with a mixing time of 200 ms. Crosspeaks between ^{A}Pen2-Hα and ^{A}C15-Hα, ^{A}Pen13-Hα and ^{B}C9-Hβ, and ^{B}C5-Hα and ^{A}Pen11γ were observed clearly, suggesting the disulfide bonds are probably formed between the corresponding residues, which drives the heterodimerization of hd1.

a)

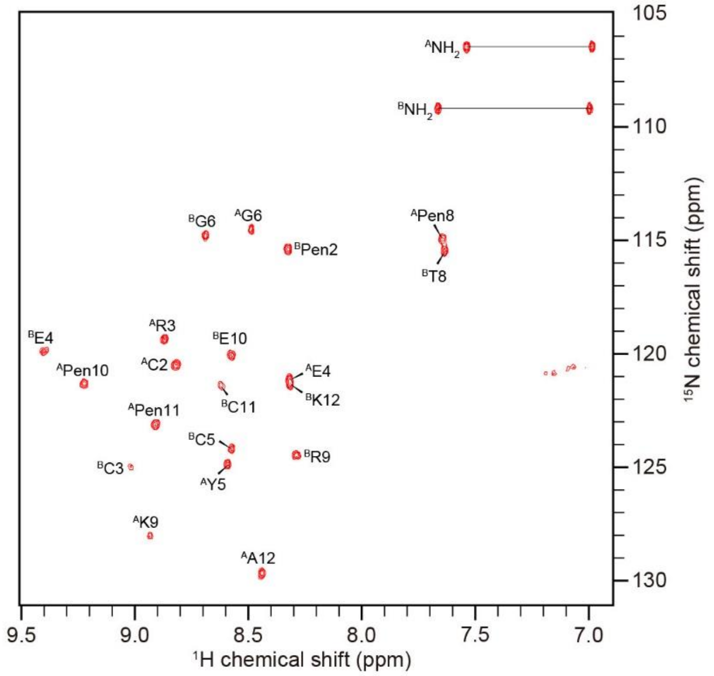

^{A}NH$_{2}$
^{B}NH$_{2}$
^{B}G6
^{A}G6
^{A}Pen8
^{B}Pen2
^{B}T8
^{A}R3
^{B}E4
^{B}E10
^{A}Pen10
^{A}C2
^{A}E4
^{B}K12
^{A}Pen11
^{B}C11
^{B}C5
^{B}R9
^{B}C3
^{A}Y5
^{A}K9
^{A}A12
9.5
9.0
8.5
8.0
7.5
7.0
^{1}H chemical shift (ppm)
105
110
115
120
125
130
^{15}N chemical shift (ppm)

b)

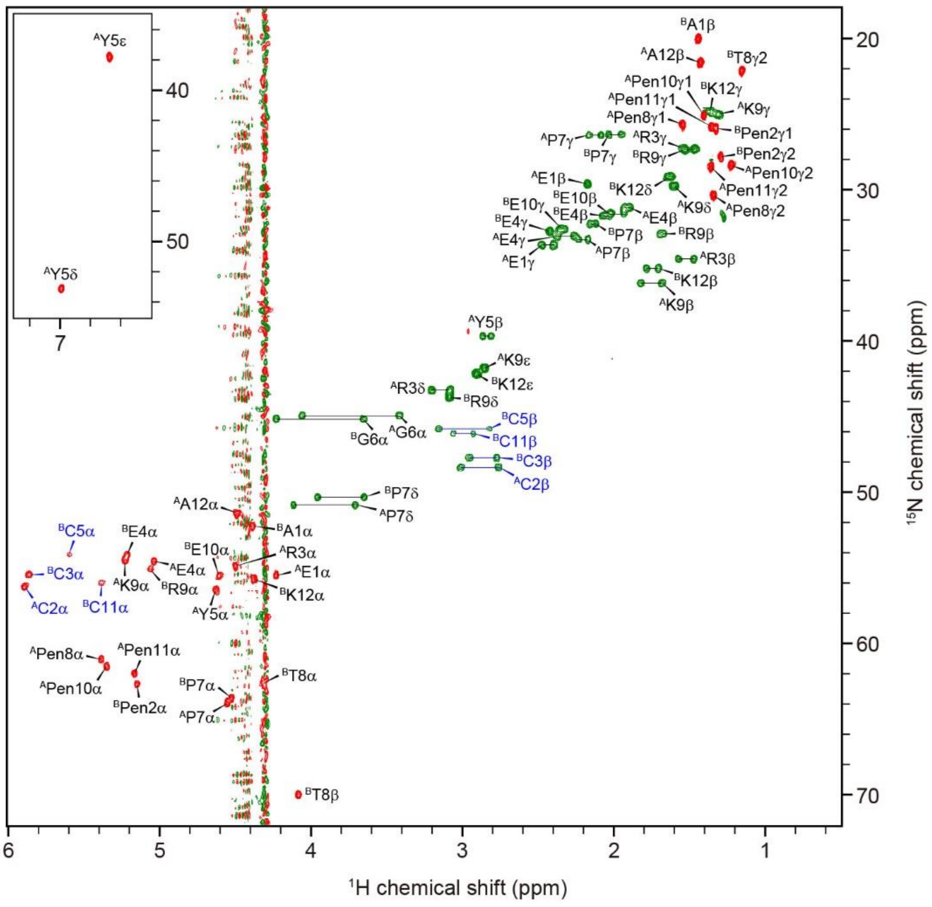

^{A}Y5ε
^{A}Y5δ
40
50
7
^{B}A1β
^{A}A12β
^{B}T8γ2
^{A}Pen10γ1
^{B}K12γ
^{A}Pen11γ1
^{A}K9γ
^{A}Pen8γ1
^{B}Pen2γ1
^{A}P7γ
^{A}R3γ
^{B}P7γ
^{B}R9γ
^{B}Pen2γ2
^{A}Pen10γ2
^{A}E1β
^{B}K12δ
^{A}Pen11γ2
^{B}E10β
^{B}E10γ
^{A}K9δ
^{A}Pen8γ2
^{B}E4β
^{A}E4β
^{B}E4γ
^{B}P7β
^{B}R9β
^{A}E4γ
^{A}P7β
^{A}R3β
^{A}E1γ
^{B}K12β
^{A}K9β
^{A}Y5β
^{A}K9ε
^{B}K12ε
^{A}R3δ
^{B}R9δ
^{B}C5β
^{B}G6α
^{A}G6α
^{B}C11β
^{B}C3β
^{A}C2β
^{B}P7δ
^{A}P7δ
^{A}A12α
^{B}A1α
^{B}C5α
^{B}E4α
^{B}E10α
^{A}R3α
^{B}C3α
^{A}E4α
^{A}E1α
^{A}K9α
^{B}R9α
^{B}K12α
^{A}C2α
^{B}C11α
^{A}Y5α
^{A}Pen8α
^{A}Pen11α
^{B}T8α
^{A}Pen10α
^{B}P7α
^{B}Pen2α
^{A}P7α
^{B}T8β
6
5
4
3
2
1
^{1}H chemical shift (ppm)
20
30
40
50
60
70
^{15}N chemical shift (ppm)

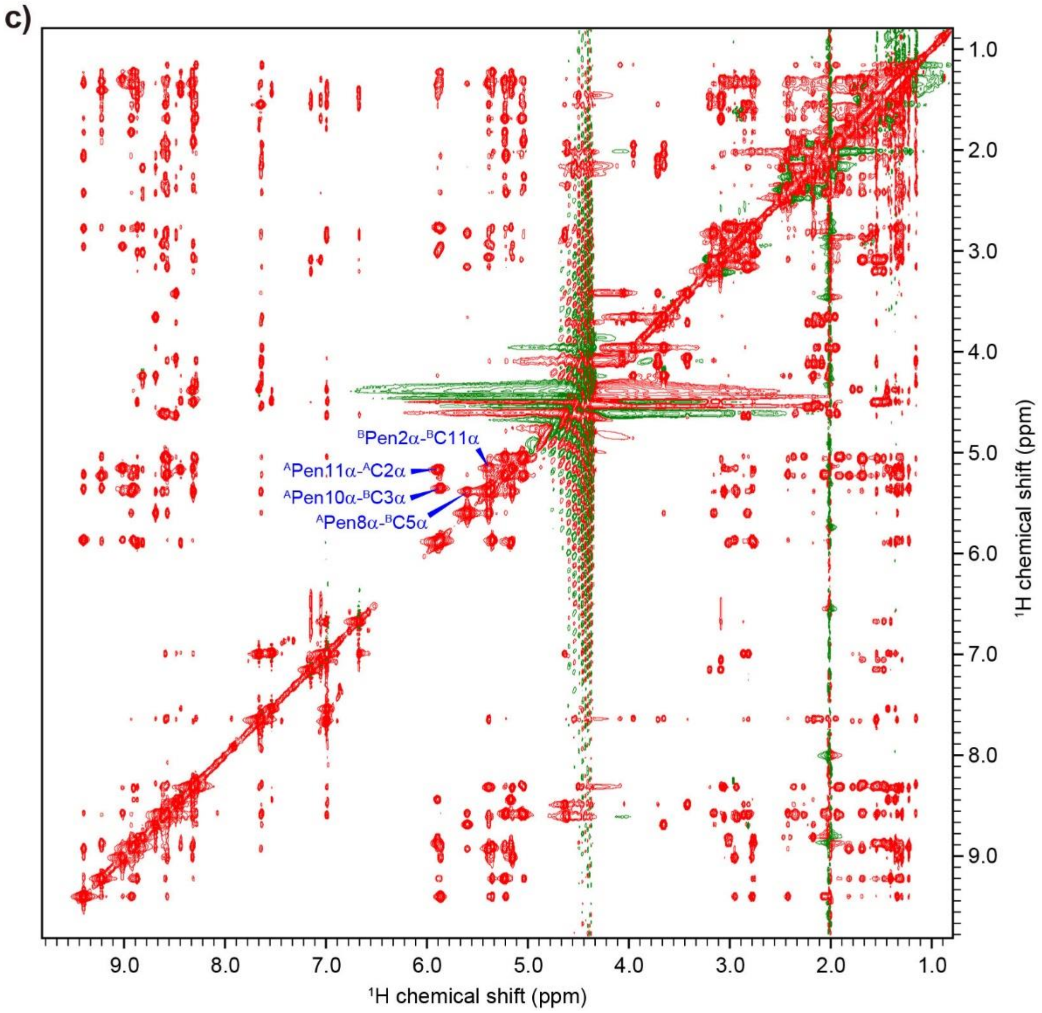

Supplementary Figure S23. 2D NMR spectra showing the disulfide-bonded heterodimerization of hd2. **a)** ^{1}H-^{15}N HSQC spectrum of hd2. The superscripts A and B represent assigned amino acid residues from A and B chains of hd2, respectively. The well dispersed signals and the homogenous signal intensities indicate the well folded peptide structure. **b)** Edited ^{1}H-^{13}C HSQC spectrum with opposite phases for CH_2 (green) and CH/CH_3 (red) signals. The Cβ chemical shifts of all four Cys residues in hd2 are in the range of oxidized chemical shifts (A chain: 48.4 ppm for Cys2-Cβ; B chain: 47.7 ppm for Cys3-Cβ, 45.8 ppm for Cys5-Cβ, and 46.2 ppm for Cys11-Cβ).[1] Moreover, all cysteines in hd2 show β-strand chemical shifts, which are in consistent with the designed peptide structure. **c)** ^{1}H-^{1}H NOESY spectrum with a mixing time of 300 ms. Crosspeaks between ^{A}Pen8-Hα and ^{B}C5-Hα, ^{A}Pen10-Hα and ^{B}C3-Hα, ^{A}Pen11-Hα and ^{A}C2-Hα, and ^{B}C5-Hα and ^{A}Pen11α were observed clearly, suggesting the disulfide bonds are formed between the corresponding residues, which drives the heterodimerization of hd2.

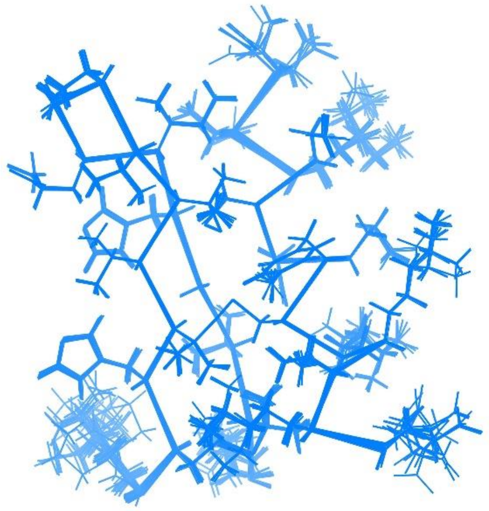

Supplementary Figure S24. A cross-eye stereo of the superimposed 20 lowest energy structures of hd1.

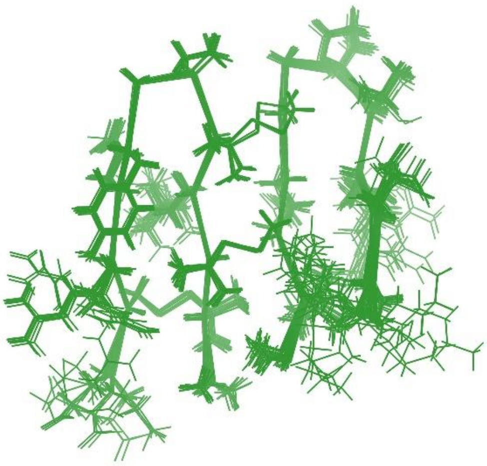

Supplementary Figure S25. A cross-eye stereo of the superimposed 20 lowest energy structures of hd2.

a)

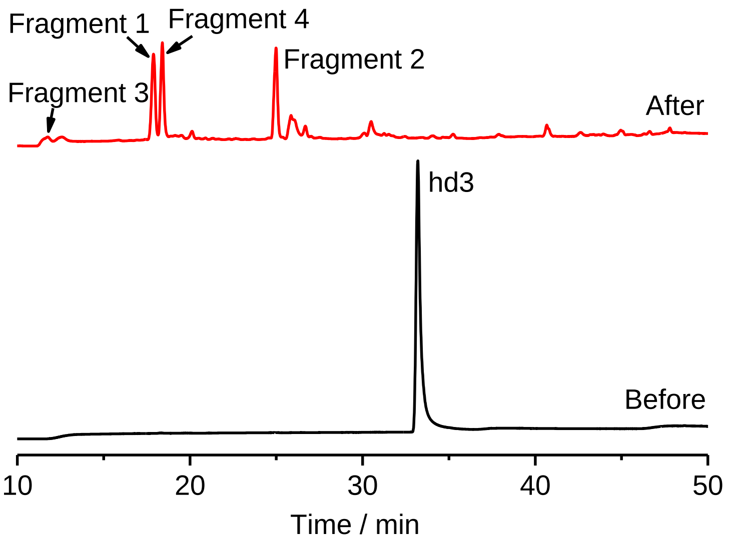
Fragment 1
Fragment 4
Fragment 2
Fragment 3
After
hd3
Before
10
20
30
40
50
Time / min

b)

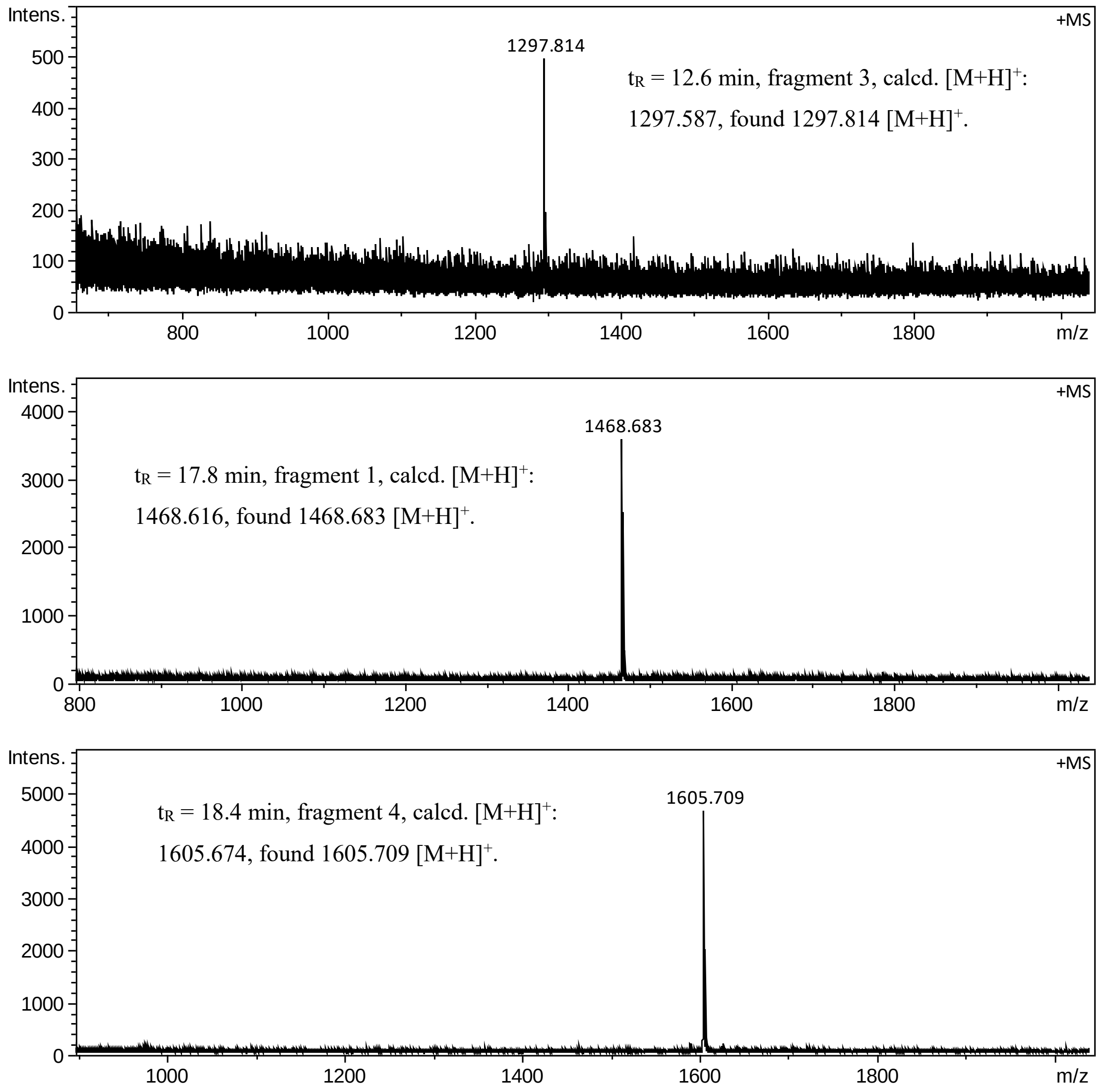
Intens.
+MS
1297.814
tR = 12.6 min, fragment 3, calcd. [M+H]+: 1297.587, found 1297.814 [M+H]+.
m/z
1468.683
tR = 17.8 min, fragment 1, calcd. [M+H]+: 1468.616, found 1468.683 [M+H]+.
1605.709
tR = 18.4 min, fragment 4, calcd. [M+H]+: 1605.674, found 1605.709 [M+H]+.

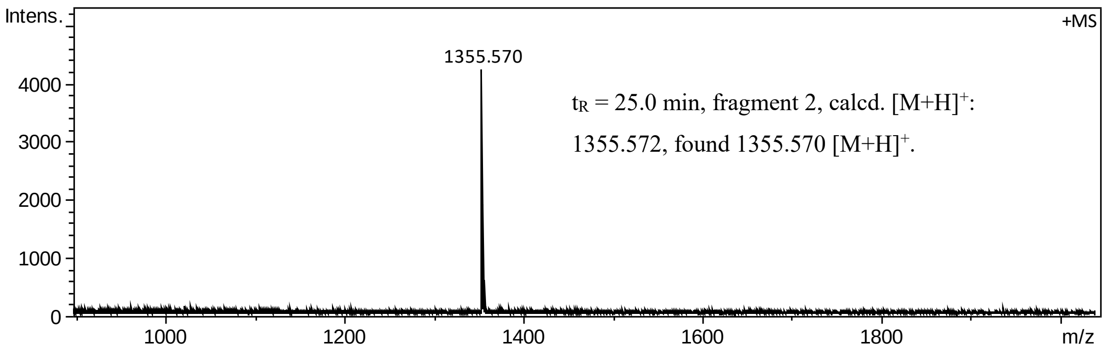

The arrows indicate the cleavage sites of trypsin digestion:

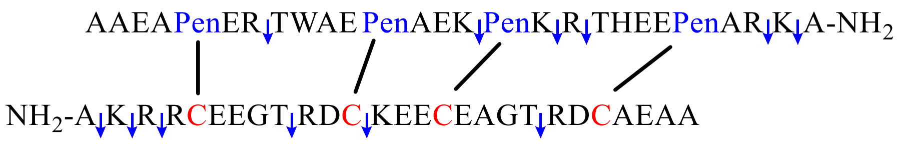

Fragments analysis:

Fragment NO.	Fragment sequence
Fragment 1	AAEAPenER | RCEEGT
Fragment 2	TWAE PenAEK | RDC
Fragment 3	PenKR | KEECEAGT
Fragment 4	THEEPenAR | RDCAEAA

Supplementary Figure S26. Trypsin digestion LC-MS analysis of hd3. a) Chromatograms showing the digested fragments from hd3. **b)** Mass spectra of the digested fragments labeled in the chromatograms.

1.4 Orthogonality of peptide heterodimerizations

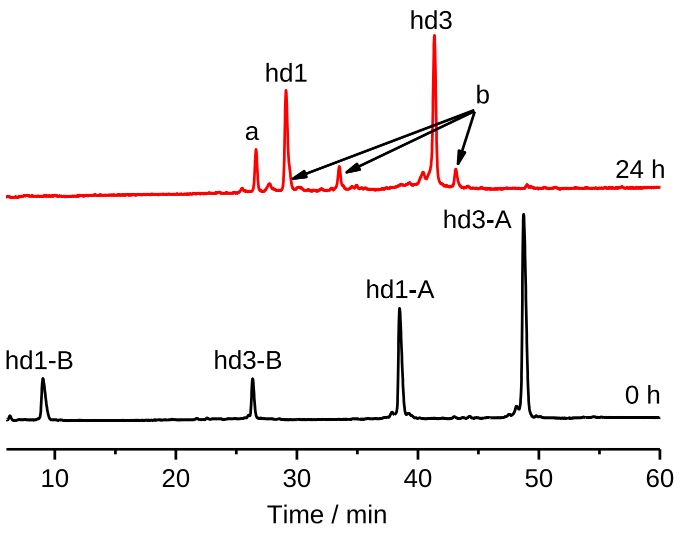

Peaks identification:

Peak NO.	Product	m/z calcd.	m/z found
a	Oxidized hd1-A	$[M+H]^+$: 1797.7539	$[M+2H]^{2+}$: 899.3691<br>$[M+3H]^{3+}$: 599.9147
	hd1	$[M+H]^+$: 3515.4711	$[M+3H]^{3+}$: 1172.4806<br>$[M+4H]^{4+}$: 879.6126
	hd3	$[M+H]^+$: 6171.7677	$[M+4H]^{4+}$: 1544.0036<br>$[M+5H]^{5+}$: 1235.1425<br>$[M+6H]^{6+}$: 1029.4539
b	Oxidized hd3-A	$[M+H]^+$: 3188.5044	$[M+3H]^{3+}$: 1064.1620<br>$[M+4H]^{4+}$: 798.3729

Supplementary Figure S27. HPLC chromatograms and peaks identification of orthogonal reaction between hd1 and hd3. A: Pen-bearing monomer, B: Cys-bearing monomer. Reaction conditions: 100 µM A of two heterodimers, 50 µM B of two heterodimers, 50 µM SeCys, 100 mM Gly-NaOH buffer (pH 10.0), at 37 °C for 24 hours.

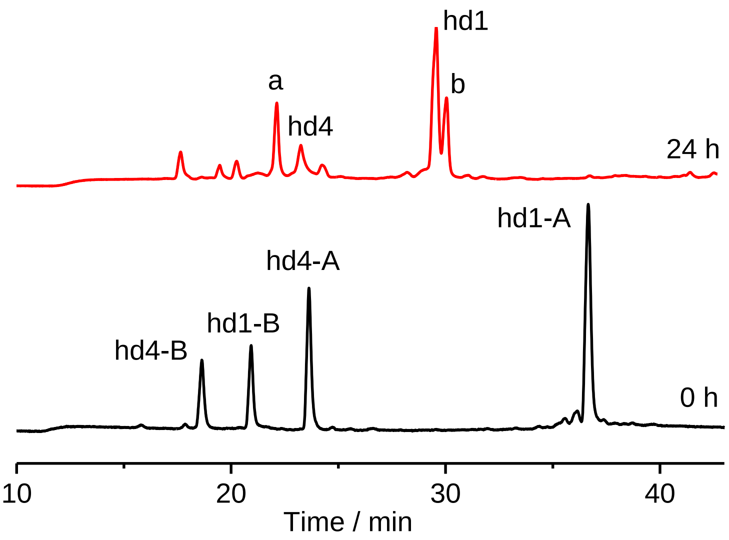

Peaks identification:

Peak NO.	Product	m/z calcd.	m/z found
a	Oxidized hd4-A	$[M+H]^+$: 1556.6693	$[M+2H]^{2+}$: 778.9113
	hd4	$[M+H]^+$: 3112.3611	$[M+3H]^{3+}$: 1038.2232<br>$[M+4H]^{4+}$: 778.9192
	hd1	$[M+H]^+$: 3515.4711	$[M+3H]^{3+}$: 1172.6037<br>$[M+4H]^{4+}$: 879.7057
b	Oxidized hd1-A	$[M+H]^+$: 1797.7539	$[M+2H]^{2+}$: 899.4631

Supplementary Figure S28. HPLC chromatograms and peaks identification of orthogonal reaction between hd1 and hd4. A: Pen-bearing monomer, B: Cys-bearing monomer. Reaction conditions: 100 μM A of two heterodimers, 50 μM B of two heterodimers, 50 μM SeCys, 50 mM Tris buffer (pH 8.5), at 37 °C for 24 hours.

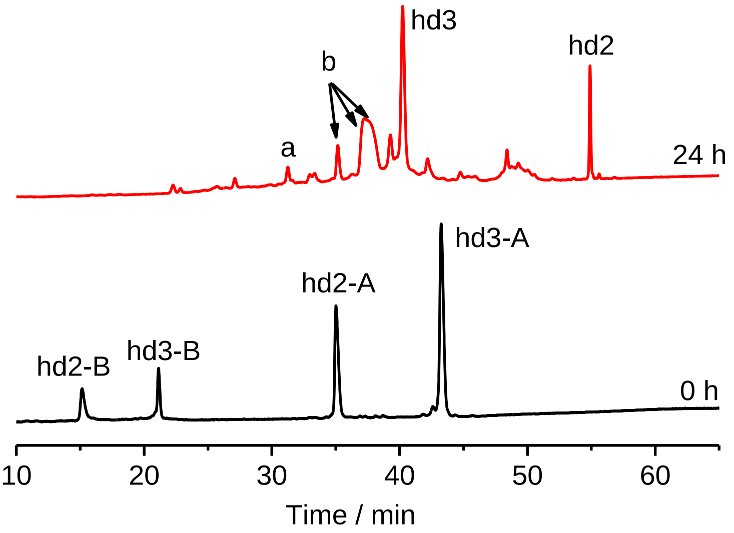

Peaks identification:

Peak NO.	Product	m/z calcd.	m/z found
a	Oxidized hd3-A	$[M+H]^+$: 3188.5044	$[M+3H]^{3+}$: 1063.4112<br>$[M+4H]^{4+}$: 798.0621
b	Oxidized hd2-A	$[M+H]^+$: 1440.5891	$[M+2H]^{2+}$: 720.7334
	hd3	$[M+H]^+$: 6171.7677	$[M+5H]^{5+}$: 1235.0464
	hd2	$[M+H]^+$: 2764.0955	$[M+3H]^{3+}$: 921.9538

Supplementary Figure S29. HPLC chromatograms and peaks identification of orthogonal reaction between hd2 and hd3. A: Pen-bearing monomer, B: Cys-bearing monomer. Reaction conditions: 100 µM A of two heterodimers, 50 µM B of two heterodimers, 50 µM SeCys, 50 mM Tris buffer (pH 8.5), at 37 °C for 24 hours.

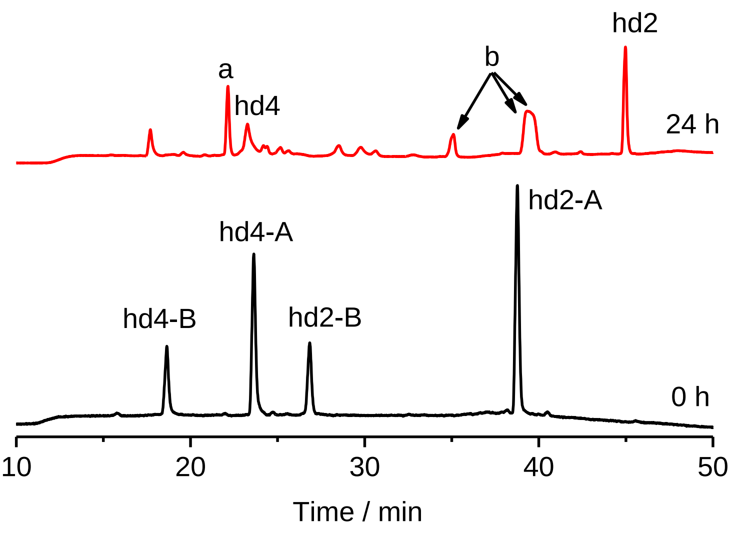

Peaks identification:

Peak NO.	Product	m/z calcd.	m/z found
a	Oxidized hd4-A	$[M+H]^+$: 1556.6693	$[M+2H]^{2+}$: 778.9073
	hd4	$[M+H]^+$: 3112.3611	$[M+3H]^{3+}$: 1038.2181<br>$[M+4H]^{4+}$: 778.9109
b	Oxidized hd2-A	$[M+H]^+$: 1440.5891	$[M+2H]^{2+}$: 720.8612
	hd2	$[M+H]^+$: 2764.0955	$[M+3H]^{3+}$: 921.7860

Supplementary Figure S30. HPLC chromatograms and peaks identification of orthogonal reaction between hd2 and hd4. A: Pen-bearing monomer, B: Cys-bearing monomer. Reaction conditions: 100 μM A of two heterodimers, 50 μM B of two heterodimers, 50 μM SeCys, 50 mM Tris buffer (pH 8.5), at 37 °C for 24 hours.

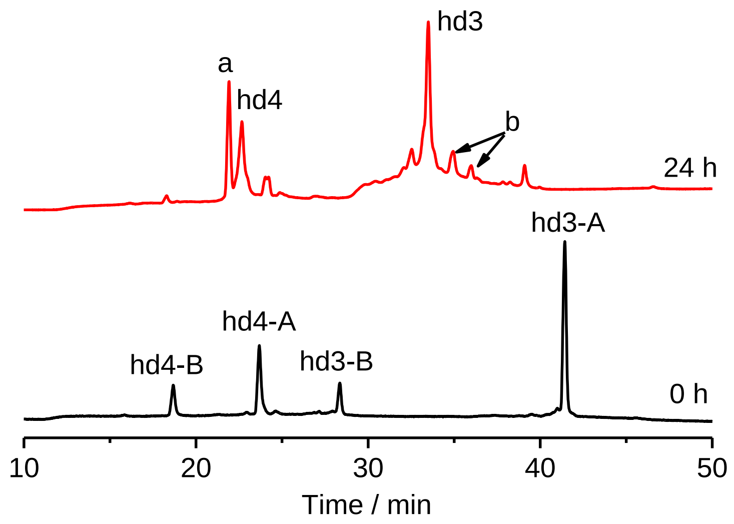

Peaks identification:

Peak NO.	Product	m/z calcd.	m/z found
a	Oxidized hd4-A	$[M+H]^+$: 1556.6693	$[M+2H]^{2+}$: 778.8669
	hd4	$[M+H]^+$: 3112.3611	$[M+3H]^{3+}$: 1037.7216<br>$[M+4H]^{4+}$: 778.7911
	hd3	$[M+H]^+$: 6171.7677	$[M+4H]^{4+}$: 1544.1426<br>$[M+5H]^{5+}$: 1235.5130
b	Oxidized hd3-A	$[M+H]^+$: 3188.5044	$[M+3H]^{3+}$: 1063.8754<br>$[M+4H]^{4+}$: 798.1564

Supplementary Figure S31. HPLC chromatograms and peaks identification of orthogonal reaction between hd3 and hd4. A: Pen-bearing monomer, B: Cys-bearing monomer. Reaction conditions: 100 μM A of two heterodimers, 50 μM B of two heterodimers, 50 μM SeCys, 50 mM Tris buffer (pH 8.5), at 37 °C for 24 hours.

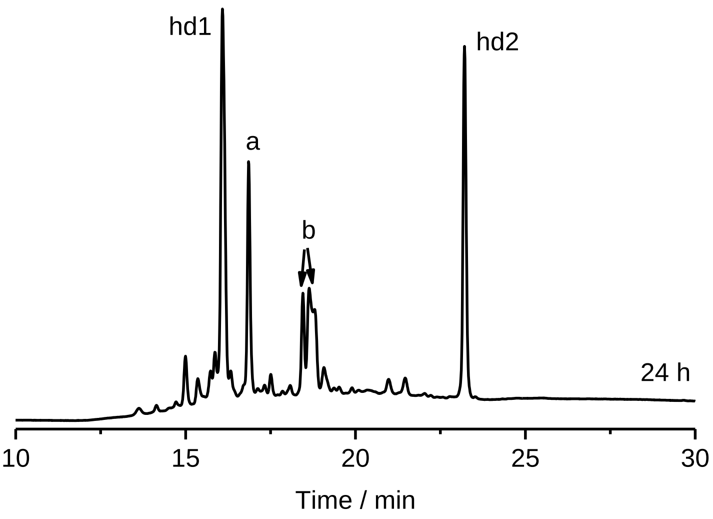

Peaks identification:

Peak NO.	Product	m/z calcd.	m/z found
	hd1	$[M+H]^+$: 3515.5	$[M+3H]^{3+}$: 1172.2
a	Incorrectly-paired heterodimer	$[M+H]^+$: 3158.3	$[M+2H]^{2+}$: 1578.9<br>$[M+3H]^{3+}$: 1053.2
b	Oxidized hd2-A	$[M+H]^+$: 1440.6	$[M+H]^+$: 1440.4<br>$[M+2H]^{2+}$: 720.7
	hd2	$[M+H]^+$: 2764.1	$[M+2H]^{2+}$: 1382.3<br>$[M+3H]^{3+}$: 921.9

Supplementary Figure S32. HPLC chromatogram and peaks identification of orthogonal reaction between hd1 and hd2. A: Pen-bearing monomer, B: Cys-bearing monomer. Reaction conditions: 100 μM A of two heterodimers, 50 μM B of two heterodimers, 50 μM SeCys, 100 mM phosphate buffer (pH 7.4), at 37 °C for 24 hours.

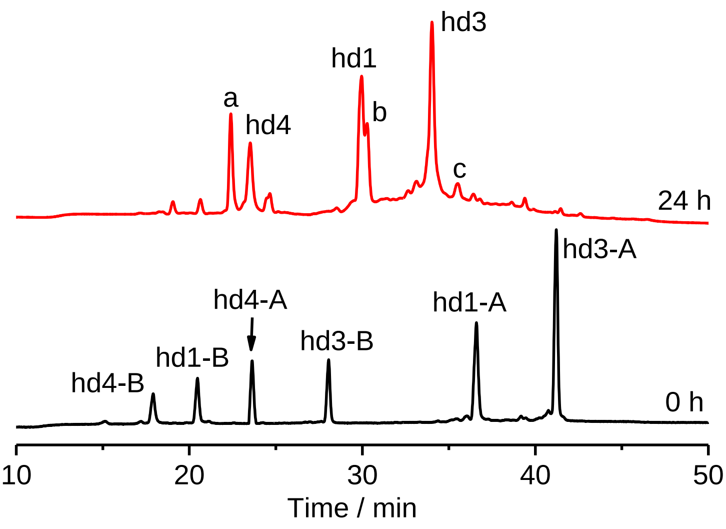

Peaks identification:

Peak NO.	Product	m/z calcd.	m/z found
a	Oxidized hd4-A	$[M+H]^+$: 1556.6693	$[M+2H]^{2+}$: 778.8773
	hd4	$[M+H]^+$: 3112.3611	$[M+3H]^{3+}$: 1037.8515<br>$[M+4H]^{4+}$: 778.6433
	hd1	$[M+H]^+$: 3515.4711	$[M+3H]^{3+}$: 1172.5542<br>$[M+4H]^{4+}$: 879.6704
b	Oxidized hd1-A	$[M+H]^+$: 1797.7539	$[M+2H]^{2+}$: 899.3597<br>$[M+3H]^{3+}$: 599.9103
	hd3	$[M+H]^+$: 6171.7677	$[M+5H]^{5+}$: 1235.2193<br>$[M+6H]^{6+}$: 1029.6862
c	Oxidized hd3-A	$[M+H]^+$: 3188.5044	$[M+3H]^{3+}$: 1063.4240<br>$[M+4H]^{4+}$: 798.0732

Supplementary Figure S33. HPLC chromatograms and peaks identification of orthogonal reaction between hd1, hd3 and hd4. A: Pen-bearing monomer, B: Cys-bearing monomer. Reaction conditions: 100 µM A of three heterodimers, 50 µM B of three heterodimers, 75 µM SeCys, 50 mM Tris buffer (pH 8.5), at 37 °C for 24 hours.

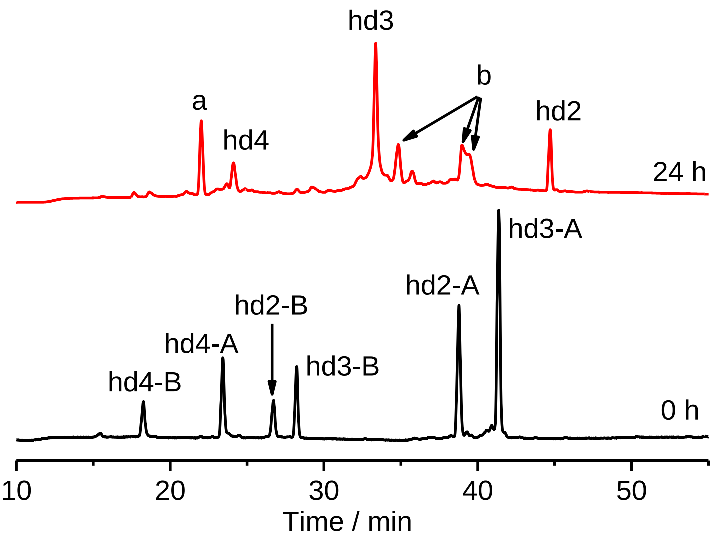

Peaks identification:

Peak NO.	Product	m/z calcd.	m/z found
a	Oxidized hd4-A	$[M+H]^+$: 1556.6693	$[M+2H]^{2+}$: 778.8085
	hd4	$[M+H]^+$: 3112.3611	$[M+3H]^{3+}$: 1037.7620<br>$[M+4H]^{4+}$: 779.3090
	hd3	$[M+H]^+$: 6171.7677	$[M+4H]^{4+}$: 1543.8979<br>$[M+5H]^{5+}$: 1235.5179
b	Oxidized hd2-A	$[M+H]^+$: 1440.5891	$[M+2H]^{2+}$: 720.7867
	hd2	$[M+H]^+$: 2764.095	$[M+3H]^{3+}$: 921.6917

Supplementary Figure S34. HPLC chromatograms and peaks identification of orthogonal reaction between hd2, hd3 and hd4. A: Pen-bearing monomer, B: Cys-bearing monomer. Reaction conditions: 100 µM A of three heterodimers, 50 µM B of three heterodimers, 75 µM SeCys, 50 mM Tris buffer (pH 8.5), at 37 °C for 24 hours.

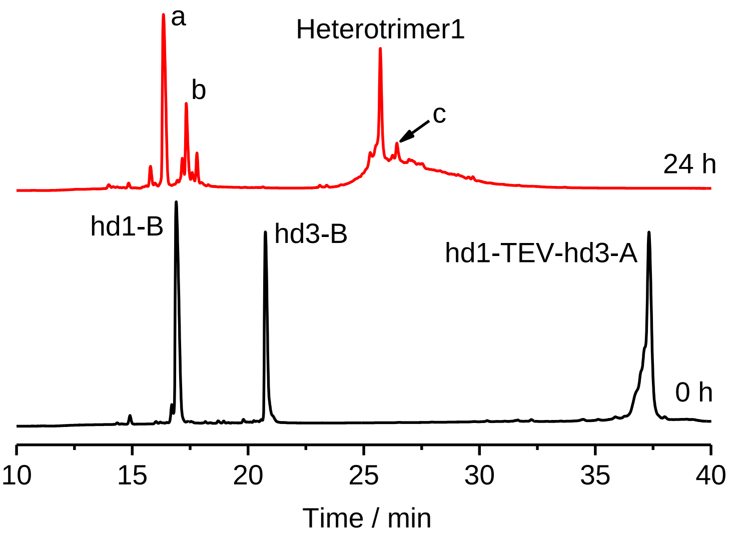

Peaks identification:

Peak NO.	Product	m/z calcd.	m/z found
a	Oxidized hd1-B	$[M+H]^+$: 1716.720	$[M+H]^+$: 1716.822
b	Oxidized hd3-B	$[M+H]^+$: 2983.270	$[M+H]^+$: 2983.370<br>$[M+2H]^{2+}$: 1492.693
	Heterotrimer1	$[M+H]^+$: 10521.588	$[M+H]^+$: 10522.022
c	Heterodimer	$[M+H]^+$: 8804.873<br>$[M+H]^+$: 7538.325	$[M+H]^+$: 8804.950<br>$[M+H]^+$: 7538.446

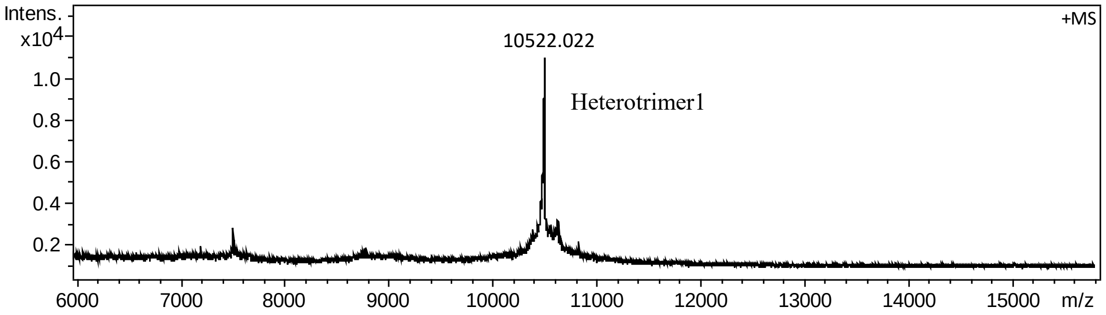

Supplementary Figure S35. HPLC chromatograms and peaks identification of oxidation-induced dimerization to form heterotrimer1. A: Pen-bearing monomer, B: Cys-bearing monomer. Reaction conditions: 100 µM hd1-TEV-hd3-A, 300 µM hd1-B, 150 µM hd3-B, 100 µM SeCys, 100 mM Gly-NaOH buffer (pH 10.0), at 37 °C for 24 hours.

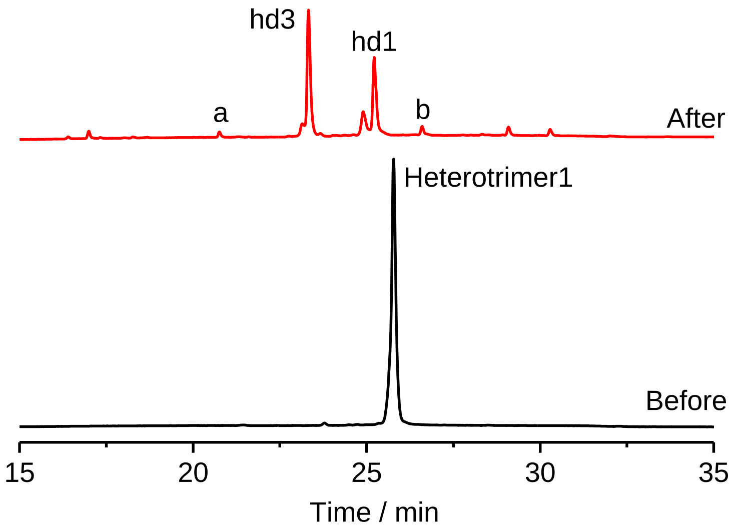

Peaks identification:

Peak NO.	Product	m/z calcd.	m/z found
a	hd3-B	$[M+H]^+$: 2987.301	$[M+H]^+$: 2987.193<br>$[M+2H]^{2+}$: 1494.130
	hd3	$[M+H]^+$: 6228.789	$[M+H]^+$: 6228.574<br>$[M+2H]^{2+}$: 3115.145
	hd1	$[M+H]^+$: 4310.816	$[M+H]^+$: 4311.726<br>$[M+2H]^{2+}$: 2156.497
b	Oxidized hd1-A<br>(single disulfide)	$[M+H]^+$: 2596.116	$[M+H]^+$: 2596.013

Supplementary Figure S36. HPLC chromatograms and peaks identification of heterotrimer1 digested by AcTEV^{TM} Protease. A: Pen-bearing monomer, B: Cys-bearing monomer.

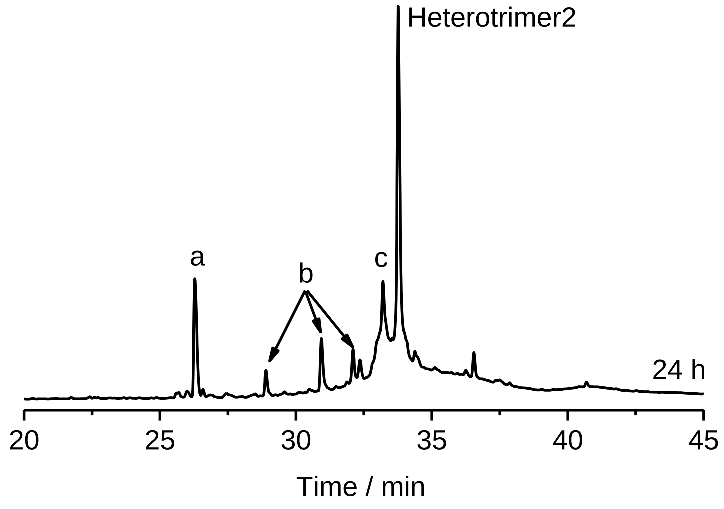

Peaks identification:

Peak NO.	Product	m/z calcd.	m/z found
a	Oxidized hd1-A	$[M+H]^+$: 1797.7539	$[M+2H]^{2+}$: 899.4375<br>$[M+3H]^{3+}$: 599.9620
b	Oxidized hd3-A	$[M+H]^+$: 3188.5044	$[M+3H]^{3+}$: 1063.9200<br>$[M+4H]^{4+}$: 797.9422
c	Heterodimer	$[M+H]^+$: 9011.9468	$[M+6H]^{6+}$: 1502.7682
	Heterotrimer2	$[M+H]^+$: 10810.6965	$[M+8H]^{8+}$: 1352.1637<br>$[M+10H]^{10+}$: 1081.9356

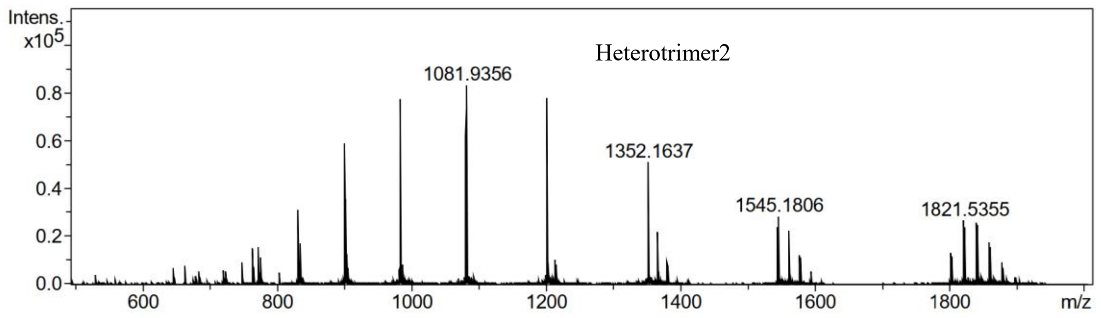

Supplementary Figure S37. HPLC chromatogram and peaks identification of oxidation-induced dimerization to form heterotrimer2. A: Pen-bearing monomer, B: Cys-bearing monomer. Reaction conditions: 500 μM hd1-A, 200 μM hd3-A, 100 μM hd1-TEV-hd3-B, 175 μM SeCys, 100 mM Gly-NaOH buffer (pH 10.0), at 37 °C for 24 hours.

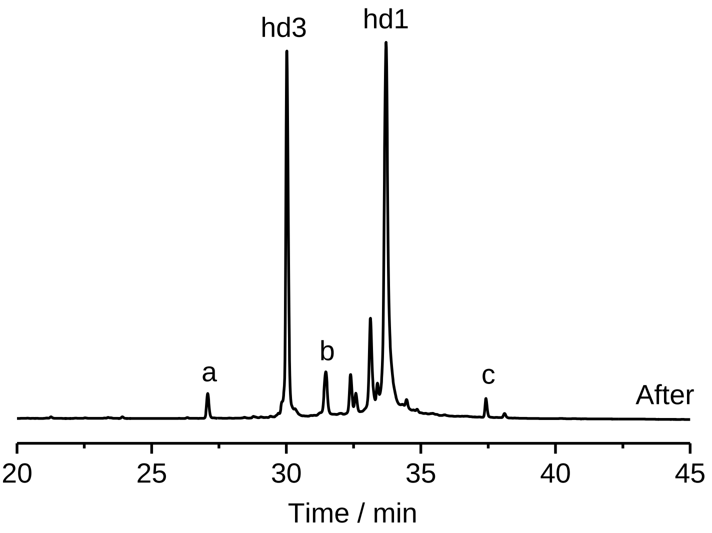

Peaks identification:

Peak NO.	Product	m/z calcd.	m/z found
a	Oxidized hd1-A (single disulfide)	$[M+H]^{+}$: 1799.7696	$[M+2H]^{2+}$: 900.4020<br>$[M+3H]^{3+}$: 600.6049
	hd3	$[M+H]^{+}$: 6373.8442	$[M+5H]^{5+}$: 1275.3938<br>$[M+6H]^{6+}$: 1063.1634
b	Oxidized hd1-B	$[M+H]^{+}$: 2657.1207	$[M+2H]^{2+}$: 1329.5879<br>$[M+3H]^{3+}$: 886.7293
	hd1	$[M+H]^{+}$: 4454.8691	$[M+3H]^{3+}$: 1485.9591<br>$[M+4H]^{4+}$: 1114.4717
c	hd3-A	$[M+H]^{+}$: 3192.5357	$[M+3H]^{3+}$: 1065.1931<br>$[M+5H]^{5+}$: 637.3087

Supplementary Figure S38. HPLC chromatogram and peaks identification of heterotrimer2 digested by AcTEV^{TM} Protease. A: Pen-bearing monomer, B: Cys-bearing monomer.

1.5 Potential applications of peptide heterodimerizations

a)

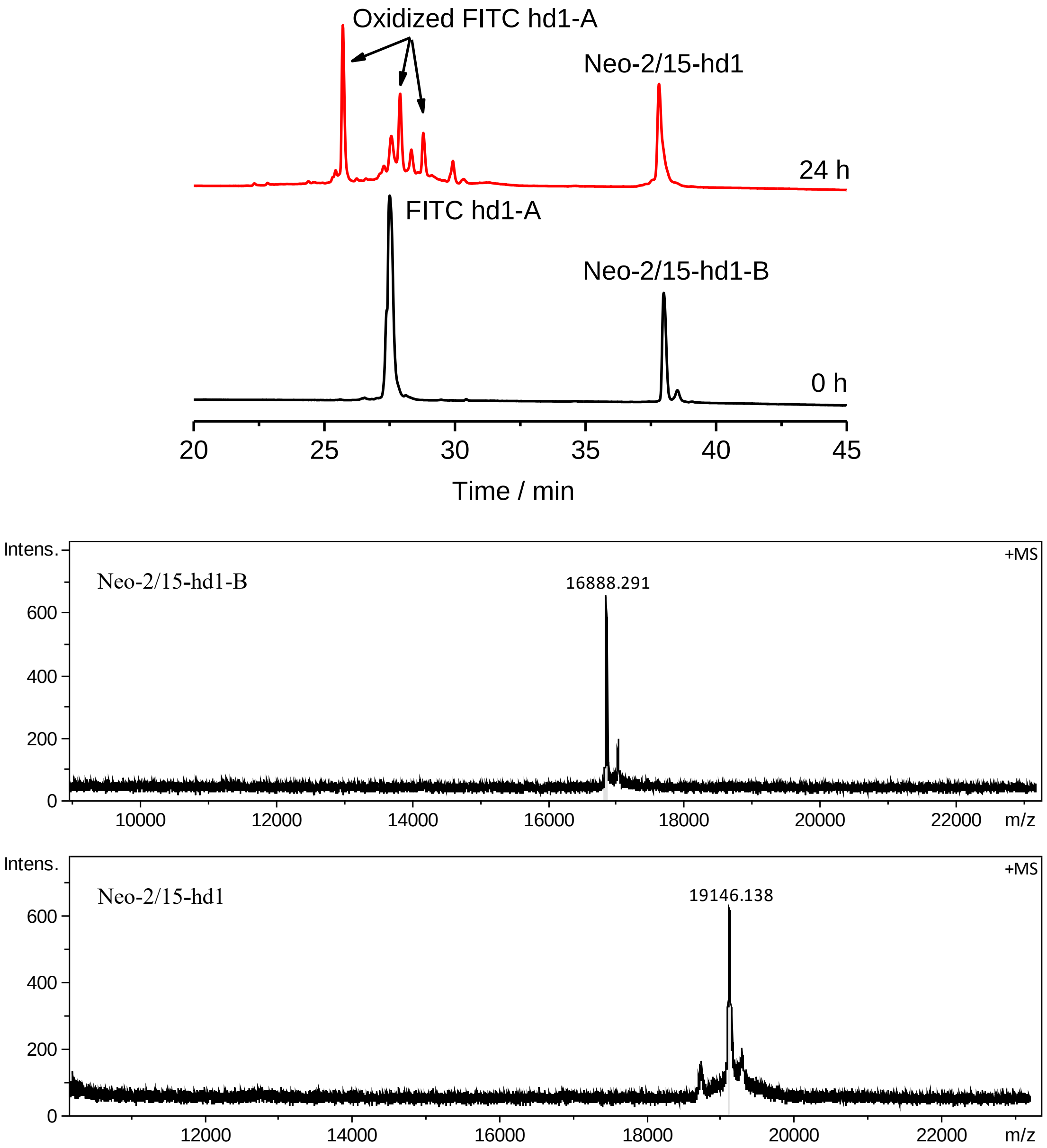

b)

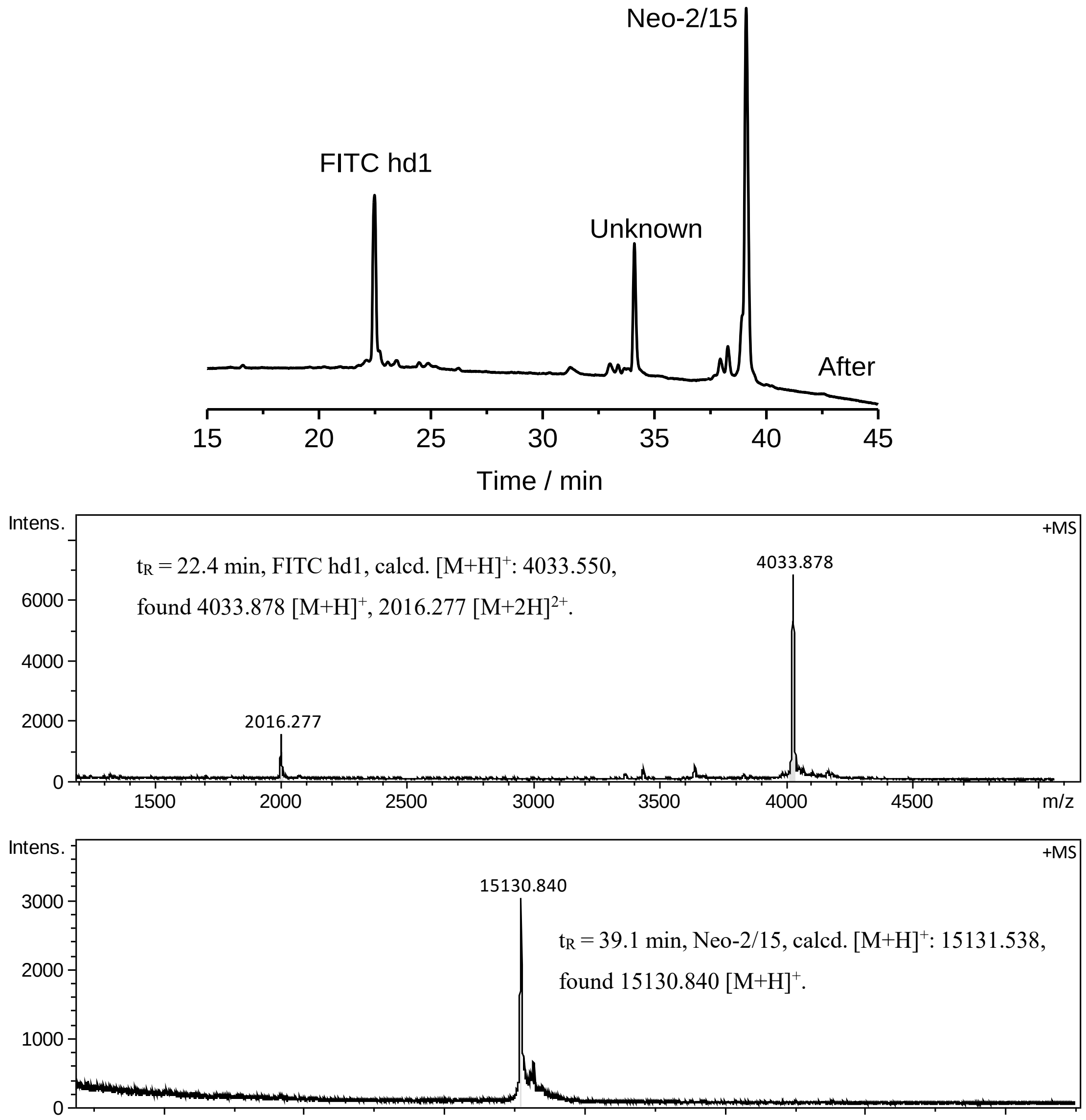

Supplementary Figure S39. Labeling of Neo-2/15 with fluorescent probe (FITC hd1) through the heterodimerization. a) HPLC chromatograms and mass spectra showing the formation of the labeled Neo-2/15. A: Pen-bearing monomer, B: Cys-bearing monomer. Reaction conditions: 100 µM FITC hd1-A, 12.5 µM Neo-2/15-hd1-B, 50 µM SeCys, 100 mM Gly-NaOH buffer (pH 10.0), at 37 °C for 24 hours. **b)** HPLC chromatogram and mass spectra showing the digested fragments of the labeled Neo-2/15 after treating with AcTEV^{TM} Protease. Unknown peak is not generated from the labeled protein.

a)

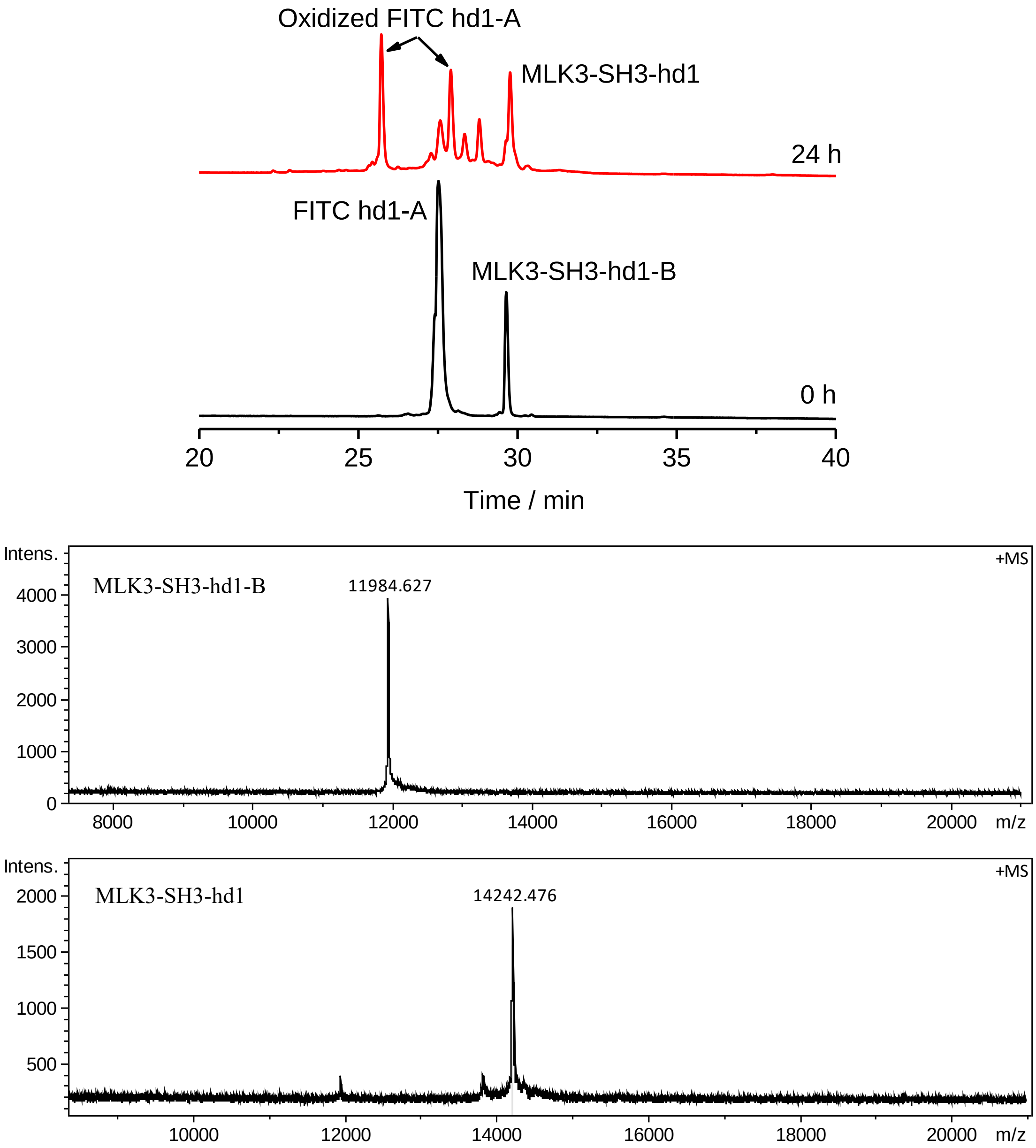
Oxidized FITC hd1-A
MLK3-SH3-hd1
24 h
FITC hd1-A
MLK3-SH3-hd1-B
0 h
20
25
30
35
40
Time / min
Intens.
+MS
MLK3-SH3-hd1-B
11984.627
4000
3000
2000
1000
0
8000
10000
12000
14000
16000
18000
20000
m/z
Intens.
+MS
MLK3-SH3-hd1
14242.476
2000
1500
1000
500
10000
12000
14000
16000
18000
20000
m/z

b)

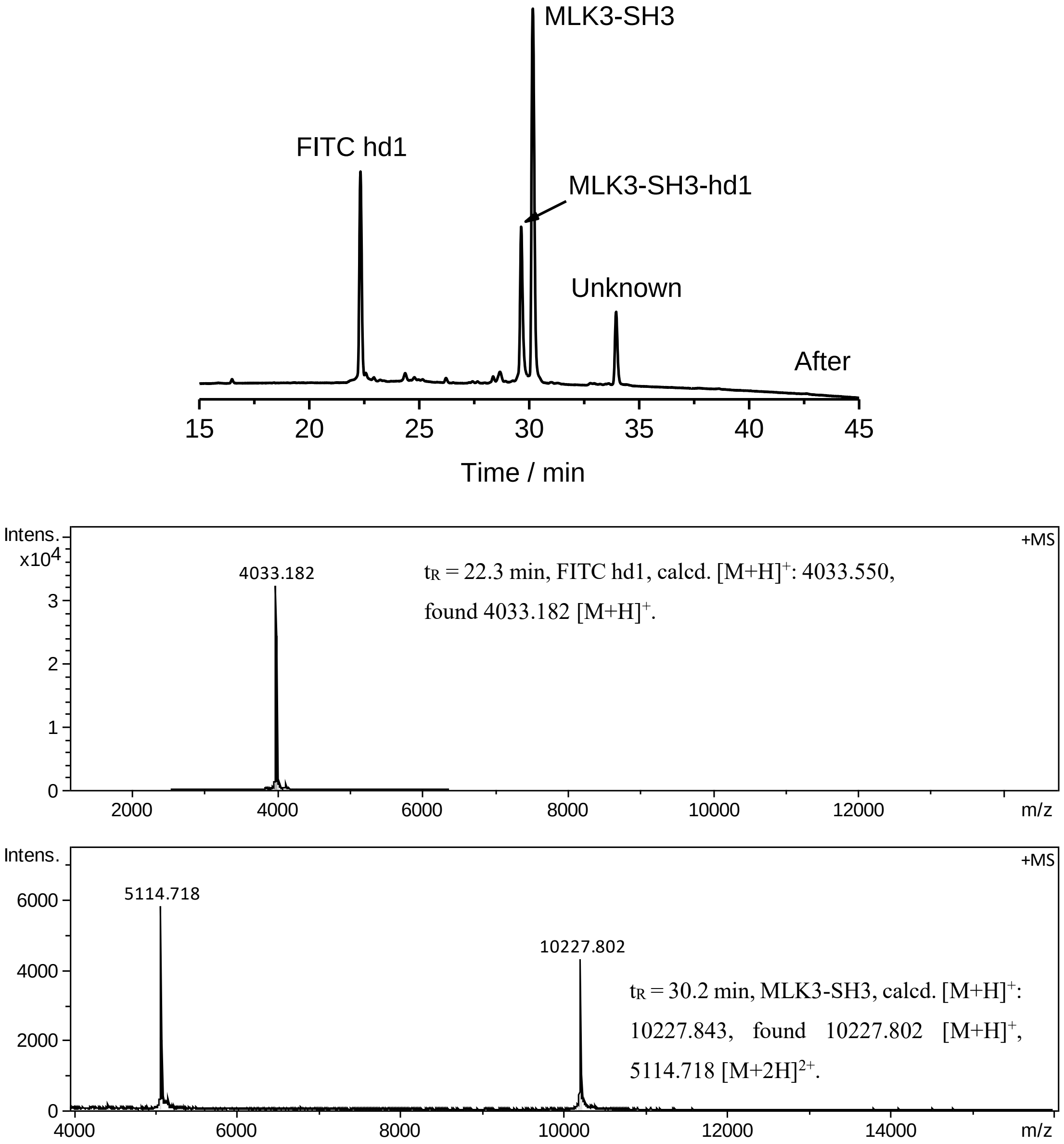

Supplementary Figure S40. Labeling of MLK3-SH3 with fluorescent probe (FITC hd1) through the heterodimerization. a) HPLC chromatograms and mass spectra showing the formation of the labeled MLK3-SH3. A: Pen-bearing monomer, B: Cys-bearing monomer. Reaction conditions: 100 μM FITC hd1-A, 5 μM MLK3-SH3-hd1-B, 50 μM SeCys, 100 mM Gly-NaOH buffer (pH 10.0), at 37 °C for 24 hours. **b)** HPLC chromatogram and mass spectra showing the digested fragments of the labeled MLK3-SH3 after treating with AcTEV^{TM} Protease. Unknown peak is not generated from the labeled protein.

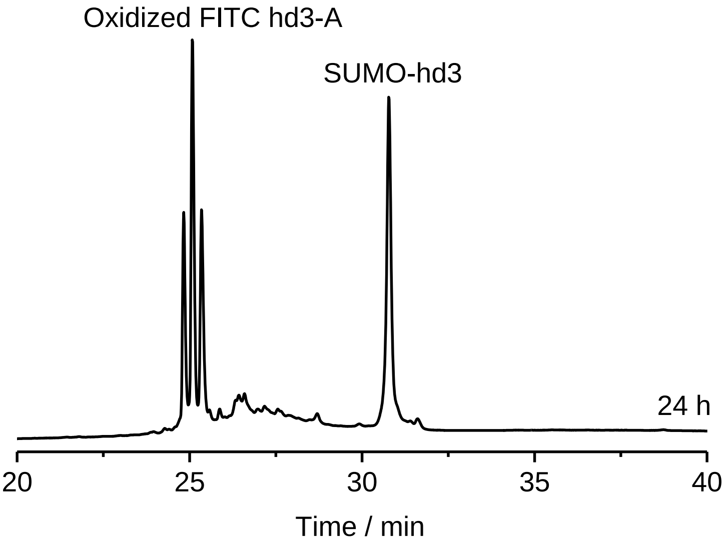

Oxidized FITC hd3-A
SUMO-hd3
24 h
20
25
30
35
40
Time / min

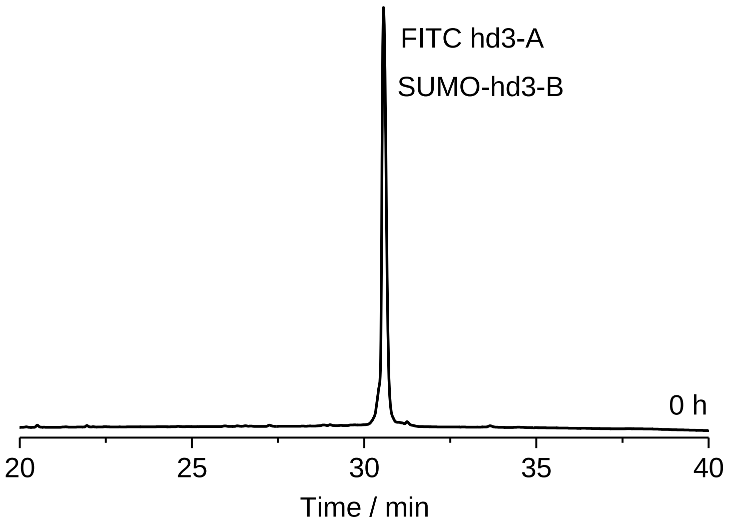

FITC hd3-A
SUMO-hd3-B
0 h
20
25
30
35
40
Time / min

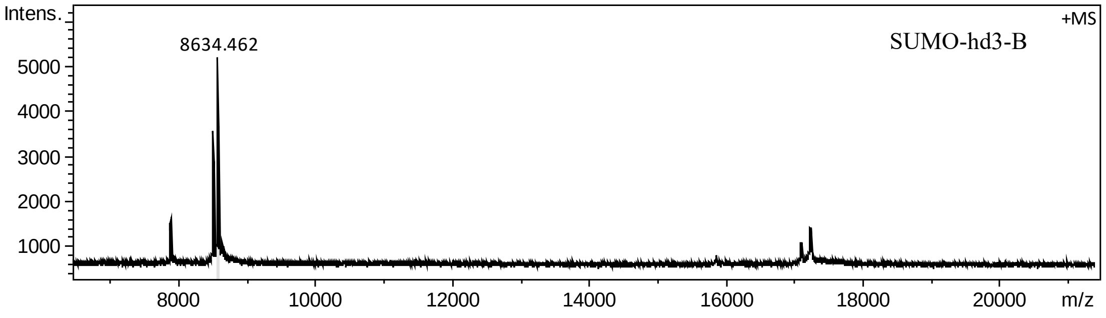

Intens.
+MS
8634.462
SUMO-hd3-B
5000
4000
3000
2000
1000
8000
10000
12000
14000
16000
18000
20000
m/z

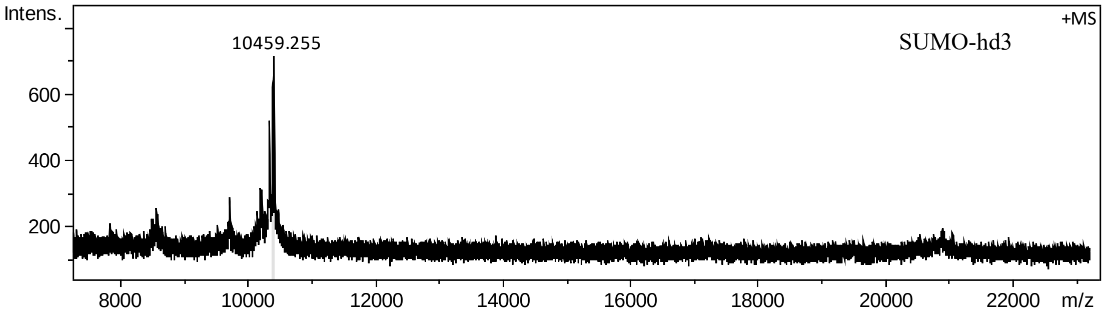

Intens.
+MS
10459.255
SUMO-hd3
600
400
200
8000
10000
12000
14000
16000
18000
20000
22000
m/z

Supplementary Figure S41. HPLC chromatograms and mass spectra showing the labeling of SUMO with fluorescent probe (FITC hd3) through the heterodimerization. A: Pen-bearing monomer, B: Cys-bearing monomer. Reaction conditions: 45 μM FITC hd3-A, 5 μM SUMO-hd3-B, 22.5 μM SeCys, 100 mM Gly-NaOH buffer (pH 10.0), at 37 °C for 24 hours.

a)

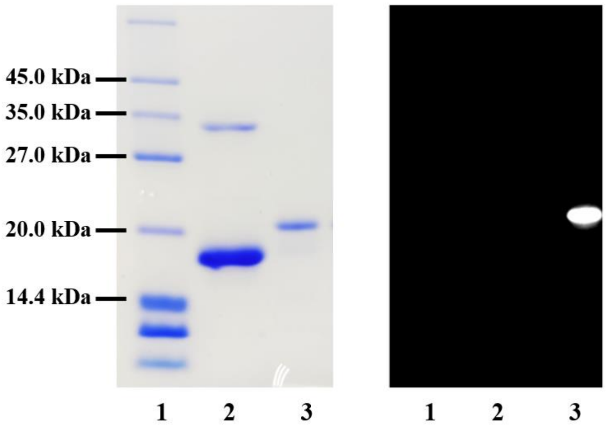

b)

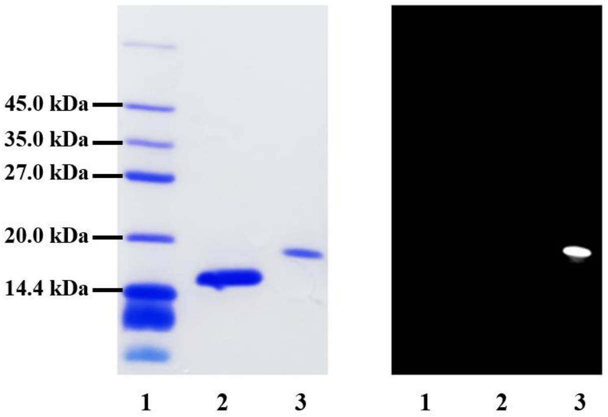

c)

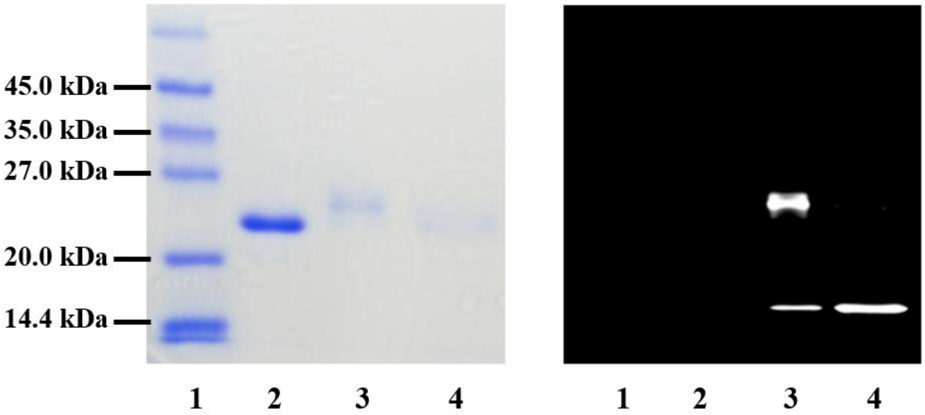

Supplementary Figure S42. White light (left) and fluorescence (right) images of a gel loaded with solutions containing different proteins (lane 1: markers; lane 2: unlabeled proteins; lane 3: fluorescence labeled proteins; lane 4: fluorescence labeled proteins treated with TCEP) and stained with Coomassie Blue. **a)** Neo-2/15-hd1. **b)** MLK3-SH3-hd1. **c)** SUMO-hd3. Experiments were repeated independently once with similar results using new samples. These results are reproducible.

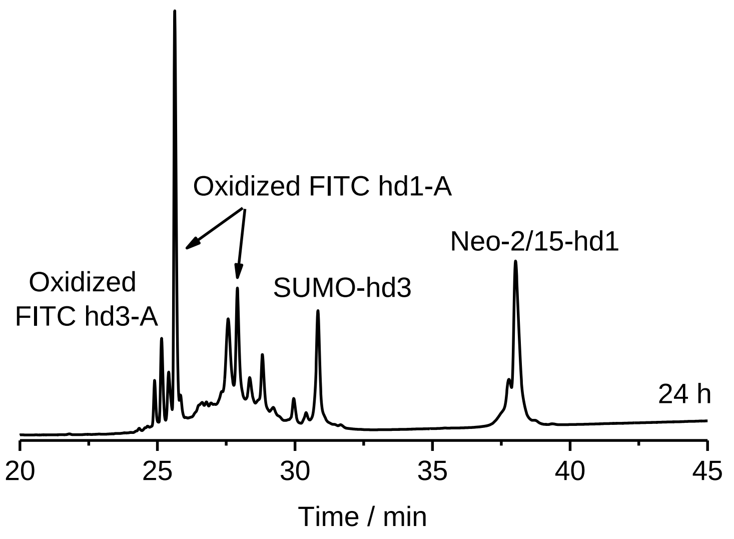

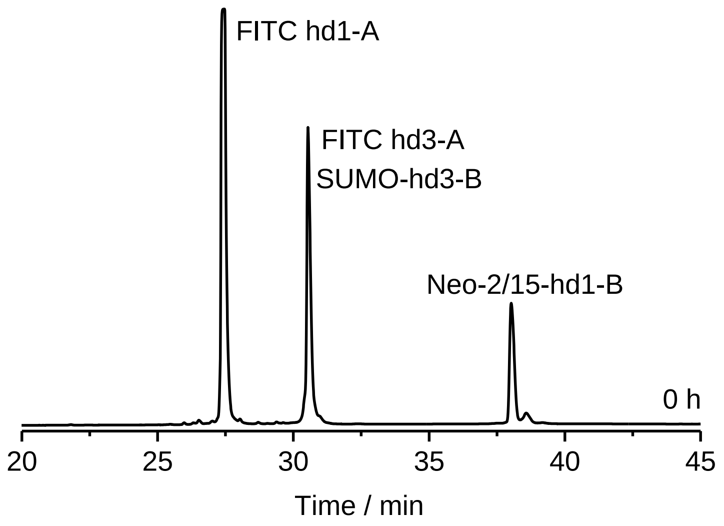

Supplementary Figure S43. HPLC chromatograms showing the labeling of Neo-2/15 and SUMO simultaneously through two orthogonal peptide heterodimerizations. A: Pen-bearing monomer, B: Cys-bearing monomer. Reaction conditions: 100 µM FITC hd1-A, 45 µM FITC hd3-A, 12.5 µM Neo-2/15-hd1-B, 5 µM SUMO-hd3-B, 72.5 µM SeCys, 100 mM Gly-NaOH buffer (pH 10.0), at 37 ℃ for 24 hours.

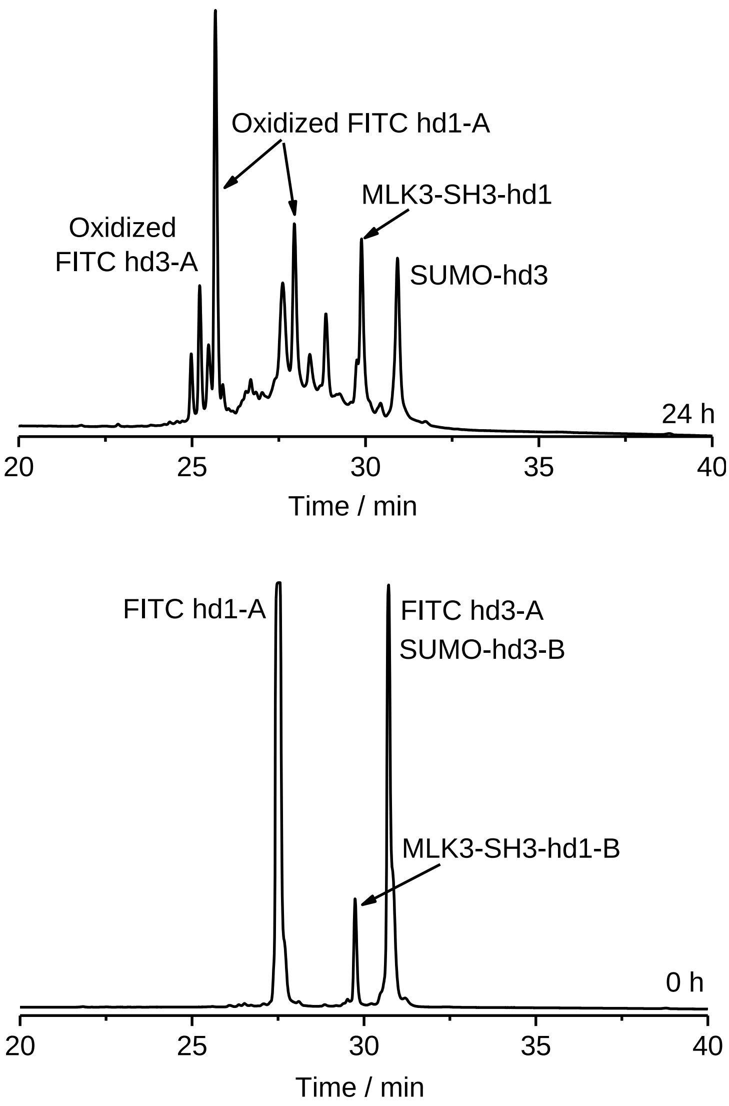

Supplementary Figure S44. HPLC chromatograms showing the labeling of MLK3-SH3 and SUMO simultaneously through two orthogonal peptide heterodimerizations. A: Pen-bearing monomer, B: Cys-bearing monomer. Reaction conditions: 100 μM FITC hd1-A, 45 μM FITC hd3-A, 5 μM MLK3-SH3-hd1-B, 5 μM SUMO-hd3-B, 72.5 μM SeCys, 100 mM Gly-NaOH buffer (pH 10.0), at 37 °C for 24 hours.

a)

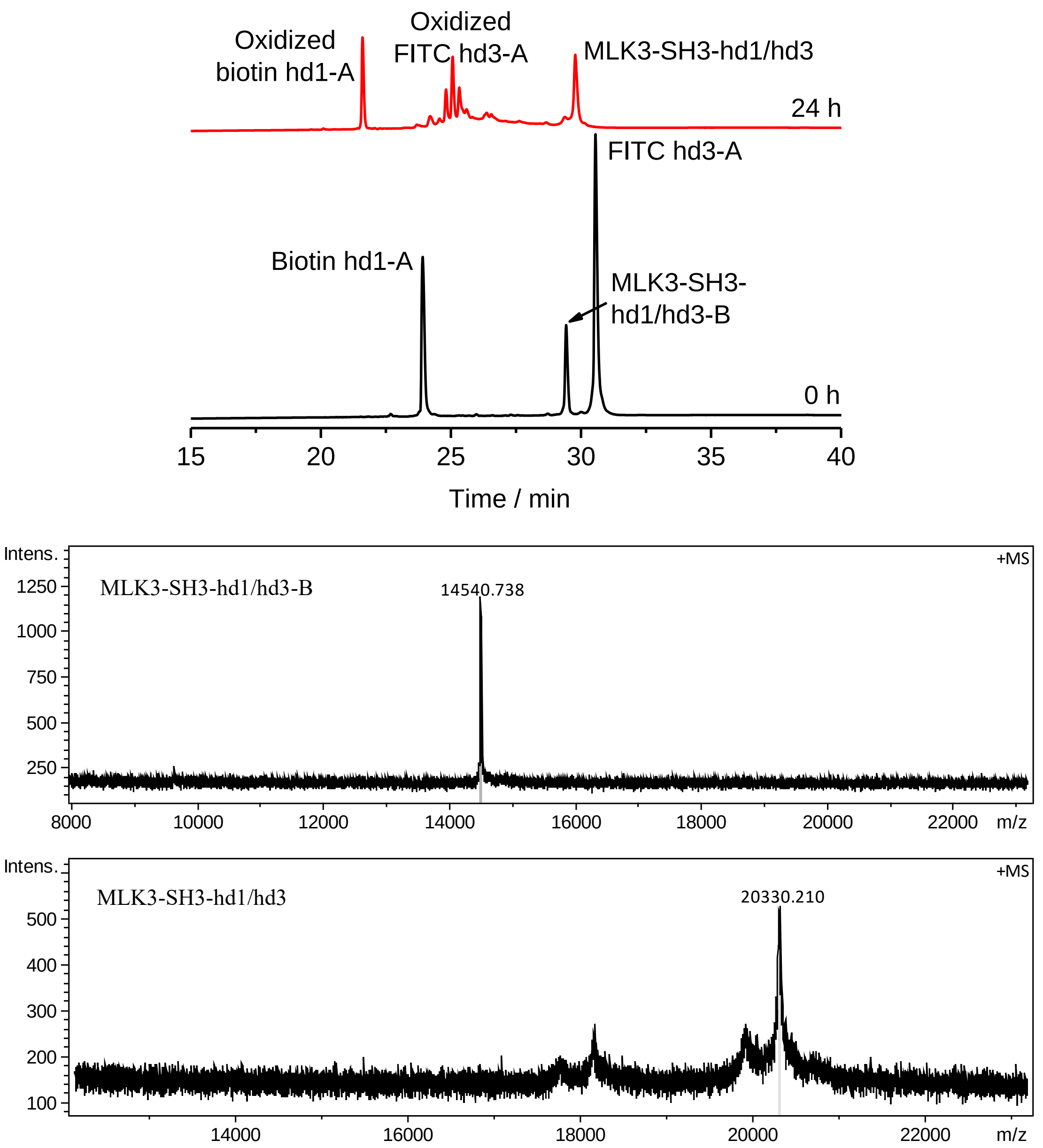

Oxidized biotin hd1-A
Oxidized FITC hd3-A
MLK3-SH3-hd1/hd3
24 h
FITC hd3-A
Biotin hd1-A
MLK3-SH3-hd1/hd3-B
0 h
15
20
25
30
35
40
Time / min
Intens.
1250
1000
750
500
250
MLK3-SH3-hd1/hd3-B
14540.738
+MS
8000
10000
12000
14000
16000
18000
20000
22000
m/z
Intens.
500
400
300
200
100
MLK3-SH3-hd1/hd3
20330.210
+MS
14000
16000
18000
20000
22000
m/z

b)

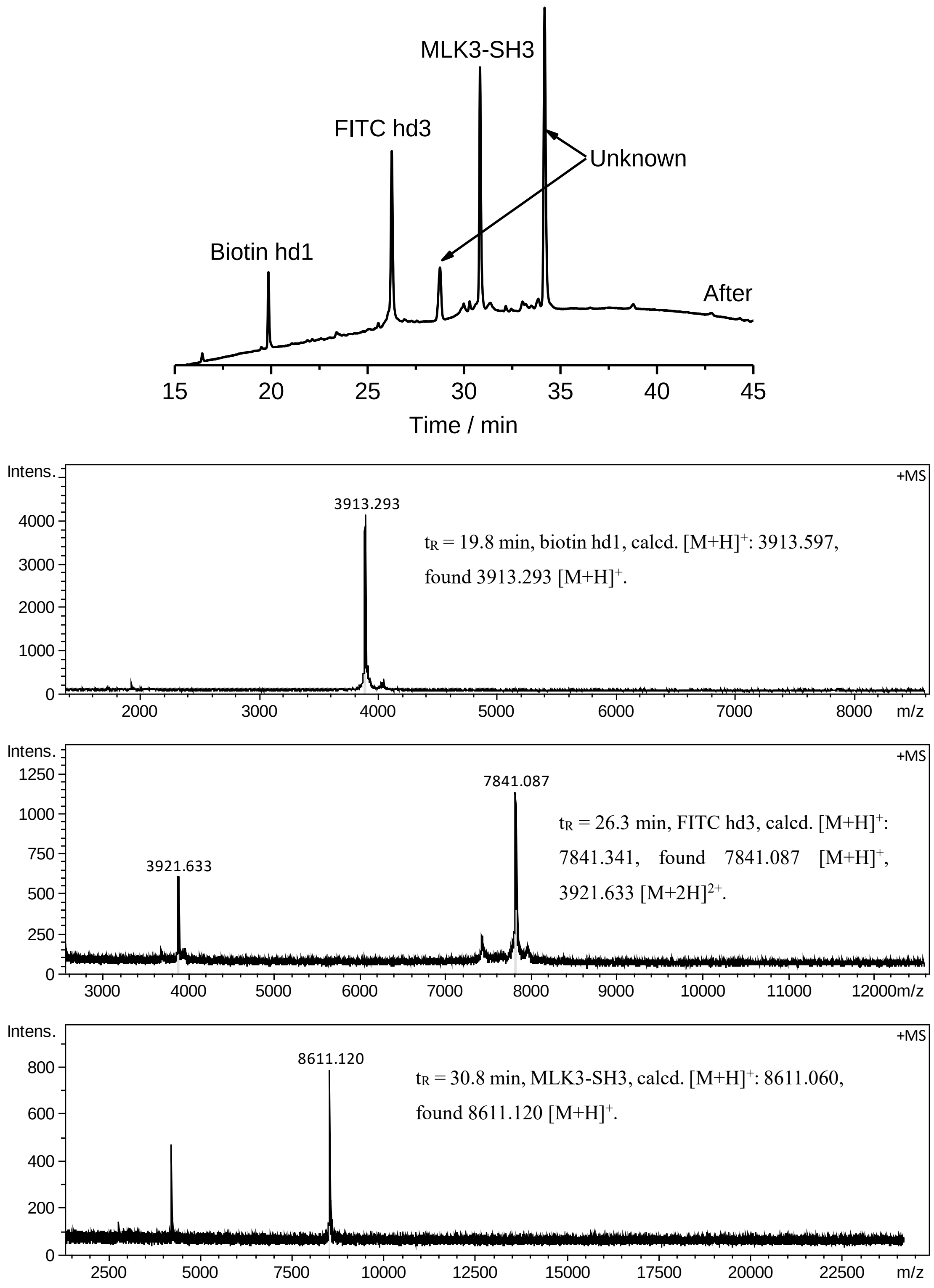

Supplementary Figure S45. Double labeling of MLK3-SH3 with two different functional modules through two orthogonal peptide heterodimerizations. a) HPLC chromatograms and mass spectra showing the formation of the doubly-labeled MLK3-SH3. A: Pen-bearing monomer, B: Cys-bearing monomer. Reaction conditions: 100 μM biotin hd1-A, 45 μM FITC hd3-A, 5 μM MLK3-SH3-hd1/hd3-B, 72.5 μM SeCys, 100

mM Gly-NaOH buffer (pH 10.0), at 37 °C for 24 hours. **b)** HPLC chromatogram and mass spectra showing the digested fragments of the doubly-labeled MLK3-SH3 after treating with AcTEV^{TM} Protease. Unknown peaks are not generated from the labeled protein.

2. References

1 Zhang, H. Y., Neal, S. & Wishart, D. S. RefDB: A database of uniformly referenced protein chemical shifts. *J Biomol. Nmr. 25*, 173-195 (2003).
