# Supplementary material for: De novo design and directed folding of disulfide-bridged peptide heterodimers
Source: Nat Commun. 2022 Mar 22;13:1539. doi: 10.1038/s41467-022-29210-x (PMC8941120; doi:10.1038/s41467-022-29210-x)
Supplement: Supplementary file 2 — Reporting Summary [file 41467_2022_29210_MOESM2_ESM.pdf]

nature portfolio

Corresponding author(s): Chuanliu Wu
Last updated by author(s): Feb 28, 2022

Reporting Summary

Nature Portfolio wishes to improve the reproducibility of the work that we publish. This form provides structure for consistency and transparency in reporting. For further information on Nature Portfolio policies, see our Editorial Policies and the Editorial Policy Checklist.

Statistics

For all statistical analyses, confirm that the following items are present in the figure legend, table legend, main text, or Methods section.

n/a	Confirmed	
☐	☒	The exact sample size (*n*) for each experimental group/condition, given as a discrete number and unit of measurement
☐	☒	A statement on whether measurements were taken from distinct samples or whether the same sample was measured repeatedly
☒	☐	The statistical test(s) used AND whether they are one- or two-sided<br>*Only common tests should be described solely by name; describe more complex techniques in the Methods section.*
☒	☐	A description of all covariates tested
☒	☐	A description of any assumptions or corrections, such as tests of normality and adjustment for multiple comparisons
☒	☐	A full description of the statistical parameters including central tendency (e.g. means) or other basic estimates (e.g. regression coefficient) AND variation (e.g. standard deviation) or associated estimates of uncertainty (e.g. confidence intervals)
☒	☐	For null hypothesis testing, the test statistic (e.g. *F*, *t*, *r*) with confidence intervals, effect sizes, degrees of freedom and *P* value noted<br>*Give P values as exact values whenever suitable.*
☒	☐	For Bayesian analysis, information on the choice of priors and Markov chain Monte Carlo settings
☒	☐	For hierarchical and complex designs, identification of the appropriate level for tests and full reporting of outcomes
☒	☐	Estimates of effect sizes (e.g. Cohen's *d*, Pearson's *r*), indicating how they were calculated

Our web collection on statistics for biologists contains articles on many of the points above.

Software and code

Policy information about availability of computer code

Data collection	Acquisition of UV-traces (HPLC chromatograms): LabSolutions version 5.85 (Shimadzu)<br>Acquisition of mass spectra: flexControl version 3.4 (Bruker), otofControl version 4.0.15.3248 (Bruker) and trapControl version 7.0 (Bruker)<br>Acquisition of circular dichroism spectra: Spectra Manager version 1.52.01 (JASCO)<br>Computational design: Rosetta software suite 3<br>Acquisition of NMR spectra: Topspin 3.5 (Bruker)<br>Code availability: The code used for the placement of disulfides into designed structures and instructions can be found at: https://github.com/atom-moyer/stapler. A representative script and associated parameter files used to design the peptides can be found in a Supplementary software file. The Rosetta software suite is available for free to all non-commercial users and to commercial users for a fee: https://www.rosettacommons.org/.
Data analysis	Topspin 3.5 was used to process NMR data.<br>NMRFAM-SPARKY was used for analysis of NMR spectra.<br>Xplor-NIH version 2.53 was used for NMR structure determination and refinement.

For manuscripts utilizing custom algorithms or software that are central to the research but not yet described in published literature, software must be made available to editors and reviewers. We strongly encourage code deposition in a community repository (e.g. GitHub). See the Nature Portfolio guidelines for submitting code & software for further information.

Data

Policy information about availability of data

All manuscripts must include a data availability statement. This statement should provide the following information, where applicable:

- Accession codes, unique identifiers, or web links for publicly available datasets
- A description of any restrictions on data availability
- For clinical datasets or third party data, please ensure that the statement adheres to our policy

The NMR structure data generated in this study have been deposited in the PDB database under accession code 7FB8 and 7FBA. The source data underlying Figure 1, Figures 3-5, Supplementary Figures 1 and 2, Supplementary Figures 6-21, Supplementary Figures 26-45 are provided as a Source Data file. PDB output files for these designed peptides are provided in a Supplementary data file. Any other data are available from corresponding authors upon reasonable request. Source data are provided with this paper.

Field-specific reporting

Please select the one below that is the best fit for your research. If you are not sure, read the appropriate sections before making your selection.

☒ Life sciences ☐ Behavioural & social sciences ☐ Ecological, evolutionary & environmental sciences

For a reference copy of the document with all sections, see nature.com/documents/nr-reporting-summary-flat.pdf

Life sciences study design

All studies must disclose on these points even when the disclosure is negative.

Sample size	No sample size calculation was performed, but experiments were repeated independently at least once using a new sample to validate the result.
Data exclusions	No data were excluded for the analyses.
Replication	Experiments were repeated independently at least once with similar results. All results were reproducible
Randomization	Experiments were not randomized, but data were validated by replication.
Blinding	Investigators were not blinded to the samples that were measured, but data were validated by replication.

Reporting for specific materials, systems and methods

We require information from authors about some types of materials, experimental systems and methods used in many studies. Here, indicate whether each material, system or method listed is relevant to your study. If you are not sure if a list item applies to your research, read the appropriate section before selecting a response.

Materials & experimental systems

n/a	Involved in the study	
☒	☐	Antibodies
☒	☐	Eukaryotic cell lines
☒	☐	Palaeontology and archaeology
☒	☐	Animals and other organisms
☒	☐	Human research participants
☒	☐	Clinical data
☒	☐	Dual use research of concern

Methods

n/a	Involved in the study	
☒	☐	ChIP-seq
☒	☐	Flow cytometry
☒	☐	MRI-based neuroimaging
