# Supplementary material for: De novo design and directed folding of disulfide-bridged peptide heterodimers
Source: Nat Commun. 2022 Mar 22;13:1539. doi: 10.1038/s41467-022-29210-x (PMC8941120; doi:10.1038/s41467-022-29210-x)
Supplement: Supplementary file 3 — Description of additional supplementary files [file 41467_2022_29210_MOESM3_ESM.pdf]

Description of Additional Supplementary Files

File name: Supplementary software

Description: This zip archive contains a representative script and associated parameter files used to design the peptides in the article.

File name: Supplementary Data

Description: This zip archive contains the PDB output files from Rosetta for all designed peptides reported in the article.
